# 1D/2D Heterostructures: Synthesis and Application in Photodetectors and Sensors

**DOI:** 10.3390/nano14211724

**Published:** 2024-10-29

**Authors:** Yuqian Liu, Yihao Lin, Yanbo Hu, Wenzhao Wang, Yiming Chen, Zihui Liu, Da Wan, Wugang Liao

**Affiliations:** 1 School of Information Science and Engineering, Wuhan University of Science and Technology, Wuhan 430081, China; 2 Engineering Research Center of Metallurgical Automation and Measurement Technology, Wuhan University of Science and Technology, Wuhan 430081, China; 3 State Key Laboratory of Radio Frequency Heterogeneous Integration (Shenzhen University), College of Electronics and Information Engineering, Shenzhen University, Shenzhen 518060, China

**Keywords:** 1D/2D heterostructures, synthesis, photodetector, sensor

## Abstract

Two-dimensional (2D) semiconductor components have excellent physical attributes, such as excellent mechanical ductility, high mobility, low dielectric constant, and tunable bandgap, which have attracted much attention to the fields of flexible devices, optoelectronic conversion, and microelectronic devices. Additionally, one-dimensional (1D) semiconductor materials with unique physical attributes, such as high surface area and mechanical potency, show great potential in many applications. However, isolated 1D and 2D materials often do not meet the demand for multifunctionality. Therefore, more functionality is achieved by reconstructing new composite structures from 1D and 2D materials, and according to the current study, it has been demonstrated that hybrid dimensional integration yields a significant enhancement in performance and functionality, which is widely promising in the field of constructing novel electronic and optoelectronic nanodevices. In this review, we first briefly introduce the preparation methods of 1D materials, 2D materials, and 1D/2D heterostructures, as well as their advantages and limitations. The applications of 1D/2D heterostructures in photodetectors, gas sensors, pressure and strain sensors, as well as photoelectrical synapses and biosensors are then discussed, along with the opportunities and challenges of their current applications. Finally, the outlook of the emerging field of 1D/2D heterojunction structures is given.

## 1. Introduction

In the last few years, with the continuous progress and development of science and technology, the fabrication and application of one-dimensional/two-dimensional (1D/2D) heterostructures has taken place. Optoelectronic devices and flexible electronics have shown rapid growth in momentum. Regarding flexible electronics, 1D/2D heterostructures are extensively utilized in wearable gadgets, smart sensors, and foldable screens [1,2,3,4]. Their excellent flexibility enables these heterostructures to adapt to various complex surfaces, providing greater flexibility and possibilities for the creation and production of electronic equipment. In the field of optoelectronic devices, 1D/2D heterostructures also show great potential. These structures can be used not only in order to create extremely effective solar cells and photodetectors but also for applications such as photonic devices and optoelectronic modulators. Through rational design and the optimization of these structures, high performance and the stability of optoelectronic devices can be achieved, bringing new opportunities and challenges for the development of optoelectronic technology.

In summary, 1D/2D heterostructures possess a wide range of potential uses in the field of electronic and optoelectronic devices, which will inject new vitality and momentum into scientific and technological innovation and industrial development. We look forward to more research and practice in the future to further explore and exploit the potential value of these structures and promote the continuous progress and improvement of related technologies.

Before exploring the application prospects, we must also recognize that challenges still exist regarding the preparation of high-quality and stable 1D/2D heterostructure systems. Firstly, problems such as the controllability of material growth and the formation of interfacial defects need to be overcome during the preparation process. Secondly, understanding and optimizing the formation mechanism and physical properties of 1D/2D heterostructures is also a complex subject. This involves in-depth studies and analyses of the structure, electrical properties, and optical properties of the materials. To overcome these challenges, there have been some representative attempts to work on them. For example, researchers have endeavored to improve the quality and stability of one-dimensional/two-dimensional heterostructures by regulating the growth conditions and optimizing the material structure and interfacial engineering. At the same time, they have also explored the formation mechanism and physical properties of 1D/2D heterostructures through a combination of experiments and theoretical simulations, providing a deeper understanding of their applications.

Overall, despite the challenges faced by 1D/2D heterostructure systems, its promising applications in electronic and optoelectronic apparatus are still highly anticipated. The timeliness of this review lies in the systematic summary of the latest research progress and application prospects, which provides an important reference and guidance for researchers in related fields. Its uniqueness lies in the in-depth and comprehensive discussion of the preparation, mechanism, and application of 1D/2D heterostructures, which highlights their importance in the future evolution of science and technology.

**Figure 1:**
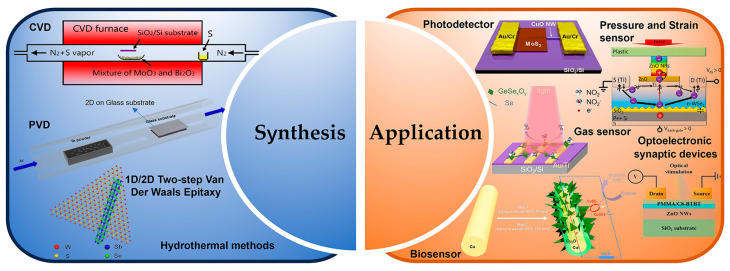
1D/2D heterostructures: synthesis and application in photodetectors and sensors. Reproduced with permission from the authors of [5,6,7,8,9,10,11,12].

This review is divided into the following sections: First, the preparation methods and material properties of 1D/2D heterostructures will be introduced. Then, the formation mechanism and physical properties of 1D/2D heterostructures will be highlighted. Then, the applications of 1D/2D heterostructures in photodetectors and sensors will be introduced in detail, and their future development direction will be prospected. Finally, the challenges and solutions of 1D/2D heterostructure systems will be summarized, and their outlook given consideration (Figure 1). In addition, for the convenience of readers to quickly review, we listed some synthesis methods and characteristics of materials (Table 1). And, we presented some properties and applications of one-dimensional/two-dimensional heterostructures (Table 2). 

## 2. Synthesis

### 2.1. Chemical Vapor Deposition (CVD)

CVD denotes the procedure of generating solid deposits by reacting gaseous or vaporous substances at the gas phase or gas–solid interaction at a certain temperature and pressure, which is divided into three important stages: the diffusion of reactive gases to the surface of the substrate; the chemical process between the reactive gases and the substrate’s surface; and the growth of deposits on the surface of the substrate and separation of gas phase byproducts from the surface of the substrate.

CVD has received extensive attention from researchers due to its advantages of simple preparation process, controllability, good film forming effect, and adjustable parameters. At present, CVD technology has been widely used and developed in many industries; for example, in the semiconductor industry, it is used to grow thin films of silicon, silicon nitride, and other materials and prepare transistors, light-emitting diodes (LEDs), photovoltaic cells, and other devices; in the field of biomedicine, it is used in biosensors, biomedical coatings, and other aspects. In the next section, we will outline the preparation of 1D, 2D, and 1D–2D materials by CVD and discuss the specific techniques, advantages, and areas to be improved for the preparation of materials of different dimensions using CVD.

#### 2.1.1. 1D

CVD can be used to prepare many types of one-dimensional nanostructures, such as nanowires, nanorods, nanotubes, etc., in which a wide range of materials can be prepared, including metals, semiconductor compounds, etc. One-dimensional nanostructures prepared by CVD possess an extensive range of uses, and their important applications are in sensor technology, optoelectronic devices, catalysts, energy storage and conversion, nanoelectronics, and nanobiology. In the last few years, researchers have accomplished a certain degree of precise control over the growth process of 1D structures by adjusting the reaction conditions, optimizing the catalysts, and selecting suitable precursors, thus obtaining semiconductor materials with good morphology and structure and then exploring the physical properties of semiconductors under different structures.

**Figure 2:**
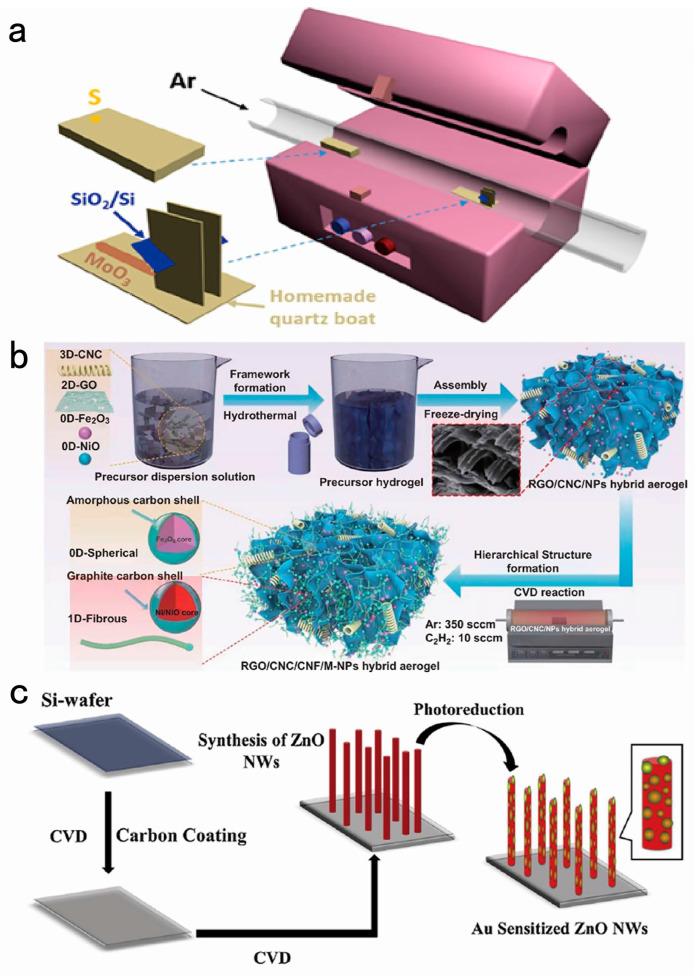
(**a**) Schematic representation of the setup for the growth of MoS_2_ nanobelts. The S powder is positioned upstream of the MoO_3_-loaded quartz boat, which is positioned in the center of the quartz tube. Reproduced with permission from the authors of [39]. (**b**) Complete synthesis process of the graphene oxide (RGO)/carbon nanocoil (CNC)/carbon nanofiber (CNF)/metal oxide nanoparticles (M-NPs) hierarchical aerogel. Reproduced with permission from the authors of [41]. (**c**) Schematic diagram of the growth of Au-sensitized ZnO NWs on silicon wafers. Reproduced from [44] with permission from the authors.

Xu et al. mixed 2 mg of powder (Te:W:NaCl = 16:1:0.1) with the agate mortar; the powder was sandwiched by two Si/SiO_2_ substrate, then put in a CVD system; finally, the furnace cooled down to room temperature naturally, and the WTe_2_ 1D nanoribbons (NRs) were obtained on the substrate [38]. Similarly, Yang et al. synthesized MoS_2_ nanobelts on a SiO_2_/Si substrate with a homemade quartz boat using a chemical vapor deposition (CVD) method. The schematic illustration of the setup for the growth is shown in Figure 2a [39].

Tuzluca et al. successfully synthesized one-dimensional single crystal In_2_O_3_ nanotowers, nanobeams, nanocones, and nanowires in a CVD system [40]. Yesilbag et al. used a CVD system for multilayer graphene (MLG) synthesis in a vacuum environment. Using varying growing times and methane (CH_4_) gas flow rates, six experiments were conducted for MLG synthesis [13].

When Zhao et al. conducted the experiment, the process was conducted at 450 °C, while introducing 350 sccm and 10 sccm of Ar and C_2_H_2_ gases. Aerogels with different electromagnetic properties can be prepared with different reaction times (Figure 2b) [41]. The following are also used to prepare aerogels.

Zou et al. prepared carbon nanotube fibers using an injection chemical vapor deposition (iCVD) method. Here, the synthesis is also referred to as a floating catalyst CVD. The special feature of this synthesis process is that a mist of ethanol, ferrocene (2 wt.%), and thiophene (1 vol%) is injected into a heated gas flow reactor along with the injection of carrier gas, which induces the detachment of the grown carbon nanotubes in the form of aerogels, which are further obtained in fibrous form by passing through a water bath [42]. The injection chemical vapor deposition is a special type of CVD in that the “injection” process involves injecting precursor solutions or gases into the system by means of an injection device. The precursors are usually selected to have catalytic ability to assist in the growth of the material. In CVD, the precursors are usually introduced into the reaction chamber through a gas flow control system. Comparatively speaking, the injection chemical vapor deposition has a higher process control accuracy, resulting in higher deposition uniformity and film quality, which gives it an advantage in the preparation of high-purity, low-defect films, as well as lower deposition temperatures. In addition, initiated chemical vapor deposition, also referred to as iCVD, is a special class of gas phase polymerization technology based on free radical polymerization. In the general initiated chemical vapor deposition process, monomers and initiators are introduced into a reactor, wherein the initiators are contacted with heated filaments and thermally dissociated to form free radicals, which are physically adsorbed to the substrate by the free radical polymerization of the monomers on the surface. This growth characteristic makes the polymer film virtually independent of the substrate or surface. Polymer films synthesized by plasma enhancement or pyrolysis-based CVD are produced by the direct decomposition of monomers and are prone to defects and byproducts. This is effectively avoided by the solvent-free nature of the iCVD process and the unique film formation mechanism. Overall, this iCVD has significant advantages over conventional CVD in terms of temperature control and film quality. Gozde Ozaydin Ince et al. have explored the great potential of iCVD for successful applications by generating one-dimensional surface-imprinted polymer nanotubes within anodized aluminum oxide films based on initiated chemical vapor deposition [43].

Kang et al. prepared ZnO nanofibers on silicon wafers by chemical vapor deposition. ZnO nanowires (NWs) were created by evaporating zinc wire at 700 °C for 30 s with a 0.6 liter per minute (LPM) flow rate of carrier gas (Figure 2c) [44]. The experiment of Zou et al. using zinc as the preparation material is as follows: ZnO powder (99.99%) and graphite powder were mixed in 1:1 or 1:2 molar ratio, which was used as the growth source. ZnO microwires were synthesized using CVD at an atmospheric pressure [14]. Unlike the previous two experiments where Nb was doped in the precursor solution, niobium-doped zinc oxide (NZO) thin films were ultimately obtained. The precursor solutions were made by dissolving 0.5 g of Zn(CH_3_COO)_2_·2H_2_O and various amounts of Nb (OCH_2_CH_3_)_5_ in either anhydrous methanol (99.8%, Sigma) or methanol (99.6%, Sigma-Aldrich) at a temperature of 400 °C for all depositions. Finally, Shaukat et al. obtained niobium-doped zinc oxide (NZO) thin films [45]. 

#### 2.1.2. 2D

Two-dimensional materials have received widespread attention due to their exceptional physical attributes, such as high mobility, adjustable band gap, excellent mechanical ductility, and other vast application potential in the field of flexible devices, optical–electronic transition, microelectronic devices, etc. They are very likely to become one of the materials to replace traditional semiconductors, but they also face great challenges in material synthesis, processing, and integration. In contrast to the traditional mechanical stripping method to obtain two-dimensional materials, this method is limited by the quality and size of the material and other factors: the yield of the finished products is low and cannot achieve the control of the number of layers. The liquid phase exfoliation method can achieve low-cost 2D material preparation of larger quantities, but there are still challenging to regulate the product size and other issues. All of the above methods are “top-down” 2D materials from raw materials; the process is random, uncontrollable, and easy to be contaminated in the preparation process. CVD, on the other hand, has a unique “bottom-up” preparation principle, which is significantly better than earlier synthesis methods in terms of controllability and the ability to avoid contamination during the preparation process. In addition, CVD has the advantages of uniform film formation, fast speed, simple process operation, and no pollution to the environment, which makes it among the most effective techniques for the preparation of 2D semiconductor materials and is particularly appropriate for wide-ranging practical applications.

The use of different preparation methods will make the thickness, size, defects, crystallinity, and other parameters of the two-dimensional materials change, which will have a certain impact on their properties. In order to attain superior quality and the controllable preparation of 2D materials, the experimental parameters in the growth process, such as the kind and dosage of precursors, the growth temperature, and the pressure in the growth system, the type and the carrier gas’s flow rate, etc., are often altered to achieve the precise regulation of the quantity of layers, stages, and morphology of 2D semiconducting materials. In order to flexibly control these growth parameters, researchers have proposed a number of interesting ideas: the “face-to-face” method, in which the substrate is inverted to face the source material directly to achieve the formation of the source material vapor clusters in space; the inverted structure of the quartz ship which achieves the uniform, low-rate diffusion of precursors; the confined-space CVD system, which provides a stable laminar flow; a physical filter structure, which essentially covers the precursor with alumina microsphere powder uniformly (alumina is used here as an example, and the filter material should actually be selected according to the probe material), and the publication of precursor vapor is controlled by changing the particle size of the alumina microsphere powder to achieve the precursor feed rate; and the countercurrent strategy, which prevents the unintended supply of chemical vapor sources and eliminates uncontrolled nucleation.

The primary precursors of most transition metal disulfide compounds have high melting temperatures, making them difficult to synthesize using conventional CVD procedures. The main solution is to adopt transition metal chlorides with lower melting points as metal–metal precursors, as they have high evaporation rates and high chemical activity, which are very favorable for synthesis, or to use a combination of alkali halides and metal oxides as metal precursors, where the intermediate products generated by the reaction between the two can lower the metal’s melting point or metal oxide and enhance the overall reaction rate.

Achieving the controllable preparation of 2D materials opens up scalable avenues in order to obtain high-performance devices.

**Figure 3:**
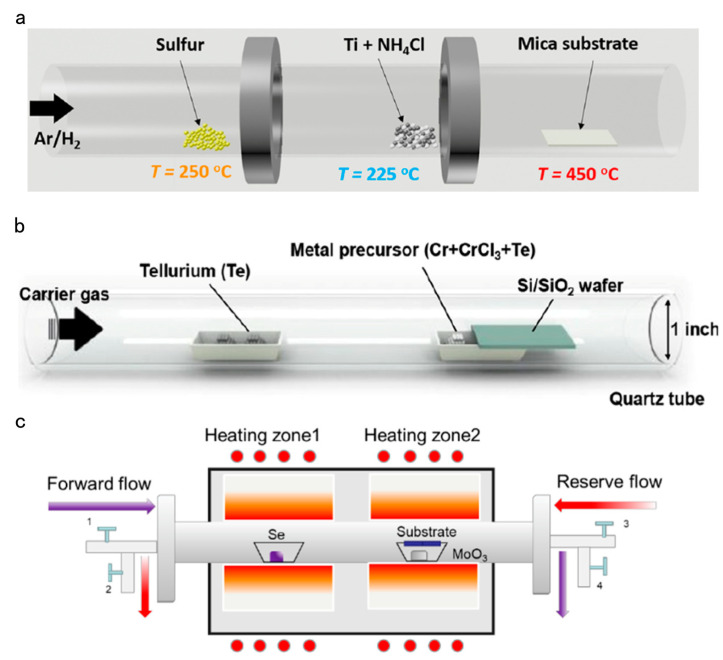
(**a**) Configuration of the experiment for the CVD development of mica-substrate-based TiS_2_ nanosheets. Reproduced from [18] with permission from the authors. (**b**) Diagram showing the experimental configuration used to create CrTe nanoflakes. Reproduced with permission from the authors of [50]. (**c**) Illustrative diagram of dual-zone furnace chemical vapor deposition device. The direction of gas flow is switched by turning on/off gas valves 1 and 4 or 2 and 3. Reproduced with permission from the authors of [52].

Wu et al., Bosi and Rotunno, Gong et al., and Liu et al. grew MoS_2_ thin films in single and dual temperature zone CVD furnaces. They all placed sulfur powder in a quartz boat upstream of a tube furnace, with a slightly different Mo source. Wu et al. and Liu et al. used MoO_3_ powder, Bosi and Rotunno used MoOx and MoS_2_ powder, and Gong et al. used ammonium molybdate ((NH_4_)_2_MoO_4_) as the molybdenum source [16,46,47,48]. They all raised the temperature of the furnace from room temperature to 450–850 °C at a certain heating rate and maintained it for a period of time. During the synthesis process, a fixed flow of Ar, Ar/H_2_, or N_2_ was introduced as the carrier gas. After growth, the furnace was allowed to naturally cool to ambient temperature, and finally, MoS_2_ thin films were obtained on the target substrate. Hafeez et al. synthesized double-layer ReS in a quartz tube with a horizontal furnace with three zones as described above. The precursor materials were ReO_3_ and S, and the carrier gas, high-purity N_2_, was used for deoxygenation in the furnace, followed by heating and maintaining the highest temperature for 10 min at a constant flow of high-purity N_2_ and H_2_, followed by natural cooling [49]. Dai et al. also used sulfur powder as a precursor to place it in the upstream hot zone, another hot zone downstream of the nickel dichloride powder. Under a constant flow rate of Ar and H_2_ composed of ultrahigh-purity argon gas, the two hot zones were heated and maintained for 10 min to grow naturally and cool down. Finally, nickel sulfide nanosheets were obtained [17]. Similar to the three experiments mentioned above, Gao et al. employed chemical vapor deposition to grow TiS_2_ nanosheets and thin films, with sulfur powder and Ti/NH_4_Cl powder as the precursors (Figure 3a) [18]. Guo et al. used mixed compounds of Cr, CrCl_3_·6H_2_O, and Te powder as the metal precursors to successfully grow CrTe nanosheets using CVD (Figure 3b) [50]. Meng et al. also used Te as the precursor. Different from the above experiment, MoO_3_ powder was used, in which NaCl was added as the reagent to promote the MoO_3_ reaction, and finally, MoTe_2_ film was synthesized [51]. Wang et al. used MoO_3_ (15 mg, Aladdin powder, 99.95%) and selenium (300 mg, Aladdin powder, 99.95%) powder as the precursors to introduce Ar to wash the gas pipe and remove oxygen, heated from room temperature to the required growth temperature, and Ar/H_2_ mixture as the carrier gas to finally grow MoSe_2_. The MoSe_2_ was grown in a dual-zone furnace with 2-inch quartz tubes and a home-improved CVD system (see Figure 3c) [52]. 

CVD is an important method to prepare high-quality and large-scale 2D materials, but the defects such as vacancies and grain boundaries produced have a significant impact on the materials’ inherent qualities during the preparation process. Additionally, the prepared size still has a large gap compared with the actual demand, and the controlled preparation of the highest quality and large-area 2D materials is still one of the current challenges. At present, there are fewer temporary research reports on the crystal growth mechanism of complex structures, and the reaction process of synthesizing 2D materials using CVD is plagued with obstacles, such as difficulty in controlling it, instability, and fluctuating growth parameters.

#### 2.1.3. 1D/2D

Heterostructures constructed from semiconductor materials have important application prospects in the field of semiconductor devices, providing a basis for the development of new functional materials and devices. Quantum well, as a two-dimensional heterojunction structure with energy grading and carrier confinement, is widely utilized in optoelectronic devices, including lasers and photodiodes. Quantum wire, as a one-dimensional heterojunction structure with quantum confinement effects, has important applications in nanoelectronics and quantum computing. Quantum dots, as zero-site heterojunction structures with size confinement effects, have special optical, electrical, and magnetic properties and are widely used in optoelectronic devices, quantum dot displays, bio-imaging, and other fields. Layers of 2D materials form another low-dimensional heterojunction structure that can modulate the electrical, optical, and mechanical properties of the materials, offering a wide range of possibilities for novel electronic devices, sensors, and energy storage.

In order to pursue the integration of 2D heterojunctions with the existing semiconductor industry, it is crucial to utilize CVD to attain large-scale controllable growth of high-quality and high-yield 2D materials. Currently, recent studies based on the CVD growth of TMD transverse heterojunctions, TMD vertical heterojunctions, and TMD Moore superlattices have led to a certain degree of controllability over the size, stacking method, and number of layers of various 2D heterostructures. In contrast, it is still challenging for the assembly of 1D/2D heterostructures due to the imperfect mechanism of 1D/2D heterostructures. Defect-induced conucleation growth, which will be mentioned below, is a critical extension method for 1D/2D heterostructures.

**Figure 4:**
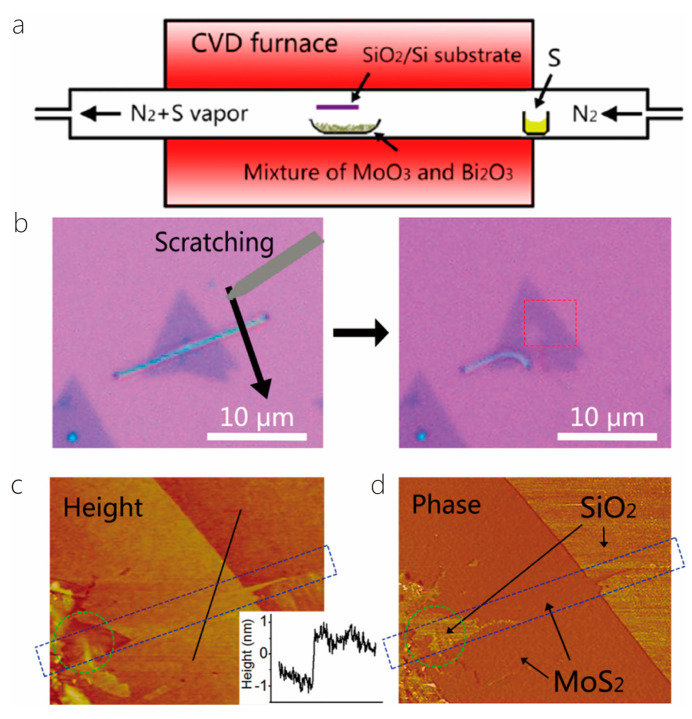
(**a**) Illustrative diagram of the heterogeneous structure synthesis process, in which a mixture of MoO_3_ and Bi_2_O_3_ operated as a solid source and heatedly interacted with S vapor. (**b**) Comparison of optical images of typical heterostructures before and after probe scratching, where a portion of the nanowires were scraped off by the probe, exposing the nucleation region below. (**c**) The AFM height image is in the enlarged red frame region (**b**). The green dotted ring marks the nucleation region, and the blue dotted box indicates the region covered by the Bi_2_S_3_ nanowires. The small figure at the bottom right also shows the height distribution along the black line, indicating no connection between the nanowires and the MoS_2_ monolayer other than the nucleation site. (**d**) The AFM phase image is in the same region in (**c**). The area selected by the green dashed circle clearly shows the lower SiO_2_ substrate after the Bi_2_S_3_ nanowires have been scraped off by part of the adjacent MoS_2_ nanosheet after being connected at the nucleation site. Reproduced with permission from the authors of [5].

Qian et al. also synthesized MoS_2_ single-walled carbon nanotube (MoS_2_-SWCNT) heterostructures using the CVD method. Based on the gas phase decomposition of ferrocene in a CO atmosphere, SWCNT thin films were synthesized using the gas phase chemical vapor deposition method. Then, the SWCNT thin films were transferred to selected graphite substrate pores using the dry transfer technique. MoO_3_ and S powder were used as the precursors to synthesize MoS_2_-SWCNT thin films in a system of low-pressure CVD. S powder was placed on an upstream quartz boat and heated to 100 °C–130 °C, while MoO_3_ was placed on a different quartz boat next to S and heated to 530 °C. Steam was introduced into the SWCNT thin films in the center of the CVD furnace through a 50 sccm Ar flow, maintaining the synthesis temperature at 530 °C with a typical growth time of 50 min [53]. According to the above synthesis steps, 2D MoS_2_ can be grown on 1D SWCNT. Li et al. also synthesized 1D-Bi_2_S_3_/2D-MoS_2_ heterostructures using the CVD method. Bi_2_O_3_ and MoO_3_ sand particles were placed in a horizontal quartz tube furnace in the focus of a quartz boat, and a hygienic SiO_2_/Si substrate was inverted on top of the solid source. Another small quartz ship loaded with pure sulfur was placed upstream of the cooling zone 12 cm away from the substrate. Then, a large flow of pure N_2_ was introduced into the quartz tube expelling the air inside the pipe. Throughout the time of the growth process, the nitrogen flow was maintained at 20 sccm, and the furnace temperature was warmed to 650 °C within 16 min, maintaining the growth of Bi_2_S_3_/MoS_2_ heterostructures for 6 min (Figure 4) [5].

How to control the growth direction of heterostructures in CVD is an important prerequisite to achieving great performance equipment. Due to the complexity of nucleation factors in the CVD process, precise nucleation control is also an important goal of the research, because it is difficult to realize selective epitaxy, and it is necessary to combine it with the growth theory of crystals to select the most appropriate growth strategy to grow ideal 2D heterostructures. However, the crystal growth mechanism of complicated structures has been not been sufficiently reported for the time being and needs to be improved continuously. Moreover, there has not been much research carried out on complicated structures’ crystal formation mechanisms up to this point, and this needs to be improved continuously.

### 2.2. Physical Vapor Deposition (PVD)

PVD technology refers to the process of vaporizing gaseous atoms or molecules on the surface of a material source, whether it be liquid or solid, or partially ionizing them into ions under vacuum conditions and depositing a thin film with a particular function on the surface of the substrate by means of a low-pressure gas (or plasma); it is among the primary technologies for surface treatment. PVD methods mainly include the techniques of vacuum evaporation, sputtering deposition, and ion plating. Among these, thermal evaporation is a technique used for depositing thin films. In a vacuum chamber, the source material is heated and evaporated into a gas using an electron beam or resistance wire, and the gaseous source material will directly adhere to the substrate that was positioned on the upper side of the raw material without colliding with the background atmosphere. Under common atmospheric pressure (10^−4^ Pa), the average free path of particles with a size of 0.4 nm is approximately 60 nm. The above techniques have wide applications in the preparation of 1D–2D heterostructure materials, for example within the domain of optoelectronic, sensor, and nanoelectronic devices. Interface control and material compatibility are the key issues in the preparation of 1D–2D heterostructures, and the pursuit of good lattice matching and chemical stability at the heterojunction interface is an important way to effectively reduce interfacial defects and property degradation. Large area, high uniformity, and repeatability are the main challenges faced by PVD, especially in heterostructures, where parameters such as the deposition rate and the thickness of different materials need to be strictly controlled. However, as more and more novel 1D structures and 2D materials are explored and studied, more in-depth research on their growth mechanisms will continue to optimize the preparation scheme, which has great potential in the synthesis of new materials.

#### 2.2.1. 1D

The PVD synthesis process has a distinct impact on the morphology of nanostructures, in which the factors regulating the nanostructures mainly include the synthesis temperature, component vapor pressure, and growth pressure. Additionally, the controllable growth of a nanomaterial structure is a challenge that needs to be improved. Thermal evaporation, the main technology of the PVD approach, is extensively used in the arranging of semiconductor materials on account of its high purity, simple operation, and low cost. In practice, 1D semiconductor materials need to be grown according to the design pattern and, therefore, have high requirements on their size, orientation, and uniformity. The structure of the synthesis can be explored by regulating the process parameters during thermal evaporation. Sometimes the morphology of 1D semiconductor materials can be studied by thermal evaporation, which may require the assistance of catalysts or additives. During the thermal evaporation process, the catalyst content can be adjusted to investigate the structural changes, and the catalyst can be used to help the material grow as desired. However, sometimes a catalyst-free thermal evaporation process is required, considering that 1D semiconductor materials may be integrated with silicon-based technologies and that metal catalysts may affect their properties. A detailed development of the above is described below.

**Figure 5:**
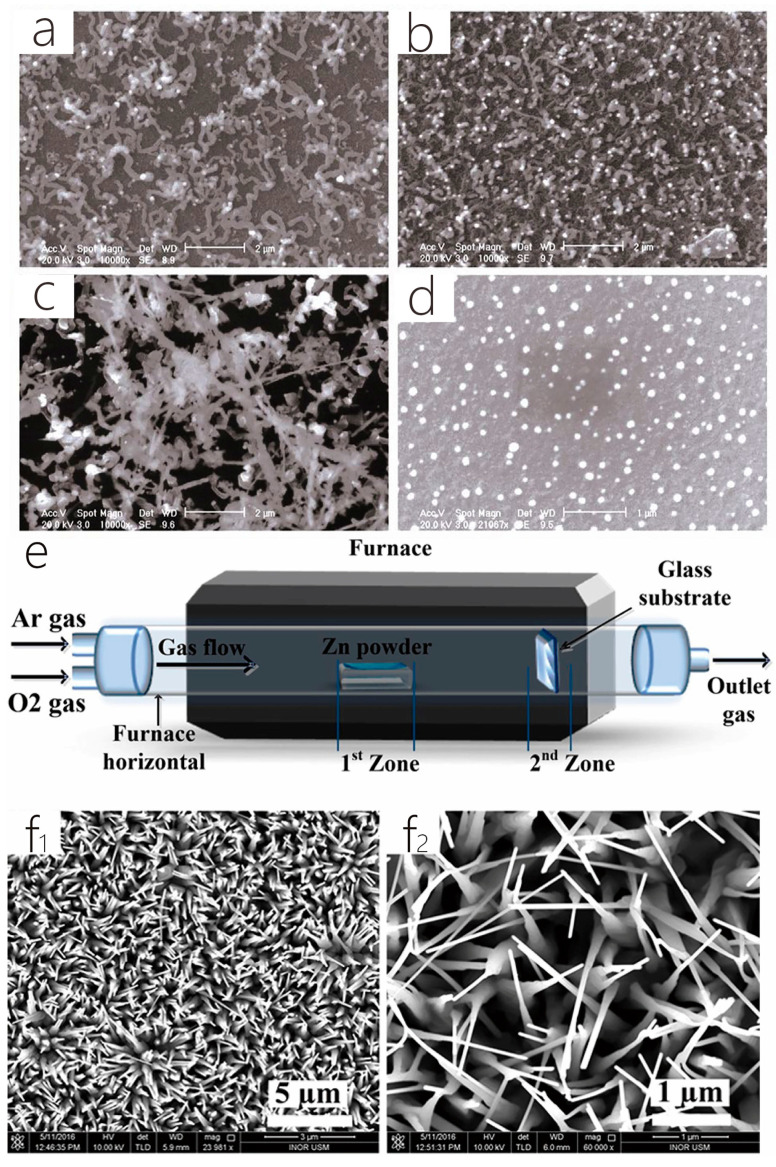
(**a**–**d**) SEM images of ZnS nanostructures grown at different temperatures: (**a**) 500 °C (**b**) 460 °C (**c**) 800 °C (**d**) 850 °C. Reproduced with permission from the authors of [19]. (**e**) Schematic diagram of the setup used for the growth of ZnO nanoneedles by thermal evaporation method. (**f_1_**,**f_2_**) Low and high magnification of FESEM images of the ZnO nanoneedles grown under 20 mL/min Ar flow rates. Reproduced with permission from the authors of [15].

In the experiments of Jin and Ge et al., quartz tubes were installed in a tube furnace, using zinc sulfide powder as the raw material, and a silicon substrate was placed downstream. Then, the pressure was kept at 10^−1^ Pa, and ZnS powder was brought to 850 °C within 1.5 h and kept at this temperature for 2 h. At the same time, the substrate temperature during the deposition region was maintained at about 500 °C–780 °C to synthesize the one-dimensional ZnS nanostructures (Figure 5a–d shows ZnS nanostructures captured in SEM pictures at various temperatures) [19]. Under a similar procedure, Jin and Peterson et al. synthesized Zn_0.9_Cd_0.1_S one-dimensional nanotubes (NTs) using a mixture of zinc sulfide and cadmium sulfide powders as raw materials and Au-coated Si wafers as substrates [54]. In another experiment, Alsultany et al. used a silver-plated glass substrate and changed the raw material to metallic zinc powder. The biggest difference was that high-purity argon was filled before heating, and high-purity oxygen was filled after heating. Figure 5(f_1_,f_2_) shows low- and high-magnification FESEM images of ZnO nanopins grown at 20 mL/min argon fluxes. After evaporation, a whitish substance was formed on the silver-plated glass substrate. Figure 5e depicts a conventional furnace with a horizontal quartz tube that has a 40 cm reaction zone, a 4 cm diameter, and a 70 cm length [15]. Similarly, Jin and Wei et al. loaded In powder and a Si substrate on a ceramic ship, which was also purified with high-purity Ar and O_2_, and finally, the In_2_O_3_ nanostructure was obtained [20]. Xie et al. added silicon powder to a crucible of graphite and fixed carbon fibers on top of the powdered silicon. After being evacuated to about 100 Pa, Ar (99.99%) was poured into the furnace until the overall pressure was 0.11 MPa. After heating and natural cooling, white cotton similar to nanostructures are finally obtained on carbon fibers [55]. Dontsova et al. underwent similar structural steps and ultimately obtained SnC_2_O_4_ powder in an alumina vessel to obtain SnO_2_ nanostructured materials [21]. Lu et al. placed the Si substrate coated with Au thin film diagonally 15 cm downstream of the ZnS powder and introduced argon gas before heating, reducing the pressure to 7.5 × 10^−2^ Torr and began heating but, ultimately, deposited at room temperature to obtain a white woolly product [56]. M. Shariati and V. Ghafouri used traditional thermal evaporation technology to prepare In_2_O_3_ nanorods and nanowires using metal indium and zinc in a horizontal tube furnace [57]. Wei et al. obtained large-scale single-oriented quasi-one-dimensional Te nanowires with an average length of ≈40 µm by growing PVD on an m-plane sapphire substrate. Te nanowires have high crystallinity and a clear chiral spiral chain structure, which endows them with strong anisotropy [58]. Li et al. also prepared Te nanowires using the PVD method. They used a pulse nucleation strategy to prepare ultrathin nanostructures using the pulsed PVD method, by controlling the nucleation and growth stages of oversaturation, respectively, to prepare the Te nanowires with a thickness of <10 nm [59]. Physical vapor deposition has been widely used in semiconductor electronics for high-quality crystal growth and thin-film deposition. The PVD method includes evaporation deposition, magnetron sputtering, and pulsed laser deposition and has a wide range of applicability. The PVD method can provide high-purity materials, suitable for extremely high-quality applications. It is still used in the synthesis of one-dimensional materials. 

Although the synthesis of the above materials using PVD makes it easy to control their structure, it should be noted that the means used for the synthesis may affect the type of defects and the density of defects in the 1D semiconductor materials, changing their physical properties either positively or negatively. The control of growth kinetics of 1D semiconductor materials is a crucial component in determining the formation of their nanostructures, but this area is complex and awaits progressive exploration.

#### 2.2.2. 2D

Two-dimensional materials, with their special structural and electrical characteristics, have a broad selection of possible applications in electronics, optoelectronics, energy, biomedicine, and other fields. In the past, the way to obtain two-dimensional materials was mostly using chemical or mechanical stripping, but this technology is subject to low yield, and the subsequent transfer process is susceptible to pollution, as well as other limitations. Physical vapor deposition, however, has the advantages of high-purity preparation and the precise control of film thickness, and it has become one of the most widely utilized ways to synthesize 2D materials. At present, the combination of extensive, high-quality 2D semiconductor materials has become one of the main directions of research. The following section firstly describes the specific way to synthesize 2D nanosheets using layered materials by physical vapor deposition, followed by a demonstration of the preparation of 2D nanosheets from nonlayered materials, which complements the range of applications of 2D materials. Finally, the focus is on distinct single-element 2D materials for which replicable and scalable large-area growth is a challenge.

**Figure 6:**
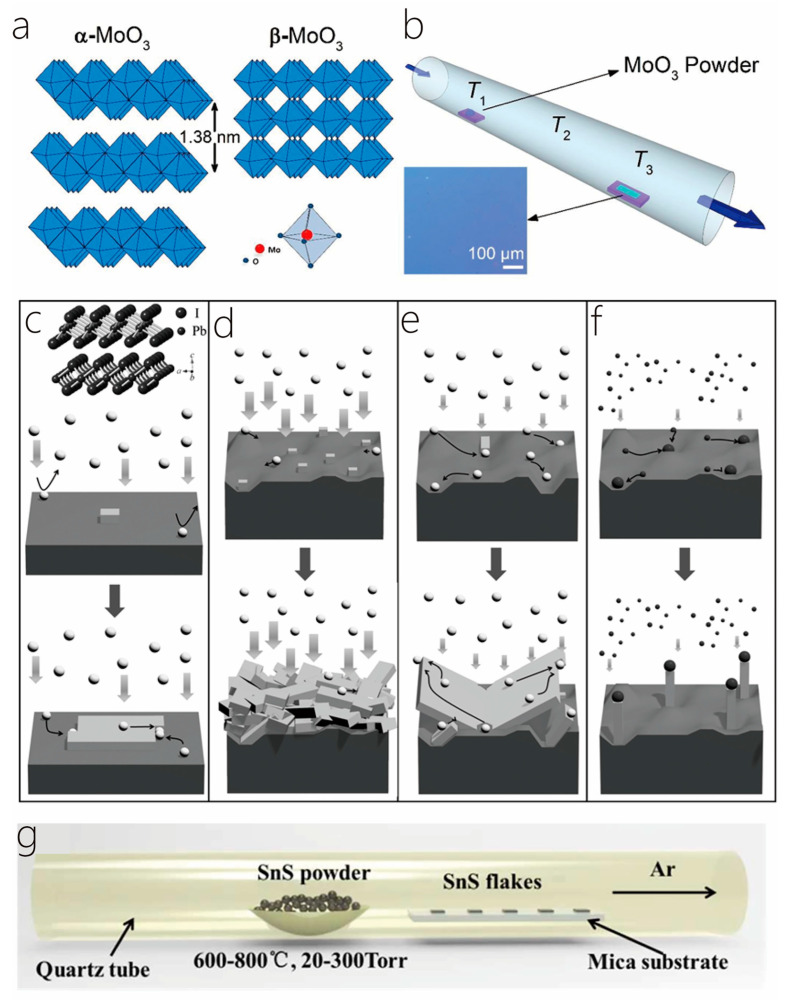
(**a**) A diagram illustrating the crystal structures of α- and β-MoO_3_. Atoms are denoted by red spheres for Mo and blue spheres for O. (**b**) Diagram illustrating the experimental setup for producing MoO_3−x_ homogeneous quasi-2D layers through thermal evaporation of MoO_3_ powder. The flow of Ar is indicated by blue arrows. Reproduced with permission from the authors of [61]. Diagrammatical representation of the growth kinetics and mechanisms of numerous PbI_2_ nanostructures obtained using different substrates and process conditions. (**c**) The growth behavior of 2D nanosheets on a smooth Si substrate is placed in a flat position. The illustration shows the atomic model of PbI_2_, demonstrating its layered structure and repeated I-Pb-I unit cells stacked along the c-axis. (**d**) At a relatively low process pressure, the figure shows the formation of high-density and tightly packed self-supporting nanosheets on the substrate with a rough surface. (**e**) As shown in the figure, the process pressure starts to increase, and the less dense, independent nanosheets form on the rough surface substrate. (**f**) The nanowires are grown on a rough-surface substrate with the highest process pressure. Reproduced with permission from the authors of [22]. (**g**) Schematic for PVD expansion of 2D SnS flakes. Reproduced with permission from the authors of [23].

Hanson et al. successfully synthesized high-quality, several-to-double layer thickness MoO_3_ using physical vapor deposition [60], during which MoO_3_ samples were obtained by using MoO_3_ powder as the precursor, 300 nm SiO_2_/Si as the substrate, ultradry air 18 sccm per minute as the carrier gas, and controlling the growth pressure, monothermal temperature, and time. Arash et al. chose the same precursor and substrate, but in order to achieve the optimum 310 m Torr of deposition pressure at a constant 100 sccm Ar flow, the quartz tube was evacuated, and the corresponding temperatures were set in each of the three temperature zones. The sample cooled down spontaneously to room temperature after 45 min of deposition. Figure 6a,b shows the crystal composition diagram of α-MoO_3_ and β-MoO_3_ and the schematic diagram of the experimental device [61]. Yao et al. placed a small ceramic boat containing 0.5 g of CuI powder as the precursor in the center of the quartz tube. A ceramic boat containing 0.5 g CuI powder as the precursor was positioned in the center of the quartz tube so that the SiO_2_/Si substrate was in another boat downstream about 10 cm from the center. γ-CuI nanosheets were grown using this homemade PVD system, expanding the study of 2D nanosheets prepared from nonlaminated materials [62]. Suleiman et al. synthesized ultrathin InI sheets using space-limited physical vapor deposition [63]. Lan et al. demonstrated the large-scale synthesis of free-standing PbI_2_ and MAPbI_3_ nanosheets. From the experiments, it can be concluded that highly crystalline, dense, and freestanding PbI_2_ nanosheets can be obtained relatively easily by intentionally utilizing the rough Si substrate surface under the optimal process pressure. Figure 6c–f shows the growth kinetics and mechanism of different PbI_2_ nanostructures under contrasting substrates and procedure conditions [22]. Xia et al. successfully synthesized large-scale two-dimensional (2D) orthorhombic SnS sheets by using freshly cut mica flakes as the substrate (Figure 6g) [23]. Zhou et al. placed a silica boat in the tube’s center with 20 mg of InSe powder, while a SiO_2_ wafer was placed downstream as a substrate, and 60 sccm of Ar was introduced to provide an inert atmosphere. The InSe powder was heated to 830 °C within 30 min and maintained at a constant temperature for a period of time to grow the InSe atomic layer [64]. Sharma et al. prepared large area fluorescent graphite oxide (FGO) films using the physical vapor deposition technique [65]. Chanana et al.’s experiments demonstrated the combination of high-quality large-area Cd_3_As_2_ films using thermal evaporation from commercial Cd_3_As_2_ lumination source material (American elements, Los Angeles, CA, USA, product #CD-AS-05-L), and crystal growth was observed at the optimized substrate temperature range of 95 °C–100 °C. During deposition, the chamber pressure was maintained at 3 × 10^−5^ Torr, and the average film deposition rate was 10.5 Å/s. The films were annealed in a three-zone tube furnace under an argon inert atmosphere, and the first zone’s temperature, which corresponds to the sample location was gradually heated (4 °C/min) to 450 °C. The samples were kept at this particular temperature for approximately 2 h and slowly cooled to room temperature in an Ar stream, approximately 2 °C/min [66].

Nowadays, research is not only satisfied utilizing the combination of high-quality, large-scale 2D semiconductor materials using PVD, but also focuses on regulating the process parameters, processing the substrate, and other operations to explore the growth morphology and the corresponding physical properties, which offers a solid material basis for the manufacture of high-performance electronic devices. However, the investigation of the growth mechanism of 2D materials is not extremely clear, and it is still difficult to obtain large-scale, uniform, low-layer, high-quality semiconductor materials.

#### 2.2.3. 1D/2D

The feature of no dangling bonds on the exterior of 2D materials allows for the incorporation of 2D materials with 1D materials to overcome the upper bound lattice mismatch, and also, this is one of the main motivations for the development of nano-heterostructures. In addition, by combining nanomaterials of different sizes, mixed-dimensional heterojunctions (MDHJs) take advantage of the desirable properties of their components, so heterojunctions composed of materials of different dimensions may have advantages such as synergistic effects and better interfacial bonding. Mixed-dimensional van der Waals (vdW) heterostructures tend to have higher light absorption cross-sections than full two-dimensional vdW heterostructures, which provide various opportunities for the growth of sensors, electronic devices, storing energy, and other fields. PVD, with the advantages of strong compatibility, good interfaces, and excellent adhesion, shows enormous possibility for application in the arrangement of 1D/2D heterojunctions. Among them, the thermal evaporation technique can be used to build one-dimensional/two-dimensional heterojunction structures using the stepwise evaporation of different materials to form heterojunction structures with specific functions. This is widely used in optoelectronic devices and sensors due to its advantages of mild reaction conditions, high crystallinity, and purity. Although the thermal evaporation technique is able to regulate the thickness and composition of the film more precisely and is expected to prepare multifunctional nanodevices, there are still challenges in controlling the depth of the film, which needs to be assisted by a precise monitoring and feedback system to guarantee the uniformity and reproducibility of the film. In addition, the synthetic treatment of certain materials requires high-temperature settings, which limits the choice of substrate materials. Process parameters still need to be optimized to obtain high-quality nanomaterials and films. For wider industrial applications, there is a higher demand for large-scale preparation. One-dimensional and two-dimensional heterostructures can be created in various forms, including transverse 1D/2D heterostructures, longitudinal 1D/2D heterostructures, and hybrid 1D/2D heterostructures. In this section, the preparation of 1D/2D heterojunctions with different structures using PVD will be introduced to provide readers with more specific synthetic reference methods, and finally, the existing problems and possibilities for the development of 1D/2D heterostructures synthesized by PVD will be presented.

**Figure 7:**
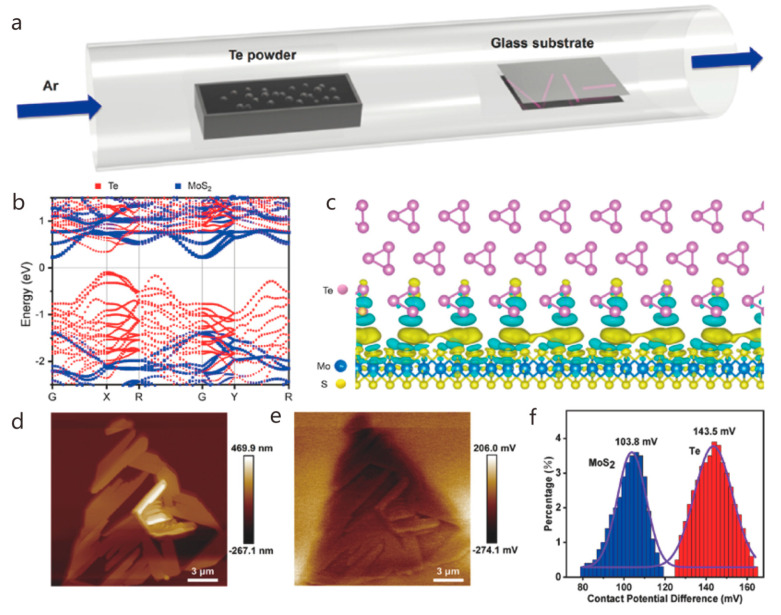
(**a**) Schematic illustration for Te NWs synthesis setup. (**b**) The band structure of the Te–MoS_2_ heterostructure as shown in the figure. (**c**) Charge difference isosurfaces of Te–MoS_2_ heterostructures. (**d**) AFM image of Te–MoS_2_ heterostructure. (**e**) KPFM image of Te–MoS_2_ heterostructure. (**f**) SP histogram of Te and MoS_2_. Reproduced with permission from the authors of [6].

**Figure 8:**
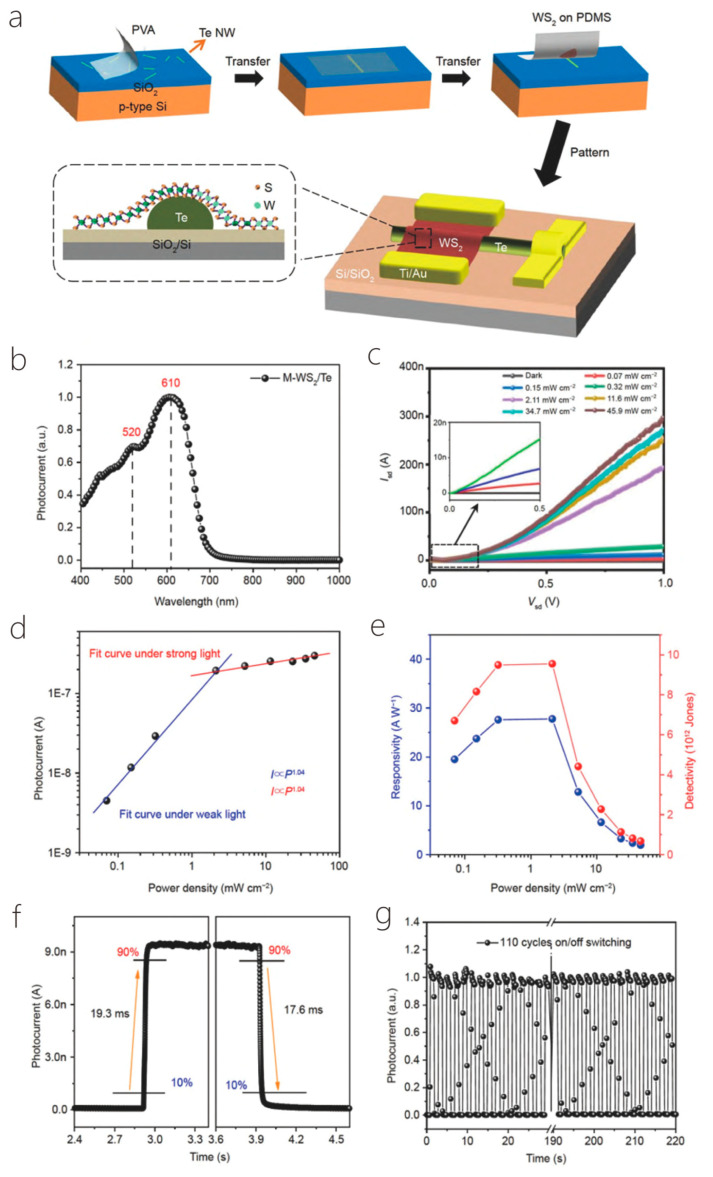
(**a**) Schematics of the fabrication process of the WS_2_/Te device and the local atomic struc-ture. (**b**) The device’s photoresponse spectrum spans 400–1000 nm. (**c**) The device’s I–V curves at varying light intensities. In the presence of a bias voltage of 1 V, the incident light power. The device’s I–V curves at varying light intensities. The incident light power depends on the bias voltage, which is 1 V. (**d**) Photocurrent. (**e**) Responsivity and detectivity. (**f**) Temporal photoresponse under the bias of 1 V. (**g**) Photoswitching characteristics of the WS_2_/Te photodetector in 110 cycles. Reproduced with permission from the authors of [67].

NW field effect tubes exhibit excellent device performance and have drawn much attention because of their unique characteristics, such as high hole mobility, strong optical second harmonic, and stretched term durability when exposed to air, which have promising applications in the area of electronics and photonics. Te’s anisotropic crystal structure produces a strong anisotropic optical response, which displays great polarization imaging in scattering environments. However, the saturable absorption capacity and modulation depth of Te nanostructures need to be renewed and optimized. Transition metal dichalcogenides (TMDs) have broadband saturable absorption and strong photoluminescence properties and may become excellent nonlinear optical structures in combination with Te. Hao et al. synthesized 1D/2D Te-TMD heterostructures by two-step vapor deposition (Figure 7a). In the first place, 2D monolayer TMD nanosheets were prepared on glass substrates using CVD. Secondly, using PVD, the preparation of Te TMD heterostructures requires the epitaxial growth of Te NWs on the surface of single TMD nanosheets. Based on the substrate engineering strategy, the growth orientation of NWs on TMD nanosheets can be effectively controlled. The precursor of the controlled synthesis of Te TMD-vdW heterostructure was selected as Te powder. The grown monolayer of molybdenum disulfide was placed 23 cm downstream of the furnace core, the temperature of the furnace core was heated to 300 °C in 20 min through Ar with a carrier flow rate of 40 sccm, and the growth time was set to 20 min to grow large-scale 1D/2D Te–MoS_2_ vertical heterostructures. The controlled large-scale 1D/2D vertical Te-TMD heterostructures with epitaxial growth were successfully demonstrated, which fills some gaps in the research on the direct epitaxial development of Te-TMD vdW in wide-region heterostructures and expands the research on the controlled preparation of Te-TMD heterostructures. It is also demonstrated that the saturable absorption performance of Te-TMD heterostructures is significantly enhanced due to the Te and TMD (Figure 7b–f) efficient interlayer charge transfer, which contributes new possibilities and methods for the growth of nonlinear optical devices based on 1D/2D Te-TMD vdW heterostructures. The absence of a useful built-in electric field and conductance gain mechanisms in 2D-WS_2_ limits its application in photodetectors [6]. Zhou et al. proposed a heterostructure hybrid photodetector containing 1D-Te and 2D-WS_2_ (Figure 8a), which uses Te to induce in-plane tension within the 2D-WS_2_ and modulates the local WS_2_ electronic structure to produce the type-II band. Not only that, but the vertical heterojunction constructed from 1D-Te and 2D-WS_2_ introduces a high conductance gain, which optimizes the transfer of photogenerated carriers and improves the sensitivity of the prepared photodetectors (Figure 8b–g). NWs were prepared by PVD, and a Te NW was transferred onto a SiO_2_/Si substrate with the assistance of polyvinyl alcohol. This was followed by the dry transfer of multilayered WS_2_ nanosheets obtained using mechanical exfoliation onto the NWs via a three-dimensional positioning platform to obtain hybrid 1D/2D heterostructures made of Te NWs and WS_2_ nanosheets. These overcame the absence of a strong internal electrical field and conductivity gain mechanism of traditional 2D, overcoming the drawbacks of conventional 2D, which lacks an effective built-in electrical field and conductivity gain mechanism, carries on the traits of both materials, and expands on their applications [67]. Kapil Bhorkar et al. investigated the optical and electronic properties of a heterojunction between 1D-Sb_2_Se_3_ and 2D-PtSe_2_. Based on this structure, the PtSe_2_ layer was used as a barrier layer to attenuate the oxidation tendency of Sb_2_Se_3_, while increasing the light absorption in the midinfrared. The substrate was initially ultrasonic cleaning for 10 min using a turbidity remover, ethanol, and deionized water, and then, the Sb_2_Se_3_ target was obtained by high-temperature melting and etched for approximately ten minutes using argon plasma to remove the surface impurities. Afterwards, an amorphous film of Sb_2_Se_3_ was placed on the substrate using RF magnetron sputtering, then, a little coating of Pt was deposited upon the outside of the Sb_2_Se_3_ film using thermal evaporation, and finally, low-temperature solemnization was performed using ultrapure Se pellets to grow the PtSe_2_/Sb_2_Se_3_ heterojunction. Raman spectroscopy results showed that both PtSe_2_ and PtSe_2_/Sb_2_Se_3_ heterojunctions produced indirect band gaps of ~0.30 ± 0.05 eV and ~0.40 ± 0.05 eV. Combined with XPS and UPS data, the energy band structure of the PtSe_2_/Sb_2_Se_3_ heterojunction interface is of type II [24]. Quasi-one- and two-dimensional ZnO nanostructures were prepared by the thermal evaporation method of Chen et al. During the growth process, the deposited ZnO stem nanorods rapidly developed into one-dimensional structures through the vapor solidification mechanism. Driven by the thermodynamics of heteronuclear tendency, a steady stream of reactant vapor may nucleate on the surface of the grown ZnO stem nanorods and further grow to become nanoparticles or nanorods through dendrite growth or epitaxial growth, respectively, resulting in quasi-one-dimensional, granular, rod-like and two-dimensional, rod-like ZnO nanostructures. The specific experimental steps were as follows: ZnO and graphite powders were placed 1:1 into an alumina boat, and then, the alumina boat was moved into a quartz tube that was placed within a tube furnace. The tube furnace was then heated to 1050 °C for 30 min, with a regulated vacuum pressure of 200 Torr and a carrier gas flow rate of 350 sccm. Afterwards, the vacuum was evacuated to 2 × 10^−2^ Torr. The belt of the furnace was naturally cooled to room temperature, and quasi-one-dimensional and quasi-two-dimensional ZnO nanostructures were obtained. Thus, it seems that epitaxial growth, in addition to twinning, has been shown to be a means of self-assembly [68].

The challenges of PVD in synthesizing 1D/2D heterojunctions should also not be overlooked, e.g., the curvature of the 1D structure makes it difficult to form smooth and clean contacts at the 1D/2D interface, which puts higher demands on high-performance device fabrication. In addition to this, the heterojunction interface needs to be further optimized to reduce interfacial defects and inhomogeneities in order to improve its stability. There are also challenges in the extensive 1D material integration on 2D material, the performance of 1D/2D heterojunction materials is heavily reliant on the atomic-level structure and chemical environment of the interface, and precisely controlling the growth conditions and interface quality of the materials is an important challenge. Overall, the synthesis of 1D/2D heterojunction materials using PVD technology has a promising application, and despite some current challenges and limitations, it is expected to achieve a wider range of applications in the future through continuous technological innovation and research.

### 2.3. Hydrothermal Methods

Hydrothermal methods are methods of synthesizing materials in aqueous solutions at high temperatures and pressures. This technique takes advantage of the unique solubility and reactivity of water in a supercritical state, enabling the preparation of materials with high purity and crystallinity, and is widely used to prepare a variety of nanomaterials, crystals, and complex compounds. It has attracted attention for its mild reaction conditions and environmentally friendly properties. Using the hydrothermal method, it is possible to combine 1D nanomaterials and 2D materials to prepare 1D/2D heterojunction structures. Under relatively mild temperature and pressure, it is favorable to the uniform development of materials and interface formation, and the excellent interfacial properties can enhance the performance of electronic and optoelectronic devices. Moreover, by adjusting the reaction parameters, the morphology and size of 1D and 2D materials can be precisely managed, thus achieving the optimal design of heterojunctions. However, precise control of the interfacial quality and contact properties between 1D and 2D materials is still a difficult problem that requires in-depth research and optimization. Moreover, the current yield of the hydrothermal method is relatively low, which is not fit for industrial production on a wide scale, and in this regard, processes to increase the yield need to be created to satisfy the needs of industrial production. In conclusion, the hydrothermal method has significant advantages and broad application prospects in the synthesis of 1D/2D heterojunction structures, and with the continuous progress and optimization of the technology, it will surely bring more innovations and breakthroughs in the development of new materials and new devices.

#### 2.3.1. 1D

The hydrothermal method is able to obtain 1D nanomaterials, such as nanowires, nanorods, and nanotubes, with high crystallinity and purity and with a specific size and shape under mild conditions, which are widely used in the fields of field effect transistors, photodetectors, and photovoltaic devices. The morphology and size of 1D materials can be controlled by modifying the reaction parameters. However, the hydrothermal technique is dependent on the circumstances of the reaction, the process control is difficult, and small process fluctuations may lead to unstable product quality. Further research and the optimization of process parameters are needed.

Yu et al. successfully prepared GdPO_4_:Tb^3+^ nanorods using a hydrothermal process, in which Gd_2_O_3_ and Tb_4_O_7_ were first disintegrated in concentrated HNO_3_, and deionized water was added after heating to remove excess HNO_3_. Then, (NH_4_)_2_HPO_4_ aqueous solution and NaOH aqueous solution were added to the resulting liquid, and the content of the latter was regulated so that the pH value of the solution reached about 13. The suspension was centrifuged after heating to obtain a suspension, and the retained precipitate was dissolved, stirred, and centrifuged to obtain the final sample. Intense green light emission was observed in the GdPO_4_:Tb^3+^ nanorods [69]. Xiao et al. prepared one-dimensional nanostructured Na_2_Ti_3_O_7_ using the hydrothermal reaction of TiO_2_ in NaOH solution. TiO_2_ was dispersed in NaOH solution with continuous stirring for 30 min, and then, the mixture was moved to a 100 mL autoclave with Teflon-lined. After heating at 160 °C for 48 h, the sample was cooled to room temperature, centrifuged to separate the white precipitate, washed several times with deionized water, and dried at a high temperature to obtain a sample with Na_2_Ti_3_O_7_ as the main component with good crystallinity and thermal stability in which the morphology of nanotubes, nanoribbons, and nanorods could be readily modified by introducing an additional amount of sodium hydroxide [25].

Overall, the hydrothermal method has significant advantages in the combination of 1D nanomaterials, but it also faces some challenges. Making the yield and product quality go hand in hand puts higher demands on the reaction device and reaction conditions. Its sensitivity to the reaction conditions requires precise process control, and further study and the optimization of the process parameters are needed to ensure stable product quality.

#### 2.3.2. 2D

The core of the hydrothermal method is to utilize the unique property of water in conditions of high pressure and temperature to promote the dissolution, diffusion, and recrystallization of reactants and, finally, obtain 2D materials with high crystallinity and purity, which are commonly employed in the domains of optoelectronic devices, catalysis, and sensors. Through the manipulation of reaction parameters, such as temperature, time, precursor concentration, and others, the thickness and lateral dimensions of the 2D materials can be precisely controlled to achieve large and uniform monolayer or multilayer materials, which at the same time, puts higher requirements on the process parameters, which need to be further researched and optimized to ensure the stable quality of the products.

**Figure 9:**
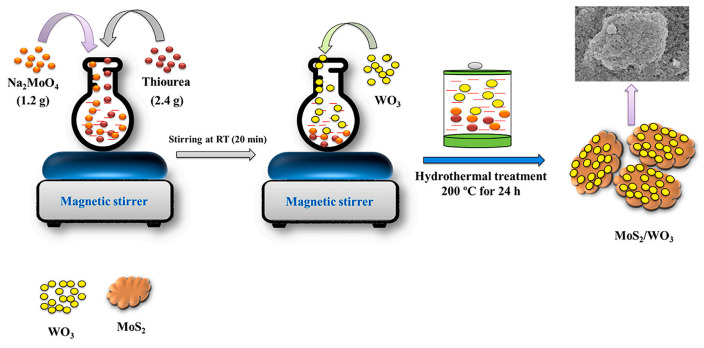
The MoS_2_/WO_3_ composite’s synthesis is depicted schematically. Reproduced with permission from the authors of [70].

Ahmad and Kim prepared glassy carbon electrodes (GCEs) of MoS_2_/WO_3_ composites using the hydrothermal method (Figure 9). First, 2 mg of electrode material (MoS_2_/WO_3_, WO_3_, or MoS_2_) was dispersed in 5 mL of ethanol with 0.1% Nafion as a binder for 1 h using ultrasonication, and then, 8 μL of the dispersed electrode material was dripped onto a cleaned GCE active surface and dried for 6 h. The electrode materials were prepared using a hydrothermal method created by Ahmad and Kim [70]. In Ma et al.’s method, the 3 mm diameter GCE surface was first polished with 0.3 and 0.05 μm Al_2_O_3_ powders and then thoroughly cleaned with ultrasonically deionized water and absolute ethanol before drying with high-purity N_2_. A drop of 6 μL of AuNPs@αNi(OH)_2_/β-rGO nanocomposite was taken on the polished GCE surface and dried at room temperature [26].

However, there are some challenges, and improving the production efficiency of the hydrothermal method to achieve the large-scale production of 2D materials is an issue that still needs to be solved. The high cost of equipment and raw materials, such as high-purity precursors, requires the exploration of low-cost production processes and raw materials, and there is an urgent need to reduce the production cost by optimizing the process and improving the material utilization. Through continuous research and technological improvements, it is expected that these limitations will be overcome to realize the efficient, low-cost, and large-scale production of 2D materials and their extensive use in a variety of sectors.

#### 2.3.3. 1D/2D

With the advantages of mild reaction conditions, controllable morphology and size, and high crystallinity and purity, the hydrothermal method has important application prospects in the preparation of 1D/2D heterojunction structures, which are widely used in the fields of optoelectronic equipment, sensors, and energy conservation. However, the hydrothermal method requires a strong command of the reaction conditions, which is the key to obtaining the desired material structure and properties. There are still several difficulties with large-scale manufacturing using the hydrothermal method, and ensuring the homogeneity and consistency of the products is still a problem that needs to be worked out.

**Figure 10:**
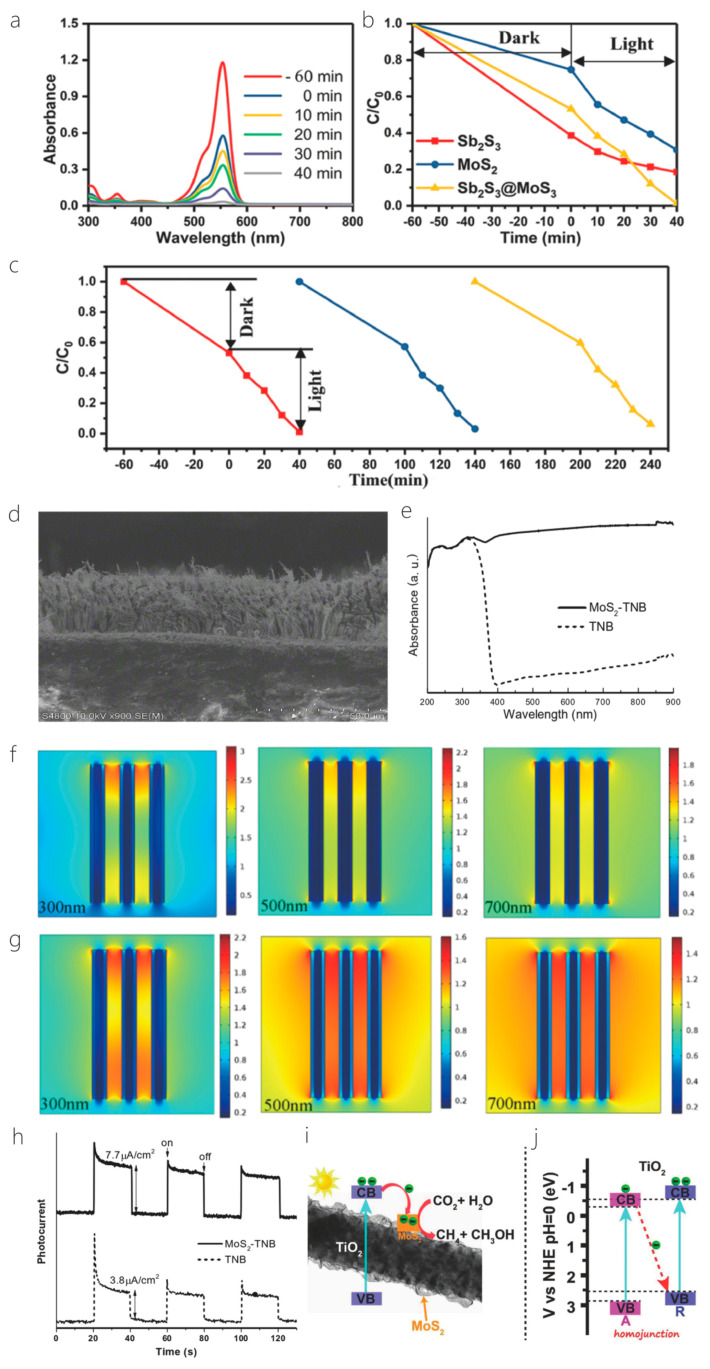
(**a**) UV-Vis absorption spectra of RhB solution at various Sb_2_S_3_@MoS_2_ nanorod reaction times. (**b**) The photocatalytic activities of different products in the same condition. (**c**) The cyclic performance of Sb_2_S_3_@MoS_2_ nanorods under simulated sunlight. Reproduced with permission from the authors of [71]. (**d**) The cross-sectional SEM images represent vertically arranged 1D MoS_2_-TiO_2_ NBs. (**e**) The UV-Vis absorption spectrum can clearly show the effect of absorption enhancement attributes on the deposition of MoS_2_ nanosheets. (**f**,**g**) Finite element method (FEM) simulation effects of the near-field electric field allocation of the 2H MoS_2_-TiO_2_ NB heterostructures excited by different wavelengths in two configurations, and (**h**) PEC photocurrent under 1.5 AM in 0.5 M Na_2_SO_4_ electrolyte. Reproduced with permission from the authors of [27]. (**i**) Diagram showing charge transfer and separation for CO_2_ reduction under UV-visible light irradiation at the TiO_2_/MoS_2_ heterojunction. (**j**) The transmission of charge between rutile homojunction and anatase. Reproduced with permission from the authors of [72].

Xu and Zhao used a two-step hydrothermal method to prepare a new MoS_2_ nanosheet core–shell heterojunction structure covered with Sb_2_S_3_ nanorods, which can extend its photoadsorption range and, thus, have excellent photocatalytic properties. In addition, the unique hybridized 1D/2D core–shell structure consisting of one-dimensional Sb_2_S_3_ nanorods and two-dimensional MoS_2_ nanosheets not only has more reactive active sites, but also, the compact interface between the two materials facilitates a quick charge transfer channel for charge separation. An amount of 1 mmol of SbCl_3_, 2 mmol of Na_2_S-9H_2_O, and 2 mmol of l-cysteine were dispersed and dissolved in 30 mL of deionized water, and the aqueous solution was created and agitated for 3 h. The nanorods were then transferred to 50 mL of Teflon stainless steel autoclave and stored at 180 °C for 14 h. Afterwards, the obtained Sb_2_S_3_ nanorods were centrifuged to separate them, washed several times, and dried at 60 °C overnight. Then, applying the same way as above, Sb_2_S_3_ nanorods (0.589 mmol), Na_2_MoO_4_-2H_2_O (0.248 mmol), and l-cysteine (0.495 mmol) were dispersed in 30 mL of deionized water, and heat-treated in a stainless steel autoclave at 180 °C for 24 h. Finally, the synthesized Sb_2_S_3_@MoS_2_ nanorods were precipitated, washed by centrifugation, and dried at 60 °C for 12 h. According to the results, the Sb_2_S_3_@MoS_2_ nanorods possessed excellent photocatalytic activity and may be a potential material for photocatalytic applications (Figure 10a–c) [71]. Wei et al. investigated a solution-processed synthesis of ultrahigh aspect ratio vertically aligned nanoforests such that the ultrahigh aspect ratio TiO_2_ nanobelts (NBs) were adorned with molybdenum disulfide in order to form 1D–2D heterostructures. Figure 10d–h shows the presentation improvement of vertically aligned 1D MoS_2_-TiO_2_ NB forests. Long anatase TiO_2_ NBs were synthesized using the stir-assisted hydrothermal method. An amount of 1.2 g of anatase TiO_2_ and 80 mL of 10 M NaOH solution were placed in a Teflon-lined stainless steel autoclave set at 200 °C and kept at the temperature for 48 h. Shear or centripetal forces due to agitation preferentially produce higher growth rates at the NB tip, a higher growth rate at the tip of the NBs, and a high stirring rate of 700. The ultralong TiO_2_ NBs were then sintered at 700 °C, and the surface was etched with diluted H_2_SO_4_ solution (0.02 M), followed by MoS_2_ modification, a step that can passivate the trap state of the TiO_2_ surface, thus inducing electron–hole complexes. A continuous molybdenum disulfide outer layer in the form of a core–shell heterostructure was finally obtained. An amount of 30 mg of sodium molybdate and 60 mg of thioacetamide as molybdenum disulfide precursor were mixed with 20 mg of etched TiO_2_ NB powder in 20 mL of DI water, and the mixture was transferred to a PTFE-lined autoclave for the synthesis of 1D MoS_2_-TiO_2_ NBs. After hydrothermal synthesis at 200 °C for 24 h, the samples were thoroughly washed with dewatered water and dried at 80 °C in an oven. Ultrahigh aspect ratio aligned 2D MoS_2_-1D TiO_2_-NBs heterostructured nanoforests arranged with an ultrahigh aspect ratio have been shown to significantly enhance photochemical reactions and have a great future as photocatalysts [27]. Xu et al. prepared 1D/2D TiO_2_/MoS_2_ heterostructured nanofibers using the in situ growth of MoS_2_ nanosheets on TiO_2_ nanofibers via the hydrothermal method, and due to the higher figure of merit for TiO_2_ compared with MoS_2_, the electrons in the MoS_2_ nanofibers are more stable than those in the MoS_2_ nanofibers. The higher figure of merit, electrons were transferred from MoS_2_ to TiO_2_ at the contact interface between MoS_2_ and TiO_2_ to facilitate the separation of carriers under the photoexcitation of MoS_2_ (Figure 10i,j). In addition, the hybridized structure enhances the light trapping ability of TiO_2_. It has a bright future in photocatalysis [72]. Keng Xu et al. prepared NiCo_2_O_4_ nanosheets immobilized on a one-dimensional WO_3_ surface using the chemical deposition method. In a typical synthesis process, the prepared WO_3_ powder is added to 50 mL of deionized water solution, assisted by ultrasound. Then, 1 mmol Ni(NO_3_)_2_·6H_2_O, 2 mmol Co(NO_3_)_2_·6H_2_O, and 30 mmol Co(NH_2_)_2_ is added in sequence, and the combined remedy stirred in a 95 °C water bath for 2 h. Next, the final product is collected, washing is repeated at least 7 times, and the solution is dried at 80 °C. A one-dimensional WO_3_ nanocomposite material consisting of NiCo_2_O_4_ nanosheets and WO_3_ nanofibers is acquired using heat treatment in air at 350 °C for 2 h [28].

### 2.4. 1D/2D Two-Step Van Der Waals Epitaxy

A single material struggles to meet the need for multifunctionality, so multifunctional nanodevices can be realized by reconstructing composite structures with the help of two or more different materials. Nowadays, hybrid integration between 2D materials and semiconductor materials of other dimensions has gained significant progress in terms of performance. One-dimensional and two-dimensional van der Waals hybrid dimensional heterostructures have the advantages of clear atomic interface, high quality, and good compatibility. So far, hybrid 1D/2D heterostructures have received extensive research attention, but their preparation by direct epitaxial growth methods has yet to be thoroughly investigated, while vdW heterostructures constructed from two or more different materials by weak vdW forces provide an effective route to 1D/2D hybrid structures. Compared with mechanical stacking and physical transfer methods, direct gas phase epitaxial growth extends the range of available materials and substrates, enables precise control of material morphology and size, and effectively avoids interfacial contamination. These features offer unique advantages in practical industrial production. Despite the fact that this technique permits for precise control of the grown material, there are still some challenges in practice, such as adjusting the growth conditions, which may lead to instability of the growth process due to the kinetic effects of the growth reaction, contamination of the substrate surface, and other aspects. In addition to this, although van der Waals epitaxy can reduce lattice mismatches and structural defects, there may still be some impurities and defects that affect the properties of the material, especially at the interfaces and growth surfaces.

**Figure 11:**
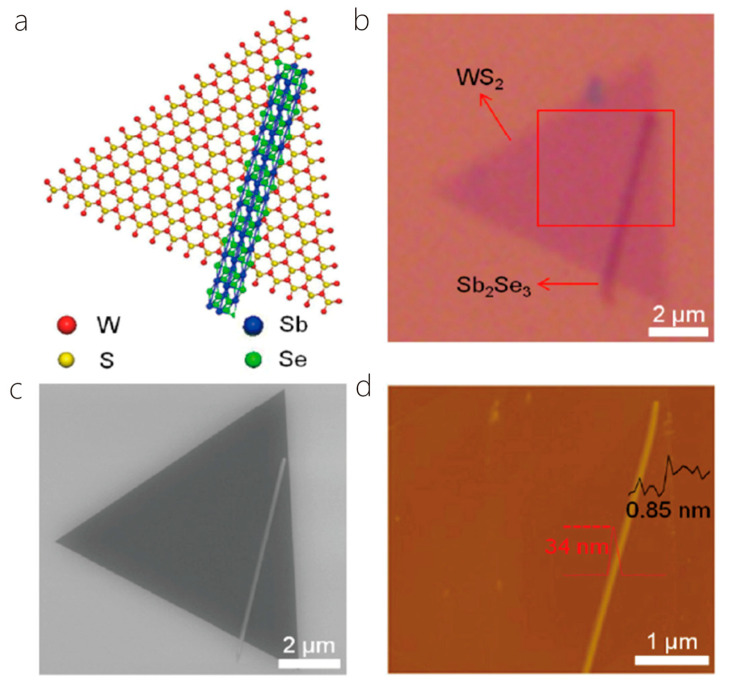
Illustrative diagram and morphological characterizations of the Sb_2_Se_3_/WS_2_ p-n heterostructure. (**a**) Schematic representation of the growth heterostructure. The W atom is represented by a red ball, the S atom by a yellow ball, the Sb atom by a blue ball, and the Se atom by a green ball. (**b**) The optical image shows the Sb_2_Se_3_/WS_2_ p-n heterostructure. (**c**) The SEM image shows the Sb_2_Se_3_/WS_2_ p-n heterostructure. (**d**) AFM image of the corresponding region marked by the red rectangle in (**b**). Reproduced with permission from the authors of [7].

When Sun et al. grew WS_2_ monolayer for the first time, 2 g of tungsten sulfide powder was located in an alumina container in the center of a horizontal quartz tube furnace, and a clean SiO_2_/Si substrate was located at another alumina container downstream of the quartz tube. Prior to heating in a heating furnace, a high-purity Ar (420 sccm) air stream was blown into the tube for 20 min to remove air from the tube. Then, a countercurrent Ar at 100 sccm was maintained, and the furnace center temperature was heated to 1170 °C within 40 min. Since reaching the expected growth temperature, a chemical vapor source was delivered downstream from 100 sccm of forward flowing argon and held at this temperature for 50 s. Then, for the first growth, the furnace was cooled to ambient temperature. For the second growth, three alumina boats were positioned upstream, in the center, and following downstream from the quartz tube, filled up with Se powder, Sb_2_Se_3_ powder, and a SiO_2_/Si substrate with WS_2_ monolayer growth. The vacuum pump dried the air inside the pipe and further rinsed it with a high argon gas flow (810 sccm). After that, the heating furnace was heated to 600 °C and maintained for 2 min. Throughout the growth procedure, the internal pressure was maintained at atmospheric pressure, and the Ar flow rate was commanded at 34 sccm. At the end of growth, the heating furnace was normally cooled to room temperature. Finally, the Sb_2_Se_3_/WS_2_ combined dimensional p-n heterojunction was obtained (Figure 11) [7].

## 3. Photodetectors

Semiconductor heterostructures are fundamental components of highly efficient optoelectronic devices, including solar cells, photodetectors, and light-emitting diodes. The unique physical properties, atomic-thin geometry, and suspension-free surface of two-dimensional van der Waals layered semiconductor materials make them ideal candidates for the formation of heterostructures. These materials easily interact with other layered materials through vdW forces without any constraints or strict lattice matching. However, the limited availability of two-dimensional materials restricts the use of heterostructures in multifunctional devices [73,74,75,76]. Nonetheless, one-dimensional/two-dimensional heterostructures constructed from two distinct materials hold great promise in delivering the portability and wearability that next-generation optoelectronics demand [77,78,79,80,81,82]. However, in the past few years, various 2D material-based photodetectors (PDs) have been manufactured and studied in depth [80]. Additionally, because of the low light absorption and long photocarrier lifetime of molybdenum disulfide crystals, some of the two-dimensional PDs (especially for PDs made of MoS_2_ and other TMD 2D materials) have low photo responsivity and slow response time, which seriously hinders their detection performance [7,29,31]. In the last several years, the application of 1D/2D semiconductor heterostructures has attracted a lot of interest due to their ability to enhance device performance based on layered materials [83,84,85,86,87,88]. Today, we exhibit the achievements of the 1D/2D photodetectors according to the characteristics of one-dimensional materials [89,90,91,92,93].

### 3.1. Metal Oxides

As a typical example of photodetectors, phototransistors with typical photoelectric effects have attracted extensive research interest because their device performance is relatively better than similar devices [30,94,95,96,97]. In the last ten years, a significant number of phototransistors with various device geometries and unusually high optical guide gain or external quantum efficiency have been reported. Most of these devices are typically assembled by integrating some metal oxide semiconductors similar to thin films or nanostructures with other organic or inorganic semiconductor materials. This chapter describes the last decade of UV or visible phototransistors based on metal oxide semiconductors [8,98]. Different kinds of phototransistors, manufactured on different semiconductors, include binary metal oxides (such as zinc oxide, stannous oxide, titanium dioxide), ternary metal oxides (such as InZnO, ZnON), and quaternary metal oxides (such as ZnInSnO, InGaZnO). The operating mechanism of these metal-oxide-based devices is systematically discussed. In addition, some emerging technologies that are widely used to optimize device performance are also discussed [99].

**Figure 12:**
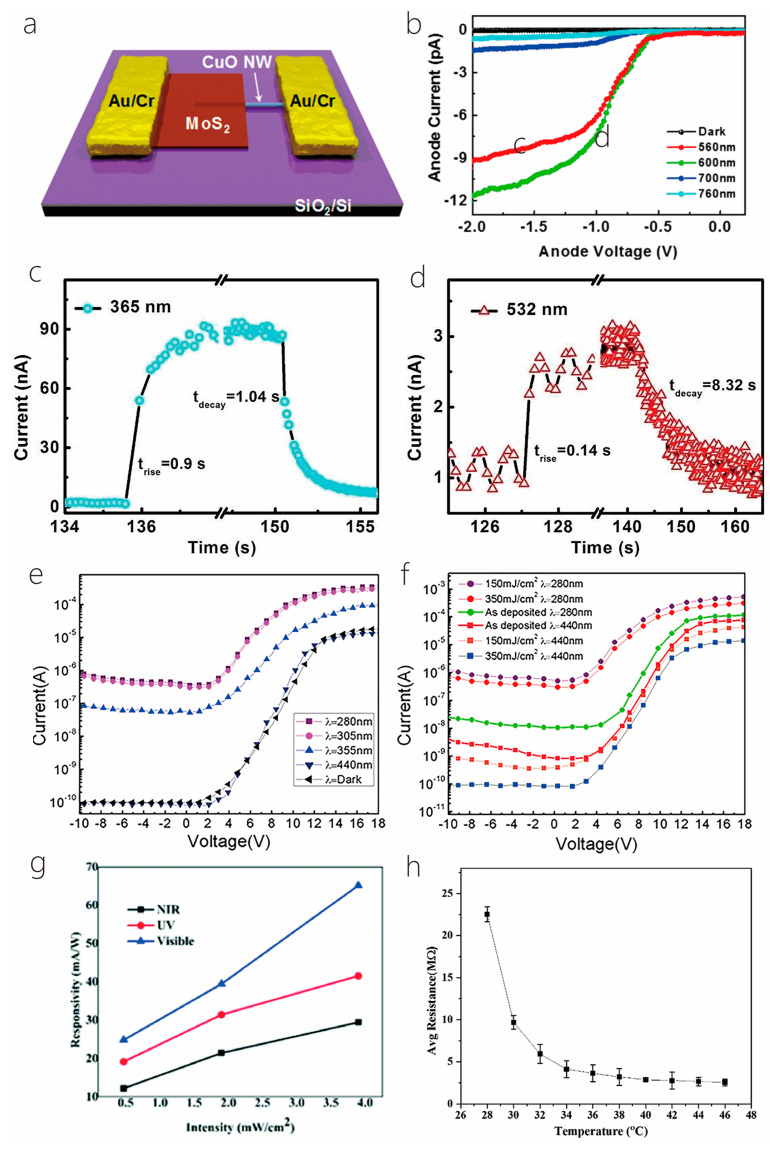
(**a**) Illustrative diagram of MoS_2_/CuO nanosheet-on-1D heterojunction photodiode. (**b**) The photocurrent curves of MoS_2_/CuO nanosheet one-dimensional heterojunction photodiodes under the illumination wavelengths of 560 nm, 600 nm, 700 nm, and 760 nm, respectively, under the incident optical power of 1 mW and under the dark condition. Reproduced with permission from the authors of [8]. (**c**,**d**) Enlarged portions of the transient photoresponse of p-MoS_2_/n-ZnO heterostructure PD under 365 nm and 532 nm light irradiations at a bias of +5 V, respectively. Reproduced with authorization from the authors of [100]. (**e**) Transfer characteristic curves of Device C (V_DS_ = 10 V) exposed to various wavelengths. (**f**) Transfer characteristic curves of Device A without laser annealing with the incident light (V_DS_ = 10 V, k = 280 nm, 440 nm), Device B with laser annealing at 150 mJ/cm^2^, and Device C with laser annealing at 150 mJ/cm^2^. Reproduced with permission from the authors of [81]. (**g**) MoS_2_/V_2_O_5_ responses v/s varying UV, visible, and NIR intensities: exhibits the highest responses. Reproduced with permission from the authors [79]. (**h**) The average resistance versus temperature plot shows the negative temperature coefficient of the resistance (N = 3). Reproduced with permission from the authors of [101].

Huo et al. [30] fabricated a cutting-edge 2D–1D van der Waals heterogeneous structured photo detector by encapsulating CuO/MoS_2_ with an innovative BN material. This state-of-the-art detector possesses an ultrahigh photo responsivity that is 10 times greater than its previous 2D–1D counterpart. The h-BN protection devices showcased exceptional sensitivity, robust optoelectronics, and gate tunability. Tweak the gate and bias voltage, and these gadgets can hit an optical response speed of 2500 AW^−1^ in forward bias mode. They also boast a high detection rate of 6.5 × 10^11^ Jones with a standard rise time of 2.5 ms in reverse bias mode. Additionally, the h-BN packaging acts as an effective shield, safeguarding the hybrid dimensional photo detector from electrical losses caused by O_2_, water, and other gas molecules during FS laser processing or operation. This dramatically enhances the stability and longevity in harsh environments, opening up new possibilities for producing cost-effective and creating long-lasting mixed-dimensional heterostructure photodetectors using femtosecond laser technology for contact engineering. Similarly, Um et al. [8] fabricated a MoS_2_/CuO nanosheet on one-dimensional heterojunction photo detector with an ultrahigh photo responsivity. Now, during wet transfer printing, capillary-assisted attraction induces close contact between the nanosheets and the one-dimensional material, resulting in the constitution of excellent heterojunctions with acute interfaces among molybdenum disulfide nanosheets with copper oxide NW heterostructures (Figure 12a). Figure 12b shows the photocurrent (Ipho) curves in the reverse bias region for light at distinct wavelengths (560, 600, 700, and 760 nm) under incident light and dark conditions under 1 mW power (P_light_) irradiation. The photocurrent in the reverse bias region increases with the increase in the reverse bias, and the wavelength of the light has an important effect on it. The energy band diagram of MoS_2_/CuO NW heterojunction can prove the optical response characteristics in the reverse bias region. When a heterojunction is formed among the n-type molybdenum disulfide sheet with the p-type copper oxide NW, a band alignment mechanism causes band bending in the junction region, resulting in a built-in potential at the interface. Under the influence of light, electron–hole pairs are created in the junction region and are washed away by the internal potential, producing a photocurrent. A rise in the intrinsic potential results in an additional enhancement of the photocurrent upon application of reverse bias.

More recently, Lee et al. [29] fabricated a hybrid one-dimensional zinc oxide NW (n-type) and two-dimensional WSe_2_ nanosheet (p-type) vdW heterojunction device for spectroscopic photo detection and imaging processes. After CYTOP (an amorphous fluoropolymer, CTL-809M from Asahi Glass) packaging, ZnO-WSe_2_ heterojunction diodes exhibited superior performance, with rectification (ON/OFF) ratios exceeding 10^6^ and an ideality factor of 3.4–3.6. Compared with n-type zinc oxide NWs, p-type WSe_2_ has lower conductivity. Nevertheless, the quantity of C-F dipoles within CYTOP results in a greater hole carrier density in WSe_2_. Therefore, it is possible to balance the concentration in the electron–hole carrier of a heterojunction, which improves the performance of the device. ZnO-WSe_2_ heterojunction diodes exhibit a spectral photo response from UV light (400 nm) to NIR (950 nm). In addition to the static photo response, the device exhibits significant optical switching behavior. Finally, a prototype visible imager utilizing this heterojunction diode is demonstrated as a single pixel. Similarly, Zhang et al. [100] proposed an optimal approach for achieving high-performance photodetectors (PDs) through the creation of a mixed-dimensional heterostructure comprised of a 2D-MoS_2_ sheet and a 1D-ZnO nanowire, this integration of 1D ZnO with p-type or n-type molybdenum disulfide results in a mixed-dimensional 2D-MoS_2_/1D ZnO heterostructure PD, which increases the photoresponse range and also upgrades the speed and response time of 2D-MoS_2_ sheets. Figure 12c,d exhibits the amplification of a test cycle used to gauge the photo response speed. The rise time (intensity) quantified the time taken to increase the peak of 10~90% optical flow, with 0.9 s and 0.14 s recorded under light exposure at 365 nm and 532 nm, respectively. The attenuation time (t attenuation) corresponded to the duration taken to lower the peak with a 90~10% photocurrent, clocking in at about 1.04 s and 8.32 s under light irradiation at 365 nm and 532 nm, respectively. Notably, the device’s variance under ultraviolet and green conditions considerably lowered the photoirradiance relative to individual n-ZnO nanowires (which registered at =3.4 s and =2.2 s) and p-molybdenum disulfide sheets (which were recorded at =18.7 s and =112.9 s).

The result of such an experiment shows that under the assistance of 365 nm illumination, the p-MoS_2_/n-ZnO PD exhibits an outstanding photo responsivity, external quantum efficiency, and response time, reaching as high as 24.36 A/W and 8.28 × 10^3^%, respectively, with a speedy response time of merely 0.9 s. Under 532 nm illumination, the photo responsivity, external quantum efficiency, and response time were 0.35 A/W, 80.9%, and 140 ms, correspondingly, which are more effective than those of other reported 2D-MoS_2_ sheet PD performances. All the experiments mentioned above indicate that MoS_2_ with the excellent properties of high mobility and efficient interband absorption could be an ideal material to produce heterojunction phototransistor. Tao et al. also fabricated a photodetector utilizing ZnO [81]. They reported the zinc oxide NRs on integration into InGaZnO_4_ (IGZO) substrates to acquire extremely delicate UV detection in the solar blindness spectrum. In this phototransistor, the photo responsivity (R) was $1.9\times 10^{5}$ A/W, the quantum efficiency (EQE) was $8.7\times 10^{7}$%, the photo sensitivity was $9.5\times 10^{5}$, and the photo detection rate was $8.12\times 10^{16}$ Jones, individually, irradiated with an incident light of 280 nm. Figure 12e shows the transfer characteristic curve of device C (350 mJ/cm^2^ laser annealing) within the range of wavelengths of 280 nm–440 nm. The results show that with the increase in the wavelength of the incident light, the photocurrent decreases with the increase in the visible incident light, and there is no reply to the visible incident light (k = 440 nm). In order to study the performance changes under different annealing processes, Figure 12f shows the transfer curves of incident ultraviolet light (k = 280 nm) and visible light (k = 440 nm). Devices B (150 mJ/cm^2^ laser annealing) and C exhibit a higher optical response at 280 nm incident light, while device A (without laser annealing) had a weaker optical response than B and C.

The results show that zinc oxide NRs/IGZO phototransistors using laser annealing technology are ideal candidates for the development of high-sensitivity solar blind photodetectors. What is more, Sahatiya et al. [79] fabricated a photodetector, while the discrete distribution of one-dimensional vanadium pentoxide nanowires combined with two-dimensional molybdenum disulfide and metal contact molybdenum disulfide allowed the apparatus to absorb light from the 365–780 nm ultraviolet–near-infrared range, where vanadium pentoxide is responsible for UV-visible absorption and MoS_2_ is responsible for visible-NIR absorption. The research also compared the responsivity in the visible, UV, and NIR regions (Figure 12g). The result shows that the prepared photodetector responds more strongly to visible illumination than ultraviolet spectroscopy and NIR, since both V_2_O_5_ and MoS_2_ have absorbance in the visible region. The responsivity of UV, visible, and NIR were found to be 41.5 mA/W, 65.1 mA/W, and 29.4 mA/W, indicating that the device has a more sensitive responsivity in the visible region and has been discovered to be similar to photodetectors made using sophisticated cleanroom technology. 

In addition, the use of local heterojunctions of molybdenum disulfide–vanadium pentoxide to efficiently separate photogenerated carriers can achieve a rapid electron transfer rate, effective charge transfer, and extremely sensitive photo detection. In another study, Veeralingam et al. [101] presented a simple, low-cost, easy-to-fabricate, SnSe_2_-based high-mobility metal insulator semiconductor field effect transistor (MISFET) using a very traditional electromagnetic transistor for an all-paper-based multifunctional photoelectric switch and temperature sensor. SnSe_2_ was synthesized on cellulose paper using the one-step hydrothermal method, and nickel oxide nanofibers were synthesized using electrospinning technology and used as gate mode electricity. MISFETs were prepared using a copper metal strip as source, drain and gate contacts, and nickel monoxide and SnSe_2_ as insulators and semiconductors, respectively. Comprehensive characterization studies exposed the formation of SnSe_2_ nanosheets and nickel monoxide nanofibers. The transfer characteristics of SnSe_2_/NiO with field effect transistors (FETs) of 3.25 μS, a mobility of 20 cm^2^ V^−1^ s^−1^, and an ion–ion ratio of 10^2^ are remarkable considering the simplicity of the cleanroom free manufacturing technology employed. The FET is based on SnSe_2_/NiO as an NIR optical switch and flexible temperature sensor. The NIR photoelectric switch based on flexible SnSe_2_ has a responsivity of 28 mA/cm^2^ and a detection rate of 5.25 × 10^6^ Jones. It must be mentioned that these FET-based temperature sensors have a temperature coefficient of resistance (TCR) value of −4.3 × 10^−3^ C^−1^, which is superior to many commercially available temperature sensors. This is illustrated by Figure 12h. The higher responsivity can be attributed to efficient photogenerated carriers, while the thermally excited electrons of the uniformly grown hexagonal SnSe_2_ nanosheets resulted in a higher TCR.

In recent years, graphene has received a lot of attention due to its high carrier transport mobility, mechanical flexibility, and other different applications. Graphene-based devices exhibit photoelectric, thermoelectric, photothermal, plasma, and other effects, which help to improve the performance of the device; therefore, graphene is also widely used in photonics and photoelectric devices. ZnO NWs are a one-dimensional nanomaterial that have been proposed for use in optoelectronics, photocatalysis, and the charge collection and transport of heterojunctions due to its unique optical, electrical, and piezoelectric properties. ZnO is a wide band gap semiconductor (~3.37 eV) with a high exciton binding energy (~60 meV), which makes it an excellent partner for photocatalysis and the charge collection and transport of heterojunctions in 1D carbon nanotubes and even 2D graphene [99]. Boruah et al. used high-density zinc oxide NWs by growing zinc oxide NWs in air. This approach has allowed us to achieve the uniform growth of NWs over a large substrate area. In addition, the development of large-area graphene has allowed for the fabrication of a photodetector based on a hybrid graphene/ZnO NWs (G/ZnO NWs), which has improved the responsivity and photocurrent gain by approximately 30% and significantly improved response and decay time compared with separately grown zinc oxide NWs devices. In G/ZnO NWs devices, carrier conduction can be carried out through metal electrode → graphene → metal electrode and metal electrode → zinc oxide NWs → zinc oxide thin layer → zinc oxide NWs → metal electrode or metal electrode → zinc oxide NWs → metal. The photo-generated free carriers have a higher probability of conduction through metal electrode–graphene metal electrode and follow a shorter electrical path in the device. In addition, the transfer mobility of photogenerated free-carrier-enhanced graphene from zinc oxide also reduces the recombination rate of photogenerated free carriers due to the high carrier transport mobility of graphene, so it may lead to the enhanced photoresponse of G/ZnO NWs devices [102]. At the interface of the heterojunction-based device, the graphene/TiO_2_ device, electrons are transferred from TiO_2_ to graphene due to the difference in energy levels of the two materials. Therefore, heterojunction is formed at the interface, which hinders the charge recombination of photoexcited electron–hole pairs. This result has prompted many researchers to further investigate ultraviolet photodetectors based on graphene–titanium dioxide nanostructures.

Noothongkaew et al. used titanium foil anodizing technology to prepare pure titanium dioxide and graphene/titanium dioxide nanotube arrays on a transparent glass substrate. UV detector devices were prepared by pure TiO_2_ NT and graphene/TiO_2_ NT devices. It was found that compared with pure titanium dioxide devices, the UV photodetectors of graphene/titanium dioxide devices showed good device performance in terms of high responsiveness, photoconductive gain, and specific detection rate.

The time response of the nanodevices showed that graphene/TiO_2_ NT devices had good stability and repeatability, and the photocurrent was calculated as I_UV-light_ and I_dark_, where both I_UV-light_ and I_dark_ were measured at a bias voltage of 5 V. For pure TiO_2_ NT devices, UV light turns on and off for 2 min per cycle, while for graphene/TiO_2_ NT devices, UV light turns on and off for 1 min per cycle. The average photocurrent of pure titanium dioxide nanodevices is 1.0 µA, while the photocurrent of graphene/titanium dioxide nanodevices is higher, at 11.0 µA. It can be observed that the photocurrent of graphene/titanium dioxide nanodevices is 10 times higher than that of pure titanium dioxide nanodevices. Small changes in the photocurrent of these two devices may be due to the heat generated by the UV illumination as well as the bias voltage. With light repeatedly turned on and off, the nanodevices can reversibly switch between low and high conductances. By comparing the current saturation curve of UV on and off times in titanium dioxide NT and graphene/TiO_2_ NT devices with time, they found that the photocurrent of graphene/TiO_2_ NT devices was significantly higher than that of pure TiO_2_ nanodevices, and the response time was faster in the ultraviolet region. One reason for the higher photocurrent of graphene/TiO_2_ devices may be the higher carrier mobility resulting from graphene coupling and less recombination of photogenerated electrons (e^−^) and holes (h^+^). When the graphene/titanium dioxide device is exposed to ultraviolet light, the e^−^ in titanium dioxide excites from VB to CB, then leaves a positive charge (h^+^) in VB and a negative charge in CB. In the absence of graphene, some photogenerated e^−^ recombine with h+, resulting in lower photocurrent efficiency. In graphene/TiO_2_ devices, e^−^ flows into graphene rapidly due to the strong interaction force between graphene and titanium dioxide and the large electronic storage capacity of graphene. As a result, the light-generated e^−^ quickly migrates to the graphene, while h^+^ remains in the titanium dioxide. Separation of e^−^ and h^+^ at the interface reduces e^−^ and h^+^ recombination. Therefore, compared with pure TiO_2_ devices, it enhances the injection and transportation of charge carriers, as well as a higher photocurrent. The results show that the heterojunction of graphene/TiO_2_ NT devices has a good application prospect for UV photodetectors and is worthy of further study [103].

The photogating effect is a mechanism used by phototransistors to detect sub-band gap rays. The ability of light to change the electronic structure of a material is explained by the fact that when a material is exposed to light, photons can interact with electrons in the material, creating excited electrons and holes. These excited electrons and holes can diffuse into the material, changing its electrical properties. The photochemical effect is produced by the photoinduced capture of carriers. The photochemical effect is often associated with the presence of impurities in the material, called trap states. These trap states can trap photo-generated excited electrons and holes and change the potential energy at the semiconductor/dielectric interface. These changes in charge distribution can contribute an extra gated field, resulting in a change in charge transmission behavior, which is a change in threshold voltage (V_Th_). These trapped charges distinguish the leakage current in dark and light irradiation. Since the optical effect is generated by energy states within the band gap, the optical effect can be used to enhance the optical response [104]. Wang et al. proposed a new high-performance photodetector based on the van der Waals ZrS_3_/MoS_2_ heterostructure to overcome the limitations of low responsivity and response speed present in ZrS_3_ photodetectors. The strong light–matter interaction, combined with the photoeffect, contributes to excellent performance. Electron–hole pairs are generated in the heterostructure under light, and the surface potential is reduced under positive V_DS_, which is conducive to the transfer of electrons from molybdenum disulfide to ZrS_3_, and the hole transports in the opposite direction. Due to the trap present in the heterogeneous interface and molybdenum disulfide, some of the holes will be trapped and produce a photochemical electric field. In principle, the spatial separation of photocarriers may inhibit their recombination, allowing photogenerated electrons to circulate in the external circuit and resulting in a higher photoconductive gain. Due to the charge capture-assisted photogating effect, the rapid spatial separation of electron–hole pairs produced by interlayer carrier transitions and light, the device exhibits superior photoresponse characteristics, with a high responsivity of up to 212 A/W from ultraviolet to visible spectrum, an extraordinary external quantum efficiency of 8.5 × 10^4^%, and a rapid rise/decay time of 0.19/0.38 ms. The photodetector not only integrates the excellent performance of ZrS_3_ and single-layer molybdenum disulfide but also further enhances the benefits through interlayer coupling, demonstrating the strong potential of ZrS_3_-based devices for high-performance, ultrafast, and polarization-sensitive photo detection [105].

Energy transfer plays a crucial and potentially impactful role in heterojunctions, significantly contributing to the performance enhancement and application expansion of photodetectors. This process occurs due to differences in energy levels or interactions between various materials, facilitating the transfer of energy from one material to another. In their research on the synergistic improvement of broadband optical response in MoSe_2_ nanoflakes/ZnO nanorod heterojunctions with plasma-enhanced Au nanoislands, Subhajit Jana et al. utilized this energy transfer mechanism for theoretical analysis. They presented a semitransparent hybrid-dimensional photoelectric detector made from several layers of solution-processed MoSe_2_ nanoflakes combined with vertically aligned ZnO NRs enhanced by plasma treatment. During experiments, they observed that photoluminescence (PL) quenching occurred alongside an increase in UV PL support energy transferred from ZnO NRs to Au nanoislands. The strategically positioned Au nanoislands greatly enhanced the interaction between light, MoSe_2_ nanoflakes, and ZnO NRs within the localized surface plasmon resonance (LSPR) range without compromising absorption at the band edge or causing device inversion. In photodetector design, carefully combining different heterojunction materials and their band structures can facilitate light absorption across a broad spectral range. Moreover, the energy transfer mechanism promotes efficient charge carrier transport among distinct materials, thereby enhancing overall light absorption efficiency. Additionally, variations in band structure and electron affinity among different materials allow for the effective separation of electrons and holes into separate components within heterojunctions; this separation minimizes the recombination losses of charge carriers while boosting the photoelectric conversion efficiency in detectors [106].

To sum up, this part reviews a variety of strategies and examples used by researchers to enhance the metal oxide semiconductor-based system’s photoelectric performance PD, which meets the different requirements of excellent spectral selectivity, high responsivity, large detection rate, fast speed, excellent stability, etc. Thus far, there are still a large number of technical problems in improving the photoelectric performance of PD based on metal oxide semiconductors. Given that the majority of proposed tactics only yield performance improvements in one aspect without systematic consideration, there is an urgent need to simultaneously explore novel sensitive semiconductor nanostructures and achieve efficient device structure engineering [80].

### 3.2. TMDs

Two-dimensional (2D) layered materials such as transition metal disulfides (TMD) (molybdenum tellurium, tungsten selenium, etc.) owing to the quantum resonance effect and a strong interlayer coupling effect. The unique thickness-dependent and strain-adjustable physical properties have garnered a lot of interest [92,93]. In the last several years, in addition to the discovery of graphene, other novel single-element two-dimensional layered materials with a changeable band gap and high theoretical carrier fluidity have been presented, such as black phosphorus (BP), arsenic (As), bismuth (Bi), tellurium (Te), and antimony (Sb), showing an adjustable band gap, high theoretical carrier fluidity, atomic surface, strong spin–orbital torque, high light absorption efficiency, and experimental exploration as hopeful competitors for field effect transistor (FET), spintronic, and photodetector (PD) applications.

#### 3.2.1. Te

In the midst of the abovementioned materials, tellurium is a quasi-two-dimensional semiconductor with a theoretical band gap and a triangular crystal structure. Uniquely, it has many one-dimensional chains of Te atomic spirals stacked together by weak van der Waals (vdW) forces along the c-axis, resulting in a mixture of wire and nanosheet forming types. In 12 experiments, Te exhibited outstanding qualities, such as recording high pore mobility (700 cm^2^ V^−1^ S^−1^), extraordinary air permanence (over two months), primitive anisotropic structure (anisotropy ratio 1.43), and wideband absorption spectrum (520 nm to 3.39 μm), making it a possible candidate for future electronics and photoelectrons.

**Figure 13:**
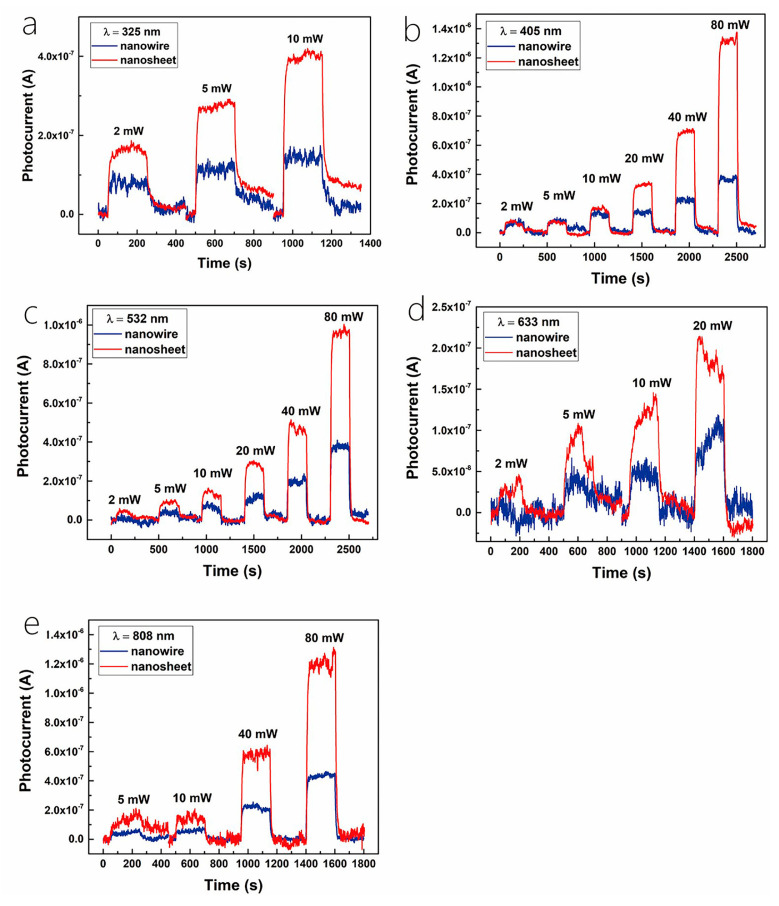
Photocurrent responses of a single GaTe nanowire PD (blue) and nanosheet PD (red) under illumination at different excitation intensities at the wavelengths of (**a**) 325 nm, (**b**) 405 nm, (**c**) 532 nm, (**d**) 633 nm, and (**e**) 808 nm (to explain the reference to color in this graphic legend, see the web version of this article). Reproduced with permission from the authors of [107].

Tien et al. [107] fabricated a photodetector utilizing two diverse GaTe nanostructures using focused ion beams (FIBs). Figure 13 demonstrates the photocurrent (IP) response of an individual GaTe nanowire and nanosheet PD upon excitation at 325 nm, 405 nm, 532 nm, 633 nm, and 808 nm, respectively. The background dark current was subtracted to exhibit the photocurrent response. The outcomes reveal that the spectral range of illumination for both GaTe nanowires and nanosheets varies widely from ultraviolet to near-infrared regions, with nanosheet PD devices displaying a greater photocurrent in contrast to nanowire PD devices. It is rational to notice a significant photocurrent response in GaTe nanosheets, taking into account their broad effective area. 

**Figure 14:**
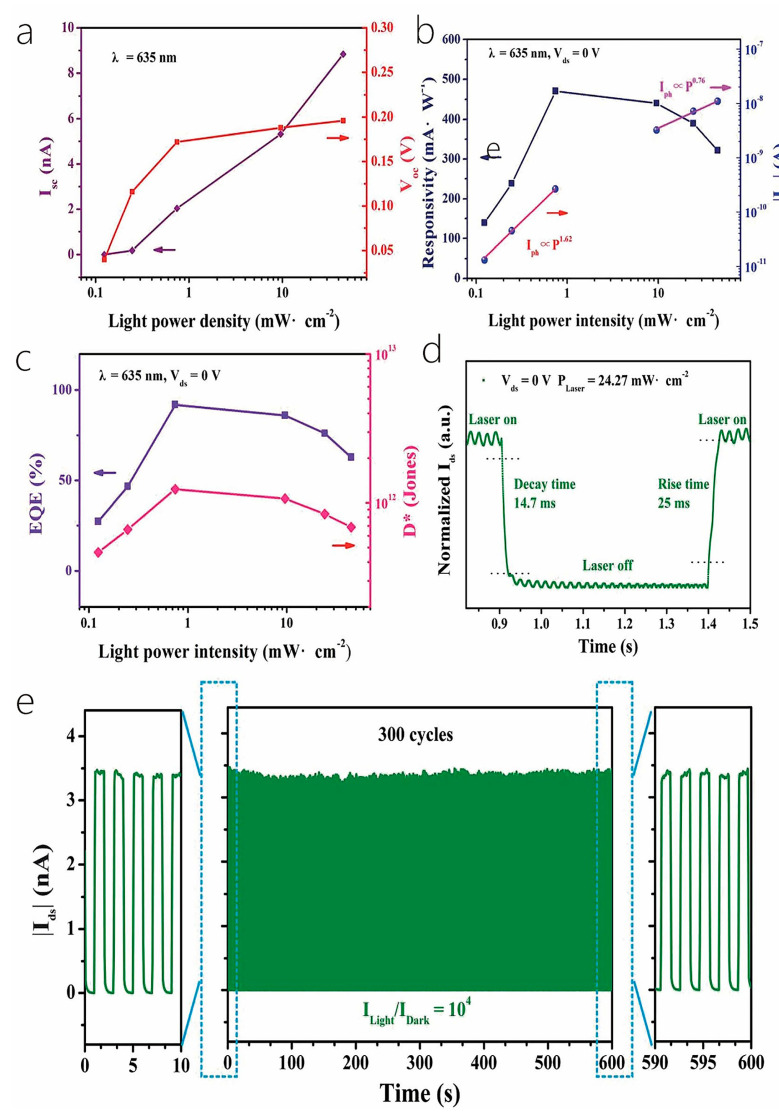
Self-supplied electro-optic response characteristics of heterostructure devices under 635 nm illumination. (**a**) The Voc and Isc of the equipment are related to the illumination power intensity. (**b**) At the 0 V bias, the photocurrent and R_λ_ change with the incident intensity. (**c**) EQE and D* as a function of the optical power intensity. (**d**) Rise and decay time of the device under 24.27 mW cm^−2^. (**e**) Photo response of a photodetector with 300 cycles. Reproduced with permission from the authors of [93].

The result shows that GaTe nanowire PD and GaTe nanosheet PD have high sensitivity, high responsivity, high normalization gain, and high detection rate in the wide spectral range from ultraviolet to NIR. In addition, GaTe nanowire PD has greater photo detection performance matched to GaTe nanosheet PD, which may be owing to the lower carrier scattering probability and higher carrier mobility of one-dimensional GaTe nanowire PD. The photo detection mechanism of one-dimensional and two-dimensional GaTe PD was investigated and considered. The remarked photo detection performance is significantly higher than that of one-dimensional or two-dimensional PD synthesized by the CVD method. Similarly, Han et al. [93] proposed a mixed-dimensional, vertical heterostructure by transferring mechanically stripped two-dimensional WS_2_ nanosheets onto epitaxial growth one-dimensional tellurium (Te) microfilament. On the basis of theoretical type II band alignment, the device displays a photoelectric volume effect and acts as an outstanding self-powered photodetector with a maximum open circuit voltage (Voc) of about 0.2 V. The built-in current causes the open circuit voltage (Voc) of the device, because electrons accumulate on the p-Te microfilament side and photogenerated holes accumulate on the n-WS_2_ side. Figure 14 shows the self-supplied electro-optic response characteristics of heterostructure devices under 635 nm illumination. With the improvement of optical power intensity, built-in electric welding is enhanced, bringing out an increase in the nonlinearity of the device Voc and a linear enhancement of Isc. At 0.74 mW cm^2^, the maximum photoresponsivity of the automatic force of the heterogeneous structure is as high as 471 mA W^−1^. Due to the complicated transfer and recombination process of optical carriers, the index (a) values acquired by analyzing the measurement data are 1.62 (under low light) and 0.76 (under strong light), respectively.

Furthermore, the dark current of the SPPD is highly suppressed to the subpar level owing to the large built-in electric field in the transverse, contributing to an I_light_/I_dark_ ratio of 10^4^, a rise time of 25 ms, and a decay time of 14.7 ms. With a negative bias of −2 V, these properties can be further enhanced.

**Figure 15:**
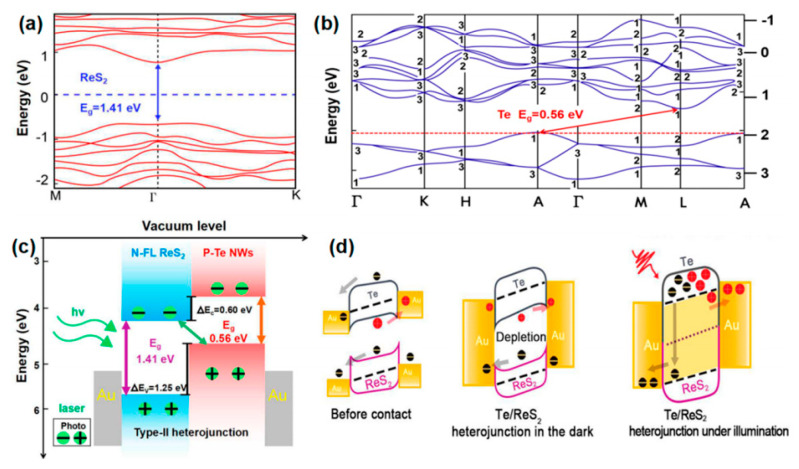
(**a**) Electron band structure of ReS_2_. (**b**) Electron band structure of Te. (**c**) Band alignment of the Te/ReS_2_ heterojunction. (**d**) Energy band diagram and carrier transport of the Te and ReS_2_ before contact, after contact, and under light irradiation. The Fermi energy is set as zero energy. Reproduced with permission from the authors of [31].

Jiang et al. [31] proposed a mixed-dimensional van der Waals heterojunction photo detector containing high-efficiency one-dimensional p-type tellurium (Te) and 2Dn-type ReS_2_ and deposited Te nanowires on ReS_2_ nanosheets using the dry transfer method. Since type II p-n heterojunctions are formed at the interface of ReS_2_ and Te, the effectiveness of the injection and separation of photoexcited electron–hole pairs can be improved. The density functional theory is shown in Figure 15a,b, from which it can be seen that the direct band gap of ReS_2_ is 1.41 eV, and the band gap dimension of Te is 0.56. As shown in Figure 15c, the maximum conduction value (CBM) and minimum valence band value (VBM) of Te are both higher than those of ReS_2_. Thus, the Te/ReS_2_ heterojunction has a type II band arrangement. Figure 15d shows the optical carrier transmission of Te/ReS_2_ heterojunction under light and dark conditions. Under dark conditions, Au, ReS_2_, and Te have different Fermi energy formations before they come into contact with each other. The contact of ReS_2_ and Te with Au forms a low Schottky barrier at the interface. During Te/ReS_2_ heterojunction formation, charge transfer and electron–hole recombination occur at the interface, and this charge transfer behavior reduces/raises the Fermi level of ReS_2_ (Te), leading to the formation of depletion regions and introducing an internal field to ReS_2_. At the Au/ReS_2_ (Au/Te) interface, depletion region formation is accompanied by a reduction in the Schottky barrier, which significantly inhibits the dark current in heterojunction bilayers. The use of 58 illumination results in the generation of electron–hole pairs in Te NWs. Thus, electrons are transferred to ReS_2_NFs, and photoexcited holes in ReS_2_NFs are transferred to Te NWs. Charge separation is beneficial for reducing electron and hole recombination, thus enhancing the photogenerated current. This phenomenon increases the Fermi level of ReS_2_ NFs and decreases the Fermi level of Te NWs. The accumulated electrons (holes) in ReS_2_ NFs (Te NWs) can be transferred via an applied electric field of V_DS_ driven by two channels and immediately captured by the electrodes, resulting in a high-photo current. Comparing the original Te and ReS_2_ photodetectors, the heterojunction device is sensitive to visible photo sensitivity (632 nm) and has an ultrafast optical response (5 ms), a high responsivity (180 A/W), and a specific detection rate (10^9^). The responsivity and the response speed are obviously better. These results show that Te/ReS_2_ hybrid heterojunctions and high-performance optoelectronic devices and sensors have great development space in the future.

#### 3.2.2. Se

**Figure 16:**
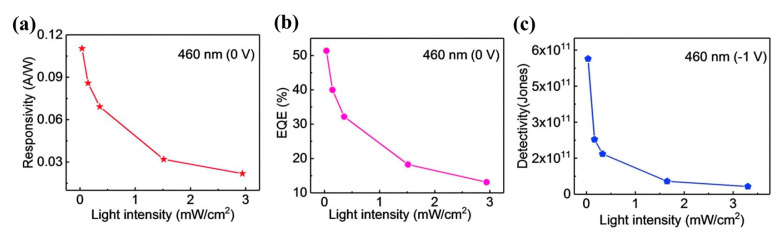
The performance of the Se/InSe photodetector. (**a**) The responsivity of this photodetector at zero bias voltage. (**b**) EQE at zero bias voltage. (**c**) The detectivity at −1 V. Reproduced with permission from the authors of [112].

Se, as a p-type semiconductor, has a band gap of 1.67 eV and, thus, exhibits many outstanding properties, such as high photoconductivity (8 × 10^4^ S cm^−1^), high conductivity (≈0.85 S cm^−1^), and a relatively low melting point (≈490 K) [8,96]. Nonetheless, as shown in our past work, Se has a great possibility for building photodetectors from the UV to the visible region. Due to its chain-like molecular structure, Se spontaneously takes on 1D forms, for instance, 1D nanotubes. In particular, one common example of a one-dimensional elemental semiconductor material is trigonal selenium (t-Se), which has many interesting physical properties, such as high light conductivity [108,109], high voltage [110], and nonlinear optical response [96]. It has a highly asymmetrical crystal structure in which selenium atoms are covalently bonded to form helical chains along the [111] crystal direction, while adjacent chains are stacked together by vdW forces to form a hexagonal structure. In particular, the suitable 1.7 eV bandgap and low dark current make t-Se widely used in UV-to-visible photo detection [96]. Shang et al. [112] fabricated a novel mixed-dimensional vdW heterojunction composed of one-dimensional p-type selenium nanotubes and two-dimensional flexible n-type InSe nanosheets, using a facile method, which has excellent photovoltaic characteristics. As illustrated in Figure 16, the values of external quantum efficiency (EQE) and responsivity (R_λ_) show a nonlinear decline as the illumination power (P) increases. At zero bias voltage and under a photo intensity of P = 40 μW cm^−2^, these values can potentially rise up to 110 mA W^−1^ and 51%. However, as the light power density rises from 0.04 to 3 mW cm^−2^, the values gradually decline from 110 to 28 mA W^−1^. It is important to note that, since our monochromator has a 68 × 68 mm slit and a reciprocal dispersion at the slit of 2.3 nm mm^−1^, the optical power range used in the experiments can be changed to an irradiance range of 0.03 to 3 mW cm^−2^. These values correspond with the common sunlight irradiance that is experienced on the earth’s surface.

The result shows that owing to the great performance of the hybrid p-n junction, the mixed dimensional van der Waals heterojunction exhibits a high on/off ratio at a relatively weak light intensity of 3 mW cm^−2^ [112]. A wideband self-powered photodetector from ultraviolet light to the visible region is realized. In the absence of an external power source, the device can have a maximum responsivity of 110 mA W^−1^. This value is about the same for the original Se device at 5 V and the InSe device at 0.1 V. In addition, even at bias voltages, the response speed is ten times quicker than the speed of a single Se or InSe gadget. This move explores a new direction for the further invention of high-performance, low-cost, and efficient photodetectors using hybrid dimensional vdW heterostructures. In another study, Lee et al. [29] fabricated a novel photo detection and imaging diode, which consists of a one-dimensional zinc oxide nanowire (n-type) and a two-dimensional WSe_2_ nanosheet (p-type) vdW heterojunction. By passivating the device with amorphous fluoropolymer, the ZnO-WSe_2_ diode exhibits remarkable performance, including a rectified (ON/OFF) ratio exceeding 10^6^ and an ideal coefficient of 3.4–3.6 owing to the carbon–fluorine (C-F) dipole effect. Moreover, the heterostructure device demonstrates a spectral photo response from ultraviolet (400 nm) to near-infrared (950 nm). Furthermore, using a ZnO-WSe_2_ heterojunction diode as an imaging pixel, a prototype visible imager has been developed. This is the first successful application of optoelectronic equipment based on 1D–2D hybrid vdW heterojunctions. The use of one-dimensional ZnO-2DWSe_2_ heterojunctions opens up promising avenues for the development of mixed-dimensional vdW heterostructures for electronic/optoelectronic applications.

Chen et al. [94] also propose a simple method to balance the Fermi level of bipolar two-dimensional materials with gate bi-as to balance the interface features of MD-vdW heterojunction photo detectors. The GaAs-WSe_2_ MD vdW heterojunction automatic force photodetector with different metal contact points verified the effectiveness of the gate-adjustable interface characteristics. Under gate bias conditions, the responsivity of the GaAs-WSe_2_ heterojunction photodetector with a Au/Cr electrode increased from 122.55 mA/W to 510.98 mA/W, which was better than the most advanced GaAs-based self-powered photodetector. This work offers a straightforward and practical method for the preparation of a high responsivity, automatic force heterojunction photo detector using the gate-adjustable interface characteristics.

## 4. Sensors

As society and technology flourish, the fields of smart robots, artificial skin, flexible electronics, and healthcare are at first glance and show the expected boom at an alarming rate [113,114,115,116,117,118]. At the same time, as an important part of intelligent detection equipment, sensors can bloom quickly to meet the requirements of high sensitivity, wide detection range, and stable response sensing ability [119]. Sensors convert external stimuli, including biological, chemical, and physical changes, into changes in electrical signals. According to the respective operating mechanism of the sensor, the sensor can be divided into the following categories: piezoresistive [120,121,122], piezoelectric [123,124,125], friction [126], and capacitive [127,128]. Due to the feature of the simple structure, low cost, and conventional signal acquisitions, piezoresistive sensors have already attracted widespread attention, which could be further used in the field of wearable detection devices [129,130]. It works by converting external stimuli into visual electrical signals, while changes in electrical resistance are created by the microscopic deformations of its own structure [129,131]. From the perspective of sensor function, sensors can also be divided into strain sensors [132,133,134], stress sensors [135,136], humidity sensors [137], gas sensors [9,32,34,138], temperature sensors [101], and so on [10,139]. From an overall perspective, the main challenge at hand is how to create the perfect sensors that are compatible with a large detection range, ultralow reaction time, ultralow detection limitation, excellent linearity, and cyclic stability [9,32,34,101,138,140,141]. In this part, we would like to summarize some novel and superior sensors, including gas sensors and pressure/strain sensors.

Considering multiple factors, such as sensor performance, stability, reliability, and cost effectiveness, the selection of the sensing material is very important. One-dimensional materials have a large aspect ratio of the structure, which can effectively interact with the target molecules or substances to enhance the detection sensitivity of the sensor to the target substances. Its geometry and size can promote the rapid transfer of charge or energy, and by adjusting the parameters, such as size, shape, and surface properties, it is also possible to regulate and enhance the sensor’s performance. The 1D heterostructures composed of these structures have the property of recognizing the heterogeneous interfacial effect that accelerates the charge conversion, which has garnered a lot of interest in the field of flexible electronics [138]. The above advantages make 1D structures important in specific sensor applications. The ultrathin thickness of 2D semiconductors provides a great surface area and oceans of reaction sites to facilitate the charge transfer between gas molecules and sensing materials. Two-dimensional materials have high electron mobility, which can effectively improve the sensor and response speed and detection sensitivity; additionally, its excellent mechanical flexibility can be adapted to complex surfaces, applicable to a wider range of scenarios. These properties make 2D structures ideal for sensor applications. Although 1D and 2D structures have demonstrated many unique advantages in sensor applications, they also face a number of shortcomings and limitations, including the stability of the material, the complexity of the manufacturing process, structural defects, and so on. In practical applications, these factors need to be considered comprehensively to continuously improve the material preparation technology and optimize the device structure design to improve the presentation of the sensors. To solve the current dilemma, there is an urgent need for emerging heterostructures to open up fresh channels for basic research and application technologies. Researchers have found that 2D materials combined with 1D or other dimensional materials to form mixed-dimensional heterostructures can overcome the limitations of a single material to a certain extent, and this integrated structure can exhibit a range of advantages in one material. For example, 1D–2D heterostructures have received more and more research attention, and they are now breaking through to become popular hybrid dimensional heterostructures [9].

### 4.1. Gas Sensors

**Figure 17:**
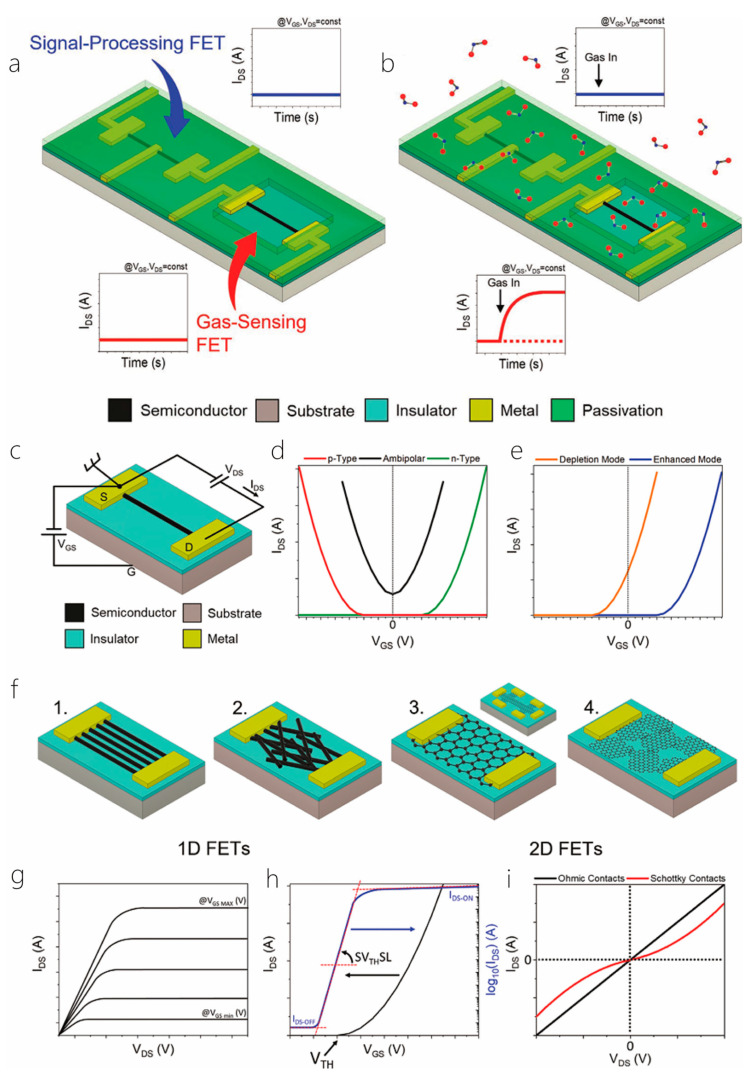
Schematic of a 1D FET for gas sensing and signal processing integrated side by side on the same chip. The change in environmental chemistry from (**a**) to (**b**) does not influence the signal processing FET, which is topped with a passivating layer that prevents the 1D material from interacting with the environment, so the current flowing through the FET, the I_DS_, depends only on the bias conditions (compare the blue traces in (**a**,**b**)). In contrast, in a gas-sensing FET, the 1D material is in touch with the environment directly, so that the I_DS_ will change when the chemical composition of the environment changes (for example, the presence of NO_2_ molecules) under a given two-base condition (compare the red traces in (**a**,**b**)). The same principle is suitable for 2D FETs. One-dimensional and two-dimensional FET gas sensor architecture and electrical behavior. (**c**) The figure shows the typical architecture and bias mode of a one dimensional FET gas sensor based on a single NW. (**d**) Transfer characteristics of P-type, N-type, and bipolar FETs. (**e**) Transfer-characteristics of depleted and enhanced N-type FETs. (**f**) Architecture description of 1D and 2D FET gas sensors: 1. 1D-FET based on aligned NW networks; 2. 1D-FET based on NW random network; 3. 2D-FE based on a single sheet. The inset shows a 2D-FET based on a single-layer sheet, characterized by Hall rod geometry; 4. 2D-FET based on a slice irregular network. (**g**) Output characteristics of an n-type FET. (**h**) The black solid line represents the linear transfer characteristic, and the blue solid line represents the logarithmic transfer characteristic of the N-type FET. (**i**) The output characteristics are close to V_DS_ = 0, highlighting the electrical behavior of FETs with Ohmic and Schottky contacts. Reproduced with permission from the authors of [143].

Since they were first proposed in 1962 [142], gas sensors using oxide semiconductors have undergone extensive research and development and are now an important device for detecting leaks of several flammable gases, such as town gases and some toxic gases like NO_2_ [9]. In the past few years, many research efforts have concentrated on the detection of gases in low concentrations, such as odor components. To meet this low level of gas, the sensor should be highly upgraded with regard to sensitivity, selectivity, and stability. What is more, the use of semiconductor 1D and 2D materials as conduits for FETs, instead of in two end devices (such as resistors, capacitors, diodes), has given rise to fresh and various gas sensor structures with significant benefits in respect to sensing performance. The current passing through the FET sensor is modulated through the gate terminals, which enables the electrical adjustment of sensitivity to detect both tiny and great concentrations of target gases. In addition, distinct electrical parameters can be used to track how gas molecules are adsorbing on nanomaterials, allowing multiparameter sensing, gathering information on sensing mechanisms, and improving selectivity to the analyte of interest. These advantages have greatly promoted the research activities of 1D and 2D FET gas sensors, i.e., the integration of gas sensing and signal processing as a whole on the same chip (Figure 17) [143]. 

This section unfolds the research results of gas sensors by refining the material preparation method, optimizing the material assembly structure and fabrication process. The following section focuses on the synthesis strategy to directly integrate the sensing materials onto the sensor, the treatment to modulate the surface properties of 1D/2D structures by surface modification, and the oxygen plasma treatment that can be used for two sensing materials that do not easily form Schottky junctions. In addition to this, a way to realize one-step synthesis to build 1D/2D heterostructures and a 1D/2D hybrid network structure based on the distinct qualities of the sensing materials themselves is proposed below.

**Figure 18:**
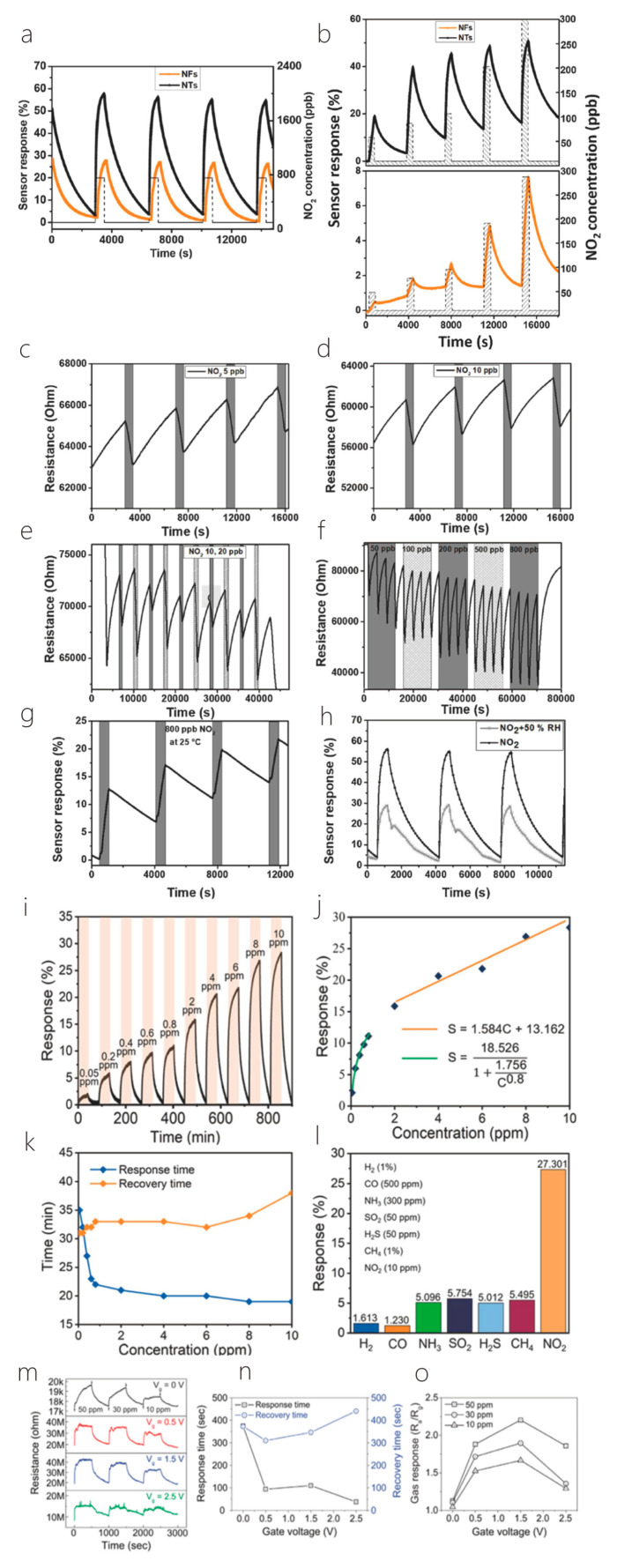
(**a**) Response of the WS_2_ NF (orange), WS_2_ NT (black) sensor to 800 ppb NO_2_ (four repeated response and recovery cycles) at an operating temperature of 150 °C. (**b**) Response and recovery of the WS_2_ NF (orange), WS_2_ NT (black) sensor to a pulse with increasing NO_2_ gas concentration (from 50 to 300 ppb). The sensor was operated at 150 °C. Repeated response and recovery cycles of WS_2_ NT nanomaterials to different NO_2_ concentrations. The operating temperature is set to 150 °C unless otherwise specified. (**c**) 5 ppb; (**d**) 10 ppb; (**e**) five repeated measurements of consecutive 10 and 20 ppb pulses; (**f**) four repeated measurements were made for concentration pulses in the range of 50 to 800 ppb; (**g**) four repeated measurements of 800 ppb pulses were performed while operating at room temperature; (**h**) three repeated measurements of 800 ppb were performed on a dry and 50%RH background. Reproduced with permission from the authors of [144]. (**i**) The room temperature dynamic sensing response of the gas sensors to NO_2_ under red light stimulation. (**j**) Sensitivity of Se/GeSexOy heterostructure sensor as a function of NO_2_ concentration: 0.05–10 ppm. (**k**) The heterostructure’s reaction and recovery time in relation to the concentration of NO_2_. (**l**) The selectivity of the sensor towards H_2_ (1%), CO (500 ppm), NH_3_ (300 ppm), SO_2_ (50 ppm), H_2_S (50 ppm), CH_4_ (1%), and NO_2_ (10 ppm). Reproduced with permission from the authors of [9]. The gas sensitivity performance of NO_2_ gas sensor based on 1D SnO_2_-2D SnSe_2_ heterostructure is improved by introducing backgate bias. (**m**) Dynamic resistance curves of a gas sensor based on 1D SnO_2_-2D SnSe_2_ hybrid nanowire network for different NO_2_ gas concentrations of 10–50 ppm at room temperature. Electrical characteristics evaluated with V_DS_ = 1 V and V_g_ = 0, 0.5, 1.0, 1.5, 2.0, and 2.5 V. (**n**) Response and recovery times as a function of gate voltage at a concentration of 50 ppm NO_2_ gas and at room temperature. (**o**) Comparison of NO_2_ gas sensing response at different backgate bias and gas concentrations of 10–50 ppm. Reproduced with permission from the authors of [34].

Alagh et al. [144] reported a two-dimensional layered WS_2_ nanosheet assembled on a one-dimensional WS_2_ nanostructure for the ultrasensitive detection of nitrogen dioxide. The result of such an experiment shows that this sensor demonstrated a stable, reproducible, and significant response to nitrogen dioxide at ppb concentrations (detailed proof is given in Figure 18a–h), which also illustrates that the sensor occupied the characteristics of the highest sensitivity, with an unparalleled ultralow detection limit below 5 ppb. However, such a sensor can only detect 800 ppb of NO_2_ when operated at room temperature (25 °C). In another study, Tang et al. [9] fabricated a p-type 1D/2D heterostructure made of GeSe_x_O_y_ nanosheets and Se bands using a single-step synthesis managed by the peel-nucleation method, which could detect NO_2_ below room temperature. Because of the type II band alignment structures that were found in heterogeneous structures with an effective bandgap energy of ~1.32 eV, this kind of sensor could cover the full visible spectrum. The result shows a ~27.3% response amplitude to 10 ppm nitrogen dioxide in red light irradiation, a sub-ppb detection limit, complete reversibility, excellent selectivity, and long-term stability of >3 months (Figure 18i–l). Similarly, Kim et al. [33] reported the fresh structure of a CO detection sensor below RT, which was devised by using a Au-modified n-type SnO_2_/p-type WS_2_ composite. The result shows that the Au-modified 10 wt.% SnO_2_-WS_2_ composite gas sensor has p-type behavior and is the most sensitive to CO gases. After analyzing the work function of P-type WS_2_C, it was found that the chemical sensitization of Au NPs resulted in an enhanced sensor response in the ternary composite. In gas sensing in self-heating mode using Joule heating, the likelihood of a Au-modified 10 wt.% SnO_2_-WS_2_, low-power CO gas sensor was demonstrated. The sensor response at 60% relative humidity was 84% of that in dry conditions, indicating that CO sensing is likely in humid environments operating at room temperature. Seo et al. [34] also fabricated a kind of gate-controlled sensor to detect NO_2_ making use of a 1D–2D hybrid nanowires network at room temperature. The report illustrated that transient resistance at different nitrogen dioxide focus was measured at room temperature when a gate bias (i.e., V_g_ = 0.5, 1.5, and 2.5 V) was applied. The gas sensor showed very good response (over 50%) compared with the response without gate voltage (Figure 18m). In addition, as shown in Figure 18n, the response and recovery behavior also improved as the gate voltage (1.5 V) increased. When the gate voltage was 1.5 V, the recovery time upgraded from 373 s to 346 s, and the response time was reduced from 375 s to 110 s. This suggests that the backgated voltage reduces the energy barrier adsorbed at the reaction site, resulting in an enhanced reaction of nitrogen dioxide gas molecules to the nanowire surface at room temperature. Figure 18o shows the response to diverse nitrogen dioxide gas concentrations at room temperature as a function of backgate voltage. The optimal gas response obtained was 2.2 (R_a_/R_g_) when exposed to 50 ppm nickel dioxide gas with V_g_ = 1.5 V at room temperature. This result is almost double the 1.13 (R_a_/R_g_) gas response without applying a gate bias.

**Figure 19:**
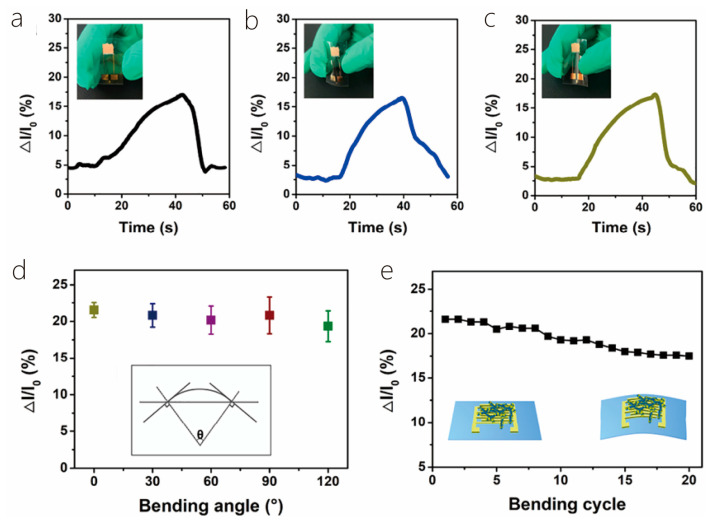
(**a**–**c**) The picture shows the dynamic ethanol response recovery curve of s BW/TiO_2_-3HNF-based flexible sensor under different bending states. The inset shows an optical image of a flexible sensing device with different bending states. (**d**) The sensitivity of the flexible sensor piece based on BW/TiO_2_-3HNFs varies with the bending angle. (**e**) Long-term stability measurement of flexible sensor elements based on BW/TiO_2_-3HNFs. Reprinted with permission from the authors of [138].

Therefore, they improved the gas sensing performance at room temperature by constructing a one- and two-dimensional hybrid nanostructure of the SnSe_2_ layer and a tin oxide nanowire network, and by controlling the backgate bias (V_g_ = 1.5 V). By reducing the energy barrier adsorbed at the reaction site controlled by the backgate bias, the response time is greatly reduced. Therefore, we believe that this research will assist in enhancing the effectiveness of gas sensors operating at room temperature. Besides, Jahangir et al. [32] proposed a diode/FET device based on a graphene/InN NW heterojunction to detect toxic gas. InN NWs have a very high carrier concentration on their surface, which makes it difficult to construct a real Schottky connection with graphene. In order to facilitate the creation of a rectifier junction, the NWs are, therefore, momentarily exposed to oxygen plasma, which creates a thin coating of In_2_O_3_ and partially passivates the surface. The result shows that such transistors exhibit excellent sensitivity to several common pollutants and toxic gases, such as nitrogen dioxide and ammonia. Heterojunction devices, when containing partially surface-passivated NWs, provide better sensitivity to specific gases than ordinary NW devices because of the presence of a larger and spatially homogeneous Schottky barrier, which can be modulated by gas molecules. The field effect version of the device allows further adjustments to sensitivity and discernment limits, including low ppb for nitrogen dioxide and low ppm for ammonia, which is extremely encouraging. What is more, in the field of flexible sensing devices, Wang et al. [138] devised a highly sensitive and superb mechanically steady flexible sensor device with a typical one-dimensional heterostructure. One-dimensional heterostructured nanofibers are wrapped in a 2D Bi_2_WO_6_ nanosheets (defined as BW NSs) with one-dimensional titanium dioxide nanofibers (NFs), exhibiting a multichannel sensitization effect. Combined with its extraordinary structural characteristics, grain boundary effect, and heterogeneous interface effect, the active site is synergistically increased, and the charge transformation is enhanced. In consequence, the device has high sensitivity (21.6%), quick response/recovery time (15.0/10.1), and low detection limits (500 ppb) at room temperature, analogous to original titanium dioxide NFs, BW NSs, and other 1D Bi_2_WO_6_/TiO_2_ hetero-nanofiber (defined as BW/TiO_2_ HNFs)-based devices. It has fine reproducibility and mechanical durability for ethanol gas molecules (19.8% at 60° after 20 cycles). Figure 19 effectively proves the above claim and has practical application prospects. The introduction of heterogeneous interfaces and grain boundaries gives a new approach to achieving very sensitive, stable, and flexible sensor devices.

For the purpose of adapting gas sensors to a wide range of application scenarios, higher demands are placed on their inherent temperature stability. Gas sensors operating at room temperature can be used in many applications, including indoor air quality monitoring, industrial gas detection, fire alarm systems, environmental pollution monitoring, etc. However, the development of their sensing properties remains a major challenge, and there is an urgent need to construct nanostructures with specific areas, crystal defects, and optimized carrier concentrations to improve low-temperature sensing properties [34]. It is known that the control of the reactive sites and charge concentration of the sensing material is a key element in determining the sensing performance of gas sensors operating at room temperature, and the shortcomings of the current gas sensors in terms of slow response and recovery time can be enhanced by starting from the optimization of the reactive sites for carrier transfer and their concentration to improve the oxidation rate at high operating temperatures. In addition to this, its irreversible sensing behavior is one of the current challenges, calling for researchers to find suitable sensitive materials for the one-step synthesis of gas sensors based on 1D/2D heterostructures. Flexible sensors are a current research favorite, but the activity and durability of current sensing materials used for the adsorption and reaction of gas molecules are insufficient, and there is a pressing need to develop high-performance active materials. According to the current research, it must be pointed out that 1D/2D heterostructures demonstrate good performance in the field of flexible electronics, which provides an effective direction for the development of flexible sensor devices [138].

### 4.2. Pressure and Strain Sensors

A pressure sensor, also known as a pressure transmitter or pressure transducer, is a device that measures the pressure of a gas or liquid, usually based on the piezoelectric effect, capacitance effect, strain gauge technology, or other principles. When pressure is applied to the sensitive element of the sensor, it causes a change in electrical properties, which is then converted into an electrical signal, and is widely used across many different sectors, including medical devices, industrial process control, the automobile industry, and environmental monitoring. Strain sensors, also known as strain gauges, are devices used to measure the deformation of materials under the action of external forces, measuring strain by detecting changes in electrical properties caused by material strain and displaying unique advantages within the domains of structural environmental observation, mechanical engineering, aerospace, and manufacturing. With the gradual deepening of research, pressure sensors and strain sensors will play a more significant role in the field of intelligent manufacturing, Internet of Things, and automation control. The introduction of new materials and technologies will further improve the performance and application range of sensors to meet a wider range of needs.

Currently, most force sensing devices are passive and overly dependent on external control. Due to the high integration of transistor-based transistors and their greater compatibility with CMOS systems, the integration of force sensing materials or structures onto FETs is one of the options currently considered to enable high-resolution active force sensors. According to the research, the hybrid dimension transistor is one of the current possibilities for sensing small pressures and amplifying piezoelectric signals, but at this stage, the research is still in the phase of investigation, the mechanism of the new structure is still not very clear, and it is necessary to research the fabrication process and the integration of the new hybrid dimension transistor further. Among them, 1D–2D transistors are candidates for future adaptive force mapping systems, and their integration into smart sensing networks has received much attention [10]. However, in the long run, better quality and more promising materials can be explored in terms of piezoelectric gate materials and 2D channel materials; to be able to design a good heterogeneous interface between the two, the doping levels of the 1D and 2D materials still need to be improved and optimized, which puts higher demands on the doping scheme and the research on the figure of merit.

**Figure 20:**
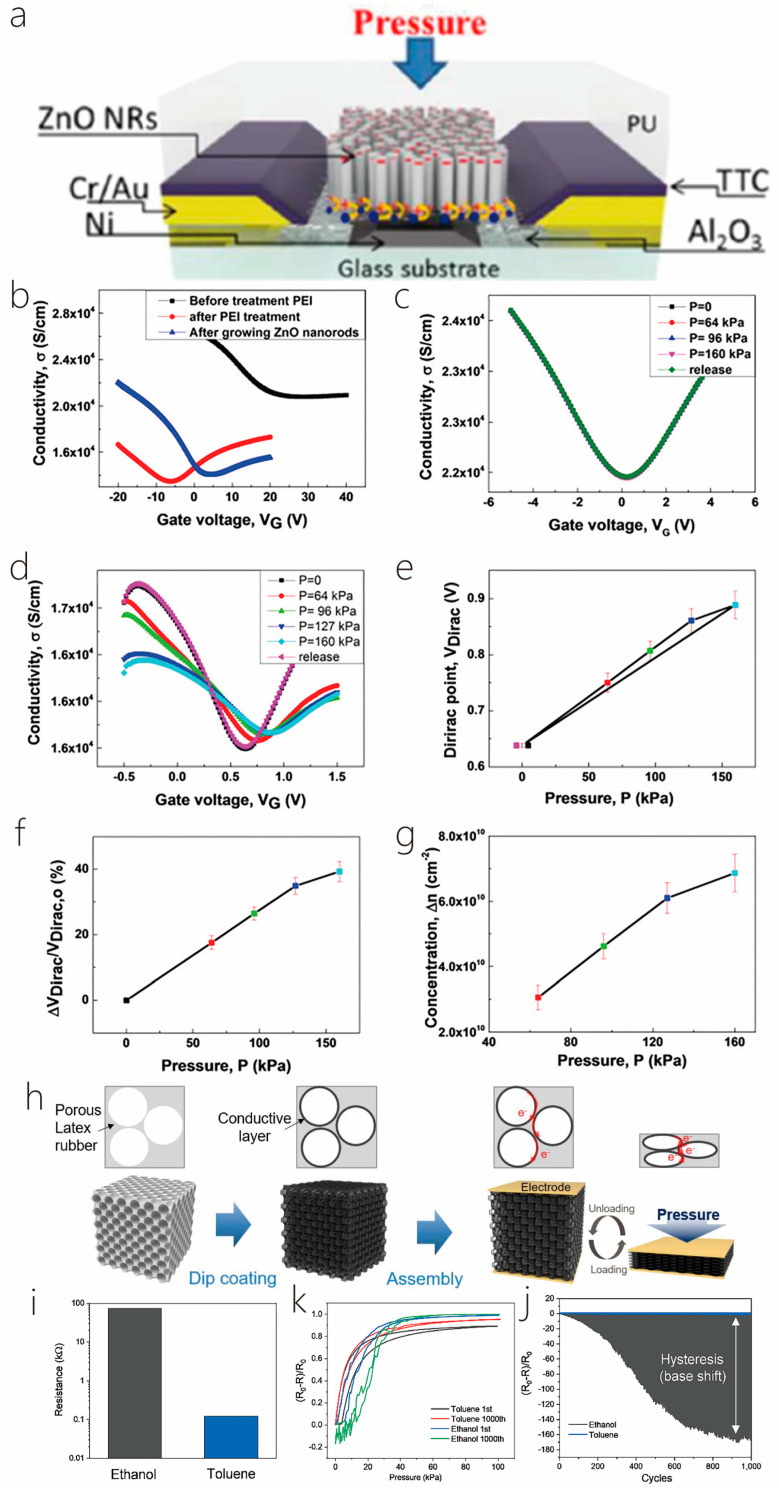
(**a**) Side profile view of a mixed-channel GFET. (**b**) Transfer characteristics of mixed-channel GFETs after each completion step, (**c**) Transfer characteristics of GFETs with Gr channels at different pressures, (**d**) Transfer characteristics of mixed-channel GFETs under different pressures. The source drain voltage (V_D_) was fixed at 1 V. (**e**) The Dirac point of transferred from Gr to ZnO-NR in the hybrid-channel GFET at different pressures; (**f**) Sensitivity of transferred from Gr to ZnO-NR in mixed-channel GFET at different pressures; (**g**) Electron concentration transferred from Gr to ZnO-NR in mixed-channel GFET at different pressures. Reproduced with permission from the authors of [123]. (**h**) Illustrative diagram of the manufacturing process of piezoresistive pressure sensor. (**i**) Electrical resistance of latex rubber coated with ethanol and toluene, respectively. (**j**) Hysteresis loops of pressure sensor based on latex rubber coated with ethanol and toluene, respectively. Due to the high resistance from the uncoated region, a noisy signal was observed from the pressure sensor coated with ethanol. (**k**) Pressure sensor durability tested at high pressure (100 kPa) for 1000 loading/unloading cycles. Reproduced with permission from the authors of [151].

As for the pressure and strain sensors, since 1856, when Thomson first reported that the resistance of iron and copper varies with time, strain sensors have been broadly manufactured through the use of diverse metals and semiconductors [111,145,146,147,148,149]. The resistance R is a characteristic of the conductor and can be written as R = ρL/S, where L is the length of the conductor, and S is the average cross-sectional area of the conductor. When stress is applied to a wire, its resistance changes. In addition to the geometry, changes in resistivity also play a significant role in changes in resistivity. The cross-sectional area of the bulk material decreases related to the longitudinal strain of the Poisson’s ratio ν. Poisson’s ratio fluctuates from 0.20 to 0.35 for most metals, while anisotropic silicon fluctuates from 0.06 to 0.36 [145,149]. In 2013, the strain transducer market is expected to exceed USD 4.5 billion. With the relentless pursuit of low-cost and miniaturized devices, traditional silicon semiconductors are facing challenges, and recent research on strain gauge sensors has mainly focused on nanoscale materials [145]. Dang et al. [123] triumphantly manufactured a graphene-based field effect transistor (GFET) with a mixed channel of zinc oxide NRs/Gr. The cross-sectional view of the hybrid-channel GFET is seen in Figure 20a. Figure 20b–d demonstrates the transfer characteristics of mixed-channel GFETs in different environments. The positive shift of the Dirac point of the mixed-channel GFET is 0.25%/kPa, and the channel conductivity changes significantly; whereas, the GFET without zinc oxide NRs has no response. Due to the piezoelectric effect of zinc oxide NRs, charge transfer at the interface between Gr and zinc oxide NRs results in changes in the Dirac point and the modulation of conductivity of holes and electron branches. The electron concentration transferred from Gr to zinc oxide NRs was calculated as 4 × 10^8^ cm^−2^ kPa^−1^. Mixed-channel GFETs without biased V_G_ also responds to boosting. Figure 20e–g depicts the Dirac point, sensitivity, and electron concentration migrated from Gr to ZnO nanorods in the hybrid-channel GFET across different pressures. The mixed-channel GFET’s excellent performance coupled with low power usage emphasizes its promising potential for future smart device pressure sensors. Li et al. designed a textile yarn strain gauge equipped with a one-dimensional–two-dimensional nano hybrid strain sensing sheath with superior tensile ability. This ultrastretchable strain sensor is highly sensitive (gauge factors (GFs) of 139.6 and 198.8, 0.01% and 125%, respectively) and has a wide and consistent strain sensing range (0.01%–125%) from microscale to macroscale. The result shows that during tensile/deformation, microcracks are generated and propagated in the layered structure of graphene nanoplates (GNPs), causing significant changes in resistance, while Ag NWs bridge adjacent GNPs to partially allow for applied stress and enhance strain [150]. Similarly, Lee et al. also fabricated synthesized hybrids that could be utilized to manufacture pressure sensors [151]. An illustrative diagram of the piezoresistive pressure sensor manufacturing process is demonstrated in Figure 20h. The outcome demonstrates that the robust bonding connection between the conductive stratum and the interface of the conductive material/elastomer substantially diminishes the sensing delay to a minimal 1.33% and considerably enhances stability in the sensing performance for up to an extensive number of 10,000 cycles under elevated pressure conditions (100 kPa). Figure 20i–k shows the electrical functionality of the original MXene pressure gauge depending on the type of solvent used in the coating solution. What is more, the synthesized hybrids with excellent interfacial adhesion function as conductive sensing materials for pressure sensors. Strong interfacial adhesion within the conductive layer and the conductive layer/elastomer interface produces minimal defects, allowing for exceptional electrical stability and incredibly minimal hysteresis even under repeated high-voltage loading. By integrating each pixel into an 8 × 8 sensor array, large-area pressure sensors have sensor-to-sensor uniformity. The high classification accuracy (>95%) of various seating postures using machine learning algorithms promotes the potential of future healthcare monitoring systems. 

**Figure 21:**
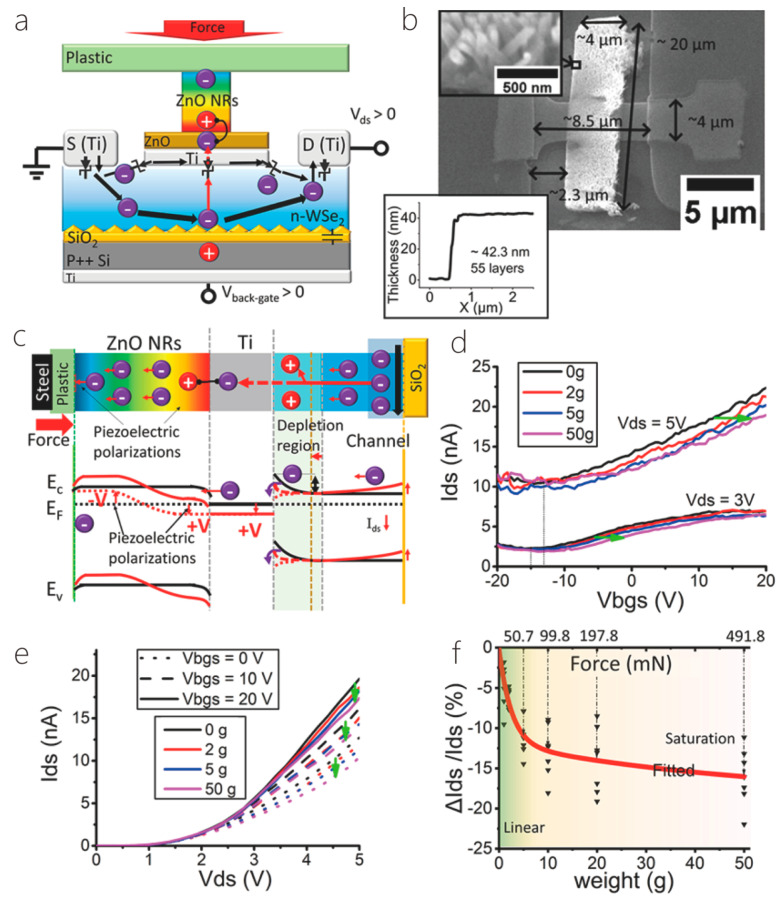
(**a**) Schematic diagram of device structure and working mechanism of ZnO-Ti-WSe_2_ piezoelectric gate transistor. (**b**) The large figure shows the SEM image of ZnO-Ti-WSe_2_ piezoelectric gate transistor. The inset figure shows the AFM profile of WSe_2_ sheet thickness and the high-power SEM image of ZnO-NR at 45° tilt. (**c**) The figure shows the energy diagram of the piezoelectric gated Schottky barrier mechanism, and the lower red line is the energy band diagram after applying strain to the ZnO NR. (**d**) Transfer characteristics of ZnO-WSe_2_ piezoelectric gate transistors under different weight loads. (**e**) Output characteristics of ZnO-WSe_2_ piezoelectric gate transistors under different weight loads. (**f**) The function of the applied weight is calculated by the drain source current change ((ΔIds/Ids)%); scattering points come from transfer and output characteristics. Reproduced with permission from the authors of [10].

Lee et al. fabricated a large 8 × 8 pressure sensor array. Conductive nylon mesh bonded by carbon black ink was used as an interconnector in the sensor array. The use of this interconnector reduced mechanical coupling in adjacent sensors with great effectiveness. Pressure of 40 kPa was applied to each sensor (64 sensors total), so sensor-to-sensor uniformity was detected one by one. The shift in relative resistance was highly uniform, with an average of 0.887, a standard deviation of 0.066, and a coefficient of variation of 7.5%. In addition, they also detected the reaction while under simultaneous pressure applied to multiple sensors. This relative resistance modulation plot accurately represents the applied pressure pattern. In the end, a large area pressure sensor array was used as an intelligent cushion for attitude monitoring the drag change of each pixel in real time in different seating positions. In another study, Geng et al. fabricated a mixed-dimensional piezoelectric-gated transistor that could be utilized as a forcing sensor [10]. The forcing sensor was made up of one-dimensional piezoelectric zinc oxide nanorods (NRs) used as a gate control and a multilayer tungsten diselenide (WSe_2_) used as a transistor channel. The result shows that applying mechanical force to piezoelectric NRs can cause drain source current variation (ΔIds) on the WSe_2_ channel. It was found that different doping types of WSe_2_ channels resulted in different orientations of ΔIds. Geng et al. also proposed two types of an actual device with a different type of channel: a piezo-gated transistor with a p-type-doped WSe_2_ channel (Dev.1) and a piezo-gated transistor with an n-type-doped channel and a Ti intermediate layer (Dev.2). Figure 21 displays a schematic of the device structure of the ZnO-Ti-WSe_2_ piezoelectric gating transistor and its operating characteristics.

In both devices, a positive piezoelectric voltage is produced at the bottom side of zinc oxide by the pressure, which, thus, causes a definite offset in the backgated transfer characteristics. However, there are significant differences in doping types of WSe_2_, which is thought to be the reason for the different direction of Ids changes. The WSe_2_ of Dev.2 is the original n-type doping; whereas, the WSe_2_ in Dev.1 has been observed to be p-type-doped, probably due to the xenon difluoride overcut. However, there are significant differences in the doping types of WSe_2_, which is thought to be the reason for the different direction of Ids changes. In the field of integrated FET for forcing detection, a new type of transistor has been introduced as a force sensing unit by Geng [35], which is a novel piezoelectric force sensing transistor based on a one-dimensional zinc oxide NR array.

Zinc oxide NR arrays have been selected as piezoelectric barriers for transistors owing to their great pressure sensitivity and relatively simple synthesis method. The reasons for choosing WSe_2_ due to the channel material are: (1) the doping level of WSe_2_ can be adjusted [152,153,154], which can provide a greater possibility to the channel; (2) compared with the other WSe_2_, it has a higher fracture strain, which may contribute to the flexible substrate [155]. When a mechanical force acts on zinc oxide NRs, the piezoelectric potential regulates the current flowing in the two-dimensional channel. The report shows that one-dimensional zinc oxide NRs are integrated with two-dimensional WSe_2_ transistors to obtain mechanical forces applied to piezoelectric NRs that can cause drain-to-source current changes (ΔIds) on a WSe_2_ channel. Different directions of ΔIds have been discovered to be caused by different types of stimulatory WSe_2_ channels. For manufactured devices, the pressure of the calibration weight is 2 g (g), resulting in a ~20% id increase in p-type devices doped with WSe_2_ channels, a 10% decrease in n-type-doped WSe_2_, and a Ti intermediate layer. The report also proposes two different examples of 1D–2D transistors: the first device (Dev (a)) has an etched WSe_2_ channel that may have p-type-doped behavior due to edge defects caused by xenon difluoride vapor [155]. The zinc oxide NRs are integrated into WSe_2_ FET by Dev (a) using a simplified method, wherein the zinc oxide NRs and the zinc oxide seed layer are in direct contact with the WSe_2_ channel, and there is no Ti adhesion layer. As for Dev (b), the original WSe_2_ is used as the channel of the FET, which is an N-doped FET. For Dev (b), there is a Ti metal layer between the ZnO seed layer and the WSe_2_ channel. This study shows that it is possible to adjust the sensitivity and polarization of 1D–2D transistors by adjusting the doping of the WSe_2_ channels, which is of great help to future high-resolution force sensing systems.

Defects in the semiconductor lattice affect the electronic and optical properties of the material, and defect engineering can be used to obtain the desired properties in semiconductors without complicated processing. One of these is the use of mild plasma treatments, which can improve device performance metrics. Various gadget designs, packaging, and mechanical coupling can affect the output of the sensing transistor, and the future also looks forward to more advanced 2D FET contact engineering for better packaging layers or package structures. Pressure and strain sensors with integrated transistors may rely on the transfer characteristics of the semiconductor channel and the piezoelectric properties of the sensing material, for which the capture of both places higher demands on the sensing material as well as the sensing structure. Also, pressure and strain sensors are a challenge to ensure accuracy and stability in extreme environments.

### 4.3. Optoelectronic Synaptic Devices

Modern electronic computers, all based on von Neumann architecture, have evolved rapidly over more than half a century to play an indispensable role in various fields of science and technology, always at a pace that is relatively consistent with the growing demand for low-power complex computation and parallel processing. However, the von Neumann bottleneck, i.e., the physical separation between the memory and the central processor, limits the use of traditional computing architecture for next-generation information electronics applications [156]. It is exciting to note that brain-inspired neuromorphic computing systems have a unique talent for combining storage and computation, responding efficiently to parallel processing with superior precision and memory. Unlike traditional von Neumann architecture, the biological brain has a large number of synapses that act as computational and storage units, allowing the brain to be efficient and fault-tolerant when dealing with emotion, learning, and thinking. This is one thing that conventional computers are not capable of. Therefore, researchers have invested a great deal of effort in simulating neuromorphic operations in electronics to achieve high parallelism and low power consumption. However, current micro- and nanoelectronics-based computing systems utilize devices where data processing and storage on the chip are still electronic. Similar all-electronic designs may impose limitations on the speed of operation and lead to an increase in power loss, resulting in significant energy loss during charging and discharging [157]. To escape these limitations, neuromorphic computing, an emerging research field that intersects optoelectronics with neuromorphic engineering, aims to push the boundaries between traditional semiconductor computing techniques and artificial neural computation and architecture. New accelerated optoelectronic–neuromorphic computing architecture can combine the benefits of information processing power with the considerable optical bandwidth at speeds similar to those of biovisual perception systems (BVPSs), which perceive a wide range of visual information through the retina of the eye. BVPSs perceive incident light through the retina of the eye, which carries a variety of visual information, and convert this information into neural impulses that are sent to the brain via optic nerve synapses. Electronic devices that mimic the functions of BVPSs, often referred to as photoelectrosynaptic devices, utilize light pulses to stimulate synapses, incorporate light perception into neurotransmission, enhance optical power and capture information such as wavelength and frequency, and have the ability to combine visual signal detection, information processing, and data retention [158]. Conventional photodetectors are constructed based on the photoelectric or photothermal effects of materials, which can convert light stimuli into electrical signals, but they are nonretainable, and once the light stimulus disappears, the light information is also eliminated; whereas, photoelectric synapses have memory and exhibit photoreceptor effects; even if the light signal disappears, they dynamically store their resistive states to realize their different synaptic properties. In recent years, rapid development in the field of nanoscale has led to the enhancement of the functionality of optoelectronic synapses relying on advanced material systems, among which the application of low-dimensional materials in the field of artificial optoelectronic synaptic devices has attracted much attention. Due to their tiny size, they offer an outstanding advantage in terms of light generation performance.

#### 4.3.1. 1D

One-dimensional nanostructures, which have only one dimension in the nanoscale range but offer another additional spatial dimension confined within it, pave a promising path for studying the nanoscale, with important implications for potential optoelectronic applications [158]. One-dimensional nanowire networks have taken the stage of neuromorphic systems due to their similarity to biological neural networks in terms of structure, information transfer, and computation. Due to their excellent optoelectronic properties, broad specific surface area, and small size to support high-density integration, they are widely used in photonic memories, intermediate resistive layers of optoelectronic memristors, and other fields. Particular attention is paid to the functional synaptic connections with synapse-like conductance changes and nonlinear dynamics occurring at the intersections of its nanowires in nanowire network-based devices. Outside of here, it exhibits a longer photogenerated carrier lifetime, excellent gas sensing capability, and asymptotic memory properties, providing a practical pathway for artificial synaptic devices: olfactory–visual, cotuned memory resistors [159]. O’Kelly et al. first used UV lithography to define a certain area of contact pads, followed by the spray deposition of a dilute TiO_2_ solution in deionized water, and finally, electron beam lithography fabrication to obtain titanium dioxide nanowire devices, which are capable of processing heterogeneous physical stimuli as associative memory responses. The experimental results demonstrate that the enhancement effect resulting from the temporal correlation of voltage and light stimuli is stable and can, therefore, be reproduced on multiple devices with a single nanowire structure, as well as expanding the number of inputs or dual-device output interconversions. For the first time, it is demonstrated that a material can realize time-correlated heterogeneous inputs, robustly delivering materials for neuromorphic sensing applications [160]. Shen et al. chose NiO, a p-type amorphous oxide semiconductor with excellent memristor performance, as the shell material to construct a 1D SiC@NiO core–shell structure, based on which they fabricated a NW network optoelectronic memristor, which solves the two problems associated with NW-based SiC memristors: they are easy to lose, and they do not store information effectively. Among them, the unique NW-NW cross junction provides a large surface area for effective charge transfer, and the oxygen vacancies of the shell structure can realize sustained photoconductivity. Due to their high-temperature stability and relatively comprehensive synaptic properties, SiC@NiO core–shell nanowire networks have promising applications in neuromorphic computing and artificial vision systems where artificial optoelectronic synapses are at high temperatures [159].

Optoelectronic synapses relying solely on individual 1D nanostructures may require additional complex processes when integrating with other electronic components in order to enhance the overall capability of the device structure. To further explore the potential of 1D nanomaterials and to solve this current situation, the binary assembly of nanowires, nanorods, or nanoribbons can be adopted as an effective collection of nanowires, nanorods, or nanoribbons. The mystery of this still needs to be investigated in detail.

#### 4.3.2. 2D

Two-dimensional materials with extremely thin structures that provide high stiffness and high tolerance to external stresses and strains are uniquely attractive for building slab portions of flexible electronic applications. The close correlation between electrical and optical stimulation and the electronic properties of 2D materials makes bionic optoelectronics based on 2D layered materials a hot research topic. In addition, 2D materials have the properties of electrical tunability, mechanical flexibility, and flexibility, which have made considerable achievements in the field of bio-optoelectronics, especially in the photostimulation of synaptic devices, which utilize their excellent physical properties. Examples of these materials, such as graphene (Gr), black phosphorus (BP), TMDs, etc., have attracted a lot of attention. Here, we will discuss several popular materials for your reference and understanding. Single-layer graphene has no bandgap and possesses disadvantages, such as low light absorption and high recombination rate, which hinders its application in optoelectronic synapses; hopefully, a graphene-based heterojunction will help to overcome these limitations. As an alternative, BP, which has the advantages of high hole mobility, wide tunable direct band gap, and UV sensitivity and defects caused by its oxidation, was used to construct the optoelectronic synapses. MXenes are the successors of graphene, and their compatibility with a variety of organic solvents further enhances their attractiveness [161].

Hao et al. provided broadband photoresponse through p-type BP channels, which were further modulated using ferroelectric HfZrO_4_ (HZO) to construct a photodetector defined by a ferroelectric structure with a field effect transistor structure to perform the device operation. As a result, the designed optoelectronic memory device can exhibit excellent electrical characteristics and synaptic functions by modulating the BP channel resistance, where the synergistic modulation effect expands the potential of BP for practical applications [162]. Cao et al. proposed a brain-like optoelectronic–synaptic thin-film transistor based on Ti_3_C_2_T_x_ MXene-TiO_2_ floating gate prepared using the solution processing method, which solves the problem of oxidative instability of MXenes and further improves the photoresponsive strength and storage capacity of the device. This study provides an effective method for constructing artificial brain-like systems [163]. Chen et al. proposed a bidirectional synaptic phototransistor based on a 2D ferroelectric semiconductor α-In_2_Se_3_, demonstrating the possibility of single- and mixed-color pattern recognition and its excellent mixed-color pattern recognition rate in a simulated neural network, which is of great promise for artificial vision systems [164].

The use of reliable synthesis methods is crucial for the integration of high-quality 2D materials into device architecture, where attention needs to be paid to the defects and side reactions triggered during the synthesis process. In addition to this, a single material alone cannot achieve the desired results, and the synergistic modulation effect resulting from enhanced integration can be used to extend the physical properties of the raw material to broaden its potential. Research into how to reduce the power consumption of photovoltaic synapses and how to effectively utilize the defective states to improve their photovoltaic properties is also needed.

#### 4.3.3. 1D/2D

One-dimensional materials have covalent bonding in the axial direction, and integration with 2D materials is not interfered with by dangling bonds in the mixed-dimensional vdW heterostructure. The heterojunction structure constituted by choosing an effective combination of appropriate 1D and 2D materials can provide excellent charge transfer channels to improve the responsiveness and performance of the device. Not only that, but it may also effectively disperse the stress and reduce the defect density, contributing to the improvement of the stability and lifetime of the optoelectronic synaptic devices. Heterojunction-based synaptic devices exhibit exceptionally sensitive photoconductive properties, enabling efficient optical signal modulation.

A photonic synaptic device based on an (Al,Ga)N nanowire/graphene heterojunction was successfully prepared by Zhou et al. Among them, the (Al,Ga) N nanowires exhibit a stable and sustained photoconductivity effect at room temperature, which, combined with excellent properties such as high stability and large surface area ratio, can improve the memory duration of the photocurrent. The good conductivity and transmittance of graphene in the UV range can be easily controlled by using an absorber with an appropriate bandgap, making graphene suitable for applications to connect NWs and conductive currents. The device is promising for low-power applications [36]. Ni et al. designed and fabricated an optoelectronic neuromorphic transistor with a p-i-n hybridized heterojunction structure consisting of quasi-one-dimensional ZnO NWs with 2D 2,7-dioctyl[1]benzothieno[3,2-b][1]benzothiophene (C8-BTBT)/poly(methyl methacrylate) (PMMA) phase separation membranes, which were prepared in combination. In this case, the effective integration of optical sensing and electrical processing takes advantage of the multidimensional heterojunction combining the properties of each party. Not only that, this low-dimensional hybridized heterojunction can significantly improve the optical performance of the photovoltaic neural device, which can be investigated further to improve the optical sensitivity of the device. The device can respond to low-intensity UV light and changing carrier orientation and has advantages in sensing patterns and further memorizing images, as well as mechanical plasticity for immediate switching [11].

Although 1D/2D heterojunction structures have many advantages in the field of optoelectronic synapses, more precise fabrication processes are needed to prepare high-quality 1D/2D heterojunctions in order to utilize their excellent performance. In addition, heterojunction structures can be designed to break the limitation of single-type semiconductor materials or single-channel structures, which provides an effective and feasible way to sense optical and electrical stimuli. The application of 1D/2D heterojunctions in photoelectric synapses still needs to be improved and explored.

### 4.4. Biosensors

A biosensor is a device that uses biological substances as recognition elements, converts biochemical reactions into quantifiable physical or chemical signals, and, thus, plays a role in detection and monitoring. As an intersection of biological sciences and engineering sciences, it is developing more and more rapidly in biological and chemical analysis, biomedical diagnostics, and environmental detection [165]. Among them, the development of semiconductor material technology has made a great contribution. As the manufacturing technology of semiconductor materials is constantly improving, they have excellent electrical characteristics and show good chemical and mechanical stability as well as supporting multiple sensing mechanisms, which can achieve high-precision measurements and multifunctionality when applied to biosensors. Through special treatment, such as surface modification and functionalization, semiconductor materials can be specifically combined with specific biomolecules to achieve the selective detection of target molecules. Therefore, the enhancement of semiconductor technology is expected to improve the ability of bioengineering applications [166]. The applications of 1D, 2D, and 1D/2D heterojunctions in the field of biosensors are described next.

#### 4.4.1. 1D

One-dimensional nanostructures can provide more surface-active sites for biomolecule binding due to their large specific surface area, thus improving the sensitivity of biosensors. Not only that, certain 1D nanostructures can provide channels with nontoxicity and good biocompatibility for electron transfer, providing more selectivity for the construction of high-performance electrochemical sensors. The ordered nanoarrays with a good microenvironment and strong electron transfer capability provide a new path for the application of biosensors. In conclusion, 1D nanomaterials have a broad application prospect in this field.

Yang et al. successfully used the citric-acid-assisted annealing method to prepare ZnO nanorods for a glucose detection biosensor. Unlike general glucose biosensors, there is a two-part linear detection region, and the affinity of denatured ZnO nanorods for glucose is effectively enhanced [167]. In contrast, Xu et al. prepared 1D CuO nanotube arrays using the anodic oxidation method and applied them for glucose detection. Compared with the above simple nanorods, the nanorod arrays possess a larger surface area and the efficient diffusion rate of biomolecules [168]. Zhou et al. designed and fabricated gold nanoparticles sensitized by ZnO nanorods arrays, which were subsequently processed for dopamine sensing, and the resulting electrochemical sensors integrated the advantages of gold nanoparticles and ZnO [169].

Despite the great potential of 1D semiconductor materials for biosensor applications, a number of challenges remain. For practical applications, how to effectively integrate small-sized 1D materials into sensors is still a challenge, and the question of whether 1D materials are compatible with biological and nontoxic samples is not to be overlooked.

#### 4.4.2. 2D

Two-dimensional materials have a unique thin-layer structure and very high specific surface area, which makes them suitable for high-density integration as well as effective for surface functionalization. Moreover, their physical and chemical properties can be tuned by layer number, doping, or other means, which can be adapted to biosensors with different needs. Two-dimensional semiconductor materials have good mechanical flexibility, and biosensors based on them are suitable for the fabrication of portable and wearable devices. Not only that, surface modification, protective coating, encapsulation, and the specific molecular functionalization of 2D nanomaterials extend their biomedical applications.

Jiang et al. designed the 2D Ti_2_C MXene-induced photocurrent polarity switching of Ag_2_S quantum dots for the construction of biosensors with ultrasensitive detection, which overcomes the hazards of conventional toxic semiconductors and offers a possibility to fabricate high-performance polarity-reversal-mode photoelectrochemical biosensors for the excavation of novel-sized photocurrent polarity switches [170]. Zhang et al. used the coupling of DNA tetrahedra and biotin–streptavidin (B-SA) to functionalize MoS_2_ field effect tubes, which showed excellent sensitivity to target proteins. Although practical testing was lacking in this study, functionalized MoS_2_ FET devices offer a potential avenue for the immediate diagnosis of complex diseases at low concentrations [171].

Although 2D semiconductor materials have good biocompatibility, they may be potentially toxic for long-term behavior in complex biological environments. We also need to be aware of their biodegradability to avoid toxic byproducts [172]. In addition to this, the nonspecific adsorption of 2D materials limits their biomedical applications and can interfere with biosensor monitoring. In order to minimize unwanted biological reactions and, at the same time, ensure the physicochemical properties of 2D materials, more precise engineering of their surfaces is required.

#### 4.4.3. 1D/2D

The 1D and 2D materials themselves have a high specific surface area, and the two form a heterojunction to further increase the active sites and improve sensitivity to monitor biomolecules. Various functionalization modifications to the surface of the heterojunction structure can enhance the selectivity of the target biomolecules and improve the accuracy of the biosensor. Not only that, but biosensors can also combine the properties of 1D and 2D materials to realize a multifunctional collection. Certain heterojunction structures also have a self-healing ability, which can improve the lifetime and reliability of the sensor. Biosensors based on 1D/2D heterojunction structures can operate at a lower power consumption level, which is important for achieving long-term operation.

Jiang et al. synthesized 1D-SnO_2_/2D-SnS_2_ heterojunctions using a hydrothermal method to effectively immobilize SnO_2_ nanorods on top of SnS_2_ nanosheets. Because heterojunction structures based on 1D/2D materials can easily obtain more active sites and reduce the grain boundary barrier, the interactions between the structures offer the possibility of obtaining s superior performance for the devices. The resulting 1D/2D SnO_2_/SnS_2_ heterostructure facilitates enhanced charge transfer, the separation of photogenerated carriers, and the sensitization of SnO_2_, which broadens the range of the effective use of light. The sensor for analyzing Lincomycin was subsequently constructed by combining PtPd nanoparticles modified with CeO_2_ as a signal amplifying nanozyme [37]. Zhao et al. used a combination of a hydrothermal pathway and controlled oxidation to together synthesize 1D/2D heterojunction structures consisting of 1D Cu NWs and 2D Cu_2_O nanosheets, which were later assembled as electrodes for glucose sensors. Among them, the Cu core becomes a channel for fast electron movement, and the Cu_2_O shell layer acts as an electron transfer medium for glucose detection. The intelligent integration of the two has a minimal lattice mismatch to facilitate electron transfer, breaking through the limitation that a single material cannot improve performance [12].

Although 1D/2D heterojunction structures have significant advantages for biosensor applications, the interfacial engineering between 1D and 2D materials needs to be further improved, and the process of accurately preparing and integrating 1D/2D heterojunction structures is complicated, limiting large-scale production. Moreover, their high area ratios may easily adsorb pollutants and affect the performance of the sensors. Measures such as improving the material preparation process, optimizing the sensor design and enhancing the surface treatment may contribute to the development of 1D/2D heterojunction structures in the field of biosensors.

## 5. Summary

This paper reviews the synthesis methods of 1D and 2D materials and 1D/2D heterostructures and their applications in photodetectors and sensors. For the synthesis of 1D and 2D materials and 1D/2D heterostructures, several methods are reviewed, including CVD, PVD, hydrothermal, and unique growth methods for 1D/2D heterostructures. For applications in photodetectors, different types of photoconverters have been fabricated on different semiconductors, including metal oxides and TMDs. We systematically discussed the working mechanism of these metal-oxide-based devices. We also thoroughly discussed the 1D/2D heterostructure application in sensors. In addition, a number of emerging technologies that are widely used to optimize device performance are discussed. This leads to the need for technology to face the manufacture of more complex devices with significantly enhanced performance and outstanding functionality. Although 1D/2D heterostructures demonstrate many unique advantages in photodetector and sensor applications, they also face some shortcomings and limitations. The fabrication of high-quality 1D/2D heterostructure materials requires complex processes and precise control, and their performance is mostly reliant on the quality of the interface, which places higher demands on the synthesis and fabrication processes. Two-dimensional materials with high light absorption and high mobility capabilities and 1D/2D heterostructures are expected to show new functions in new optoelectronic device applications. However, the difficulty of manufacturing high-quality 1D–2D heterostructures limits their application in photodetectors. The manufacture of high-quality 1D/2D heterostructure materials requires complex processes and precise control, and their performance is highly dependent on the quality of the interface, which puts higher demands on the synthesis and manufacturing processes. For example, in the practical application of one-dimensional/two-dimensional hybrid nanostructures, one-dimensional nanostructures must grow uniformly on two-dimensional layers. Although several methods for preparing position-controlled 1D/2D nanostructures have been reported, many integrated devices require the production of more scalable and reproducible position-controlled 1D nanostructures on large size 2D nanomaterials. Therefore, homogenization and mass production methods for 1D/2D heterostructure materials to achieve low-cost commercial applications remain to be investigated. In addition, the assembly of different materials and precise control of interface characteristics are critical to the operation and performance of solid-state devices. So far, mixed-dimensional one-dimensional/two-dimensional heterostructures have been fabricated using various physical assembly methods. However, direct epitaxial growth methods, which have obvious advantages in preparing large-scale products and obtaining perfect interfaces, have rarely been studied. In this regard, the mixed-dimensional vdW heterostructure presents unique challenges and opportunities. Unlike bulk inorganic or fully two-dimensional vdW heterostructures, in which all components can be grown in the same reactor by switching precursors during a single growth process, synthesizing two materials with different atomic arrangements and thermal stability ranges in a single process is extremely challenging. Photodetectors generate heat during operation, especially at high power densities, and how to effectively manage and dissipate heat is one of the keys to ensuring the performance stability of 1D/2D heterojunction photodetectors. Improving the slow response and recovery time of current gas sensors is also one of the outstanding issues, and there is an urgent need to develop high-performance sensing materials to make up for the lack of activity and durability. One-dimensional and two-dimensional mixed-dimensional transistors are still in the investigation phase, and the mechanism has yet to be clarified, which has slowed the progress of pressure and strain sensors. Currently, theoretical modeling based on 1D/2D heterostructures is equally challenging for researchers. Due to its multidisciplinary nature, it is necessary for researchers in various relevant fields to focus their efforts on fully exploring the potential applications of this heterostructural material. For example, several well-known theoretical modeling tools based on density functional theory (DFT) and mixed functional and mixed heterogeneous interface approximation can help us to better understand the physical fields involved in such heterogeneous structures. In the future, with the discovery of new materials, the improvement of synthesis techniques, and the optimization of device processes, the research and application of 1D/2D heterostructure materials will continue to be deepened, and their applications in various advanced technologies will become more widespread and mature. We hope that this review will give insights into the development of 1D/2D heterostructures, especially their synthesis and application in optoelectronic devices.

## Figures and Tables

**Table 1:** The synthesis methods and characteristics of some of the materials exemplified in the table below are accompanied by references.

Material	Synthesis	Characteristic	Reference
1D CNTs	CVD	High anisotropic thermal conductivity and current-carrying properties	[13]
1D ZnO	CVD	High surface-to-volume ratio, high light transmission in the visible zone	[14]
	PVD		[15]
2D MoS_2_	CVD	Excellent mechanical properties	[16]
2D NiS_2_	CVD	Excellent electrical conductivity, special magnetic properties	[17]
2D TiS_2_	CVD	Good thermoelectric properties, localized surface plasmon resonances (LSPRs) in the NIR range	[18]
1D Bi_2_S_3_/2D MoS_2_	CVD	Fast photoresponse, high mobility	[5]
1D ZnS	PVD	Excellent transport properties	[19]
1D In_2_O_3_	PVD	Transparent conductive oxide	[20]
1D SnO_2_	PVD	High specific surface area and excellent gas-sensitive properties	[21]
2D PbI_2_	PVD	Good photosensitivity, good photoresponsivity	[22]
2D SnS	PVD	High sensitivity to temperature	[23]
1D Te/2D TMDC	PVD/CVD	Effective interlayer charge transfer, broadband saturable absorption	[6]
1D Sb_2_Se_3_/2D PtSe_2_	PVD	Extended low-temperature growth, increased light absorption in the midinfrared	[24]
1D Na_2_Ti_3_O_7_	Hydrothermal methods	Good thermal stability	[25]
2D α-Ni(OH)_2_	Hydrothermal methods	High specific surface area, excellent electrochemical redox properties	[26]
1D TiO_2_/2D MoS_2_	Hydrothermal methods	Good separation of electron–hole pairs	[27]
1D WO_3_/2D NiCo_2_O_4_	Hydrothermal methods	Excellent gas-sensitive properties	[28]
1D Sb_2_Se_3_/2D WS_2_	Two-step Van Der Waals Epitaxy	Obvious current rectification behavior	[7]

**Table 2:** The following table demonstrates the characteristics and applications of some of the 1D/2D heterostructures and is followed by references.

Material	Characteristic	Application	Reference
1D Bi_2_S_3_/2D MoS_2_	Fast photo response time	Photodetector	[5]
1D ZnO/2D WSe_2_	High rectification ratio	Photodetector	[29]
1D CuO/2D MoS_2_	High carrier mobility, strong rectification characteristics	Photodetector	[30]
1D Te/2D ReS_2_	Strong light absorption, high photoresponsivity	Photodetector	[31]
1D InN/2D graphene	High carrier mobility and surface sensitivity	Gas sensor	[32]
1D SnO_2_/2D WS_2_	Low voltage, low power consumption	Gas sensor	[33]
1D SnO_2_/2D SnSe_2_	High sensitivity, selectivity and efficiency	Gas sensor	[34]
1D ZnO/2D WSe_2_	High resolution force	Pressure and strain sensor	[35]
1D (Al,Ga)N nanowire/2D graphene	Broadband, low power consumption	Optoelectronic synaptic devices	[36]
1D SnO_2_/2D SnS_2_	High photon to electron conversion efficiency and stability	Biosensor	[37]
1D Cu/2D Cu_2_O	Good performance stability and high charge transfer efficiency	Biosensor	[12]

## Data Availability

No new data were created or analyzed in this study. Data sharing is not applicable to this article.

## References

[1] Jeon P.J., Lee Y.T., Lim J.Y., Kim J.S., Hwang D.K., Im S. (2016). Black phosphorus–zinc oxide nanomaterial heterojunction for p–n diode and junction field-effect transistor. Nano Lett..

[2] Yang Z., Kim C., Lee K.Y., Lee M., Appalakondaiah S., Ra C.H., Watanabe K., Taniguchi T., Cho K., Hwang E. (2019). A Fermi-level-pinning-free 1D electrical contact at the intrinsic 2D MoS_2_–metal junction. Adv. Mater..

[3] Zhao D., Chen Y., Jiang W., Wang X., Liu J., Huang X., Han S., Lin T., Shen H., Wang X. (2021). Gate-tunable photodiodes based on mixed-dimensional Te/MoTe_2_ van der Waals heterojunctions. Adv. Electron. Mater..

[4] Chen L., Xue F., Li X., Huang X., Wang L., Kou J., Wang Z.L. (2016). Strain-gated field effect transistor of a MoS_2_–ZnO 2D–1D hybrid structure. ACS Nano.

[5] Li Y., Huang L., Li B., Wang X., Zhou Z., Li J., Wei Z. (2016). Co-Nucleus 1D/2D Heterostructures with Bi_2_S_3_ Nanowire and MoS_2_ Monolayer: One-Step Growth and Defect-Induced Formation Mechanism. ACS Nano.

[6] Hao G., Xiao J., Hao Y., Zhou G., Zhu H., Gao H., Xu Z., Zhao Z., Miao L., Li J. (2023). Van der waals epitaxial growth of mixed-dimensional 1D/2D heterostructures with tellurium nanowires and transition metal dichalcogenide nanosheets for nonlinear optical applications. Mater. Today Phys..

[7] Sun G., Li B., Li J., Zhang Z., Ma H., Chen P., Zhao B., Wu R., Dang W., Yang X. (2019). Direct van Der Waals Epitaxial Growth of 1D/2D Sb_2_Se_3_/WS_2_ Mixed-Dimensional p-n Heterojunctions. Nano Res..

[8] Um D.S., Lee Y., Lim S., Park S., Lee H., Ko H. (2016). High-performance MoS_2_/CuO nanosheet-on-one-dimensional heterojunction photodetectors. ACS Appl. Mater. Interfaces.

[9] Tang T., Li Z., Cheng Y.F., Xu K., Xie H.G., Wang X.X., Hu X.Y., Yu H., Zhang B.Y., Tao X.W. (2023). Single-Step Growth of p-type 1D Se/2D GeSexOy Heterostructures for Optoelectronic NO_2_ Gas Sensing at Room Temperature. J. Mater. Chem. A.

[10] Geng Y., Xu J., Bin Che Mahzan M.A., Lomax P., Saleem M.M., Mastropaolo E., Cheung R. (2022). Mixed Dimensional ZnO/WSe_2_ Piezo-gated Transistor with Active Millinewton Force Sensing. ACS Appl. Mater. Interfaces.

[11] Ni Y., Zhang S., Sun L., Liu L., Wei H., Xu Z., Xu W., Xu W. (2021). A low-dimensional hybrid pin heterojunction neuromorphic transistor with ultra-high UV sensitivity and immediate switchable plasticity. Appl. Mater. Today.

[12] Zhao Y., Fan L.L., Zhang Y., Zhao H., Li X., Li Y., Wen L., Yan Z., Huo Z. (2015). Hyper-Branched Cu@ Cu_2_O Coaxial Nanowires Mesh Electrode for Ultra-Sensitive Glucose Detection. ACS Appl. Mater. Interfaces.

[13] Yesilbag Y.O., Tuzluca Yesilbag F.N., Huseyin A., Tuzluca M., Ismail I., Ertugrul M. (2021). Synthesis and Characterization of Graphene/Carbon Nanotube Hybrid: Effects of Ni Catalyst Thickness and H_2_ Flow Rate on Growth and Morphological Structure. J. Mater. Sci. Mater. Electron..

[14] Zou A.L., Hu L.Z., Qiu Y., Cao G.Y., Yu J.J., Wang L.N., Zhang H.Q., Yin B., Xu L.L. (2015). High Performance of 1-D ZnO Microwire with Curve-Side Hexagon as Ethanol Gas Sensor. J. Mater. Sci. Mater. Electron..

[15] Alsultany F.H., Majdi H.S., Abd H.R., Hassan Z., Ahmed N.M. (2019). Catalytic Growth of 1D ZnO Nanoneedles on Glass Substrates Through Vapor Transport. J. Electron. Mater..

[16] Gong Y., Li B., Ye G., Yang S., Zou X., Lei S., Jin Z., Bianco E., Vinod S., Yakobson B.I. (2017). Direct Growth of MoS_2_ Single Crystals on Polyimide Substrates. 2D Mate.

[17] Dai C., Li B., Li J., Zhao B., Wu R., Ma H., Duan X. (2020). Controllable Synthesis of NiS and NiS_2_ Nanoplates by Chemical Vapor Deposition. Nano Res..

[18] Gao Z., Ji Q., Shen P.-C., Han Y., Leong W.S., Mao N., Zhou L., Su C., Niu J., Ji X. (2018). In Situ-Generated Volatile Precursor for CVD Growth of a Semimetallic 2D Dichalcogenide. Acs Appl. Mater. Interfaces.

[19] Jin C.Q., Ge C.H., Xu G., Wei Y.X., Ding Q.P., Zhu M., Duan H.B. (2015). Controllable Synthesis and Cathodoluminescent Property of 1D Wurtzite ZnS Nanostructures. J. Alloys Compd..

[20] Jin C., Wei Y., Peterson G., Zhu K., Jian Z. (2017). Controllable Synthesis and Defect-Dependent Photoluminescence Properties of In_2_O_3_ Nanostructures Prepared by PVD. Mater. Res. Express.

[21] Dontsova T.A., Nagirnyak S.V., Zhorov V.V., Yasiievych Y.V. (2017). SnO_2_ Nanostructures: Effect of Processing Parameters on Their Structural and Functional Properties. Nanoscale Res. Lett..

[22] Lan C., Dong R., Zhou Z., Shu L., Li D., Yip S., Ho J.C. (2017). Large-Scale Synthesis of Freestanding Layer-Structured PbI_2_ and MAPbI_3_ Nanosheets for High-Performance Photodetection. Adv. Mater..

[23] Xia J., Li X.-Z., Huang X., Mao N., Zhu D.-D., Wang L., Xu H., Meng X.-M. (2016). Physical Vapor Deposition Synthesis of Two-Dimensional Orthorhombic SnS Flakes with Strong Angle/Temperature-Dependent Raman Responses. Nanoscale.

[24] Bhorkar K., Sygellou L., Cathelinaud M., Ren D., Adam J.L., Yannopoulos S.N. (2022). Band alignment and optical properties of 1D/2D Sb2Se3/PtSe2 heterojunctions. ACS Appl. Electron. Mater..

[25] Xiao X., Zheng C., Cai S., Chen W., Li W., Guo Q. (2020). Optimization of nonlinear optical response of one-dimensional nanostructured sodium titanate through morphological control. Nano.

[26] Ma M., Zhe T., Ma Y., Wang Z., Chen Q., Wang J. (2018). Highly Sensitive and Reproducible Non-Enzymatic Glucose Sensor Fabricated by Drop-Casting Novel Nanocomposite with 3D Architecture and Tailorable Properties Prepared in Controllable Way. Talanta.

[27] Wei Z., Hsu C., Almakrami H., Lin G., Hu J., Jin X., Agar E., Liu F. (2019). Ultra-high-aspect-ratio vertically aligned 2D MoS_2_-1D TiO_2_ nanobelt heterostructured forests for enhanced photoelectrochemical performance. Electrochim. Acta.

[28] Xu K., Yang Y., Yu T., Yuan C. (2018). WO_3_ Nanofibers Anchored by Porous NiCo_2_O_4_ Nanosheets for Xylene Detection. Ceram. Int..

[29] Lee Y.T., Jeon P.J., Han J.H., Ahn J., Lee H.S., Lim J.Y., Choi W.K., Song J.D., Park M.C., Im S. (2017). Mixed-Dimensional 1D ZnO–2D WSe_2_ van der Waals Heterojunction Device for Photosensors. Adv. Funct. Mater..

[30] Huo J., Zou G., Xiao Y., Sun T., Feng B., Shen D., Lin L., Wang W., A Z., Liu L. (2023). High performance 1D–2D CuO/MoS_2_ photodetectors enhanced by femtosecond laser-induced contact engineering. Mater. Horiz..

[31] Tao J.J., Jiang J., Zhao S.N., Zhang Y., Li X.-X., Fang X., Wang P., Hu W., Lee Y.H., Lu H.-L. (2021). Fabrication of 1D Te/2D ReS_2_ mixed-dimensional van der waals p-n heterojunction for high-performance phototransistor. ACS Nano.

[32] Jahangir I., Wilson A., Uddin M.A., Chandrashekhar M.V.S., Koley G. Oxygen plasma treated graphene/InN nanowire heterojunction based sensors for toxic gas detection. Proceedings of the 2016 IEEE Sensors.

[33] Kim J.H., Sakaguchi I., Hishita S., Suzuki T.T., Saito N. (2022). Au-decorated 1D SnO_2_ nanowire/2D WS_2_ nanosheet composite for CO gas sensing at room temperature in self-heating mode. Chemosensors.

[34] Seo J., Nam S.H., Lee M., Kim J.-Y., Kim S.G., Park C., Seo D.-W., Kim Y.L., Kim S.S., Kim U.J. (2022). Gate-controlled gas sensor utilizing 1D–2D hybrid nanowires network. IScience.

[35] Geng Y. (2022). Development and Characterisation of an Integrated 1D–2D Hybrid Field-Effect Transistor for Force-Sensing Applications. Ph.D. Thesis.

[36] Zhou M., Zhao Y., Gu X., Zhang Q., Zhang J., Jiang M., Lu S. (2023). Realize low-power artificial photonic synapse based on (Al, Ga) N nanowire/graphene heterojunction for neuromorphic computing. APL Photonics.

[37] Jiang L.Y., Hu R., Wang A.J., Mei L.P., Feng J.J. (2023). 1D/2D SnO_2_/SnS_2_ heterojunctions coupling with PtPd/CeO_2_ heterostructured nanozyme for ultrasensitive PEC apatasensing of lincomycin. Sens. Actuators B Chem..

[38] Xu M., Tang B., Lu Y., Zhu C., Lu Q., Zheng L., Zhang J., Han N., Fang W., Guo Y. (2021). Machine Learning Driven Synthesis of Few-Layered WTe_2_ with Geometrical Control. J. Am. Chem. Soc..

[39] Yang L., Yuan X., Liu R., Wu P., Zhong Y., Zhu F., Chang W. (2022). Intrinsic Properties of Metallic Edge States in MoS_2_ Nanobelt. J. Mater. Sci. Mater. Electron..

[40] Tuzluca F.N., Yesilbag Y.O., Ertugrul M. (2018). Synthesis of In_2_O_3_ Nanostructures with Different Morphologies as Potential Supercapacitor Electrode Materials. Appl. Surf. Sci..

[41] Zhao Y., Zuo X., Guo Y., Huang H., Zhang H., Wang T., Wen N., Chen H., Cong T., Muhammad J. (2021). Structural Engineering of Hierarchical Aerogels Comprised of Multi-Dimensional Gradient Carbon Nanoarchitectures for Highly Efficient Microwave Absorption. Nano-Micro Lett..

[42] Zou J., Zhang X., Xu C., Zhao J., Zhu Y.T., Li Q. (2017). Soldering Carbon Nanotube Fibers by Targeted Electrothermal-Induced Carbon Deposition. Carbon.

[43] Ince G.O., Armagan E., Erdogan H., Buyukserin F., Uzun L., Demirel G. (2013). One-dimensional surface-imprinted polymeric nanotubes for specific biorecognition by initiated chemical vapor deposition (iCVD). ACS Appl. Mater. Interfaces.

[44] Kang S.W., Deshmukh P.R., Sohn Y., Shin W.G. (2019). Plasmonic Gold Sensitization of ZnO Nanowires for Solar Water Splitting. Mater. Today Commun..

[45] Shaukat S., Khaleeq-ur-Rahman M., Dildar I.M., Jamil H., Binions R. (2019). One Dimensional (1-D) Signatures of Nanopillars and Nanowires in Niobium Doped Zinc Oxide (NZO) Thin Films Prepared by Aerosol Assisted Chemical Vapour Deposition (AACVD). Ceram. Int..

[46] Wu S., Zeng X., Wang W., Zeng Y., Hu Y., Yin S., Lu J., Zhou G. (2018). The Morphological Control of MoS_2_ Films Using a Simple Model under Chemical Vapor Deposition. Thin Solid Film..

[47] Bosi M., Rotunno E. (2019). Direct Synthesis of Few-Layer MoS_2_ on Silica Nanowires by Chemical Vapor Deposition. J. Nanosci. Nanotechnol..

[48] Liu X., Chen J., Hu Y., Pan Q., Du X., Zhong H., Zhang T., Jiang C., Ma B. (2023). CVD Growth of the Centimeter-Scale Continuous 2D MoS_2_ Film by Modulating the Release of Mo Vapor with Adjusting the Particle Size of Al_2_O_3_ Microsphere. Chem. Phys. Lett..

[49] Hafeez M., Gan L., Li H., Ma Y., Zhai T. (2016). Large-Area Bilayer ReS_2_ Film/Multilayer ReS_2_ Flakes Synthesized by Chemical Vapor Deposition for High Performance Photodetectors. Adv. Funct. Mater..

[50] Guo Y., Kang L., Yu S., Yang J., Qi X., Zhang Z., Liu Z. (2021). CVD Growth of Large-scale and Highly Crystalline 2D Chromium Telluride Nanoflakes. Chem. Nano. Mat..

[51] Meng L., Xu C., Li H., Wang X., Yan X. (2019). Controlled Synthesis and Frictional Properties of 2D MoTe_2_ via Chemical Vapor Deposition. Chem. Phys. Lett..

[52] Wang S., Wang G., Yang X., Yang H., Zhu M., Zhang S., Peng G., Li Z. (2020). Synthesis of Monolayer MoSe_2_ with Controlled Nucleation via Reverse-Flow Chemical Vapor Deposition. Nanomaterials.

[53] Qian Y., Seo S., Jeon I., Lin H., Okawa S., Zheng Y., Shawky A., Anisimov A., Kauppinen E., Kong J. (2020). MoS_2_-Carbon Nanotube Heterostructure as Efficient Hole Transporters and Conductors in Perovskite Solar Cells. Appl. Phys. Express.

[54] Jin C., Peterson G., Zhu K., Jian Z., Wei Y., Ge C. (2017). One-Step Synthesis and Scale-Dependent Luminescence Properties of 1D Zn_0.9_Cd_0.1_S Nanostructures Prepared by PVD. J. Phys. D-Appl. Phys..

[55] Xie X., Su Z., Huang D., Yang C., Wang Y., Jiang D., Huang Q. (2020). Synthesis and Growth Mechanism of SiC/SiO_2_ Nanochains by Catalyst-Free Thermal Evaporation Method in Ar/CO Atmosphere. Trans. Nonferrous Met. Soc. China.

[56] Lu J., Zeng X., Liu H., Zhang W., Zhang Y. (2012). Controlled Growth and Photoluminescence of One-Dimensional and Platelike ZnS Nanostructures. Appl. Surf. Sci..

[57] Shariati M., Ghafouri V. (2014). In_2_O_3_-ZnO Heterostructure Development in Electrical and Photoluminescence Properties of In_2_O_3_ 1-D Nanostructures. Int. J. Mod. Phys. B.

[58] Wei X., Wang S., Zhang N., Li Y., Tang Y., Jing H., Lu J., Xu Z., Xu H. (2023). Single-Orientation Epitaxy of Quasi-1D Tellurium Nanowires on M-Plane Sapphire for Highly Uniform Polarization Sensitive Short-Wave Infrared Photodetection. Adv. Funct. Mater..

[59] Li S., Zhang H., Ruan H., Cheng Z., Yao Y., Zhuge F., Zhai T. (2023). Programmable nucleation and growth of ultrathin tellurium nanowires via a pulsed physical vapor deposition design. Adv. Funct. Mater..

[60] Hanson E.D., Lajaunie L., Hao S., Myers B.D., Shi F., Murthy A.A., Wolverton C., Arenal R., Dravid V.P. (2017). Systematic Study of Oxygen Vacancy Tunable Transport Properties of Few-Layer MoO_3-x_ Enabled by Vapor-Based Synthesis. Adv. Funct. Mater..

[61] Arash A., Ahmed T., Rajan A.G., Walia S., Rahman F.A., Mazumder A., Ramanathan R., Sriram S., Bhaskaran M., Mayes E. (2019). Large-Area Synthesis of 2D MoO_3-x_ for Enhanced Optoelectronic Applications. 2D Mater..

[62] Yao K., Chen P., Zhang Z., Li J., Ai R., Ma H., Zhao B., Sun G., Wu R., Tang X. (2018). Synthesis of Ultrathin Two-Dimensional Nanosheets and van Der Waals Heterostructures from Non-Layered Gamma-CuI. Npj 2D Mater. Appl..

[63] Suleiman A.A., Huang P., Jin B., Jiang J., Zhang X., Zhou X., Zhai T. (2020). Space-Confined Growth of 2D InI Showing High Sensitivity in Photodetection. Adv. Electron. Mater..

[64] Zhou J., Shi J., Zeng Q., Chen Y., Niu L., Liu F., Yu T., Suenaga K., Liu X., Lin J. (2018). InSe Monolayer: Synthesis, Structure and Ultra-High Second-Harmonic Generation. 2D Mater..

[65] Sharma R., Birojud R.K., Sinai O., Cohen H., Sahoo K.R., Artel V., Alon H., Levi A., Subrahmanyam A., Theis W. (2018). Vapour Transport Deposition of Fluorographene Oxide Films and Electro-Optical Device Applications. Appl. Mater. Today.

[66] Chanana A., Lotfizadeh N., Condori Quispe H.O., Gopalan P., Winger J.R., Blair S., Nahata A., Deshpande V.V., Scarpulla M.A., Sensale-Rodriguez B. (2019). Manifestation of Kinetic Inductance in Terahertz Plasmon Resonances in Thin-Film Cd_3_As_2_. ACS Nano.

[67] Zhou Y., Han L., Song Q., Gao W., Yang M., Zheng Z., Huang L., Yao J., Li J. (2022). Hybrid 1D/2D heterostructure with electronic structure engineering toward high-sensitivity and polarization-dependent photodetector. Sci. China (Mater.).

[68] Chen Y.X., Tang Q.L., Chen J.H. (2008). Dendritic and epitaxial growths of ZnO particle–rod and rod–rod nanostructures with quasi-one-dimensional and two-dimensional configurations. Mater. Lett..

[69] Yu L., Li D., Yue M. (2007). Fabrication and characterization of the photoluminescent properties of Tb3+ doped one-dimensional GdPO4 nanorods. Mater. Lett..

[70] Ahmad K., Kim H. (2022). Synthesis of MoS_2_/WO_3_ Hybrid Composite for Hydrazine Sensing Applications. Mater. Sci. Semicond. Process..

[71] Xu M., Zhao J. (2018). Facile Synthesis of 1D/2D Core–Shell Structured Sb_2_S_3_@ MoS_2_ Nanorods with Enhanced Photocatalytic Performance. Electron. Mater. Lett..

[72] Xu F., Zhu B., Cheng B., Yu J., Xu J. (2018). 1D/2D TiO_2_/MoS_2_ hybrid nanostructures for enhanced photocatalytic CO_2_ reduction. Adv. Opt. Mater..

[73] Wang W., Wang W., Meng Y., Quan Q., Lai Z., Li D., Xie P., Yip S., Kang X., Bu X. (2022). Mixed-Dimensional Anti-ambipolar Phototransistors Based on 1D GaAsSb/2D MoS_2_ Heterojunctions. ACS Nano.

[74] Hou Y., Wang K., Yang D., Jiang Y., Yennawar N.H., Wang K., Sanghadasa M., Wu C., Priya S. (2019). Enhanced performance and stability in DNA-perovskite heterostructure-based solar cells. ACS Energy Lett..

[75] Wang Z., Jingjing Q., Wang X., Zhang Z., Chen Y., Huang X., Huang W. (2018). Two-dimensional light-emitting materials: Preparation, properties and applications. Chem. Soc. Rev..

[76] Guo N., Gong F., Liu J., Jia Y., Zhao S., Liao L., Su M., Fan Z., Chen X., Lu W. (2017). Hybrid WSe_2_–In_2_O_3_ phototransistor with ultrahigh detectivity by efficient suppression of dark currents. ACS Appl. Mater. Interfaces.

[77] Chang C., Chen W., Chen Y., Chen Y., Chen Y., Ding F., Fan C., Fan H.J., Fan Z., Gong C. (2021). Recent progress on two-dimensional materials. Acta Phys. Chim. Sin.

[78] Luo L.B., Fu C. (2020). Phototransistors based on metal oxide semiconductors. Semicond. Met. Oxide Thin-Film. Transistors.

[79] Sahatiya P., Badhulika S. (2017). Discretely distributed 1D V_2_O_5_ nanowires over 2D Mo_2_ nanoflakes for an enhanced broadband flexible photodetector covering the ultraviolet to near infrared region. J. Mater. Chem. C..

[80] Ouyang W., Teng F., He J.H., Fang X. (2019). Enhancing the photoelectric performance of photodetectors based on metal oxide semiconductors by charge-carrier engineering. Adv. Funct. Mater..

[81] Tao Z., Liu X., Lei W., Chen J. (2018). High sensitive solar blind phototransistor based on ZnO nanorods/IGZO heterostructure annealed by laser. Mater. Lett..

[82] Xue F., Yang L., Chen M., Chen J., Yang X., Wang L., Chen L., Pan C., Wang Z.L. (2017). Enhanced photoresponsivity of the MoS_2_-GaN heterojunction diode via the piezo-phototronic effect. NPG Asia Mater..

[83] Fu N., Zhang J., He Y., Lv X., Guo S., Wang X., Zhao B., Chen G., Wang L. (2023). High-Sensitivity 2D MoS_2_/1D MWCNT Hybrid Dimensional Heterostructure Photodetector. Sensors.

[84] Yang Z., Liu X., Zou X., Wang J., Ma C., Jiang C., Ho J.C., Pan C., Xiao X., Xiong J. (2017). Performance Limits of the Self-Aligned Nanowire Top-Gated MoS_2_ Transistors. Adv. Funct. Mater..

[85] Lin P., Zhu L., Li D., Xu L., Wang Z.L. (2018). Tunable WSe_2_–CdS mixed-dimensional van der Waals heterojunction with a piezo-phototronic effect for an enhanced flexible photodetector. Nanoscale.

[86] Miao J., Hu W., Guo N., Lu Z., Liu X., Liao L., Chen P., Jiang T., Wu S., Ho J.C. (2015). High-responsivity graphene/InAs nanowire heterojunction near-infrared photodetectors with distinct photocurrent on/off ratios. Small.

[87] Yi H., Yang H., Ma C., Ma Y., Ye Q., Lu J., Wang W., Zheng Z., Deng Z., Zou Y. (2023). Multilayer SnS_2_/few-layer MoS_2_ heterojunctions with in-situ floating photogate toward high-performance photodetectors and optical imaging application. Sci. China Mater..

[88] Zhang X.L., Li J., Leng B., Yang L., Song Y.D., Feng S.Y., Feng L.Z., Liu Z.T., Fu Z.W., Jiang X. (2023). High-performance ultraviolet-visible photodetector with high sensitivity and fast response speed based on MoS_2_-on-ZnO photogating heterojunction. Tungsten.

[89] Zhao S., Dong B., Wang H., Wang H., Zhang Y., Han Z.V., Zhang H. (2020). In-plane anisotropic electronics based on low-symmetry 2D materials: Progress and prospects. Nanoscale Adv..

[90] Ying H., Li X., Wu Y., Yao Y., Xi J., Su W., Jin C., Xu M., He Z., Zhang Q. (2019). High-performance ultra-violet phototransistors based on CVT-grown high quality SnS_2_ flakes. Nanoscale Adv..

[91] Qin J., Qiu G., Jian J., Zhou H., Yang L., Charnas A., Zemlyanov D.Y., Xu C.-Y., Xu X., Wu W. (2017). Controlled growth of a large-size 2D selenium nanosheet and its electronic and optoelectronic applications. ACS Nano.

[92] Qin J., Yan H., Qiu G., Si M., Miao P., Duan Y., Shao W., Zhen L., Xu C., Ye P.D. (2019). Hybrid dual-channel phototransistor based on 1D t-Se and 2D ReS_2_ mixed-dimensional heterostructures. Nano Res..

[93] Han L., Yang M., Wen P., Gao W., Huo N., Li J. (2021). A high performance self-powered photodetector based on a 1D Te–2D WS_2_ mixed-dimensional heterostructure. Nanoscale Adv..

[94] Chen X., Jiang B., Wang D., Li G., Wang H., Wang H., Wang F., Wang P., Liao L., Wei Z. (2021). Gate-tunable the interface properties of GaAs–WSe_2_ (1D–2D) vdWs heterojunction for high-responsivity, self-powered photodetector. Appl. Phys. Lett..

[95] Jang J., Lee Y., Yoon J.Y., Yoon H.H., Koo J., Choe J., Jeon S., Sung J., Park J., Lee W.C. (2018). One-dimensional assembly on two-dimensions: AuCN nanowire epitaxy on graphene for hybrid phototransistors. Nano Lett..

[96] Yang W., Hu K., Teng F., Weng J., Zhang Y., Fang X. (2018). High-performance silicon-compatible large-area UV-to-visible broadband photodetector based on integrated lattice-matched type II Se/n-Si heterojunctions. Nano Lett..

[97] Ghoshal D., Yoshimura A., Gupta T., House A., Basu S., Chen Y., Wang T., Yang Y., Shou W., Hachtel J.A. (2018). Theoretical and experimental insight into the mechanism for spontaneous vertical growth of ReS_2_ nanosheets. Adv. Funct. Mater..

[98] Zheng L., Hu K., Teng F., Fang X. (2017). Novel UV–visible photodetector in photovoltaic mode with fast response and ultrahigh photosensitivity employing Se/TiO_2_ nanotubes heterojunction. Small.

[99] Schaper N., Alameri D., Kim Y., Thomas B., McCormack K., Chan M., Divan R., Gosztola D.J., Liu Y., Kuljanishvili I. (2021). Controlled fabrication of quality ZnO NWs/CNTs and ZnO NWs/Gr heterostructures via direct two-step CVD method. Nanomaterials.

[100] Zhang J., Liu Y., Zhang X., Ma Z., Li J., Zhang C., Shaikenova A., Renat B., Liu B. (2020). High-performance ultraviolet-visible light-sensitive 2D-MoS_2_/1D-ZnO heterostructure photodetectors. Chem. Sel..

[101] Veeralingam S., Badhulika S. (2020). 2D-SnSe_2_ nanoflakes on paper with 1D-NiO gate insulator based MISFET as multifunctional NIR photo switch and flexible temperature sensor. Mater. Sci. Semicond. Process..

[102] Boruah B.D., Ferry D.B., Mukherjee A., Misra A. (2015). Few-layer graphene/ZnO nanowires based high performance UV photodetector. Nanotechnology.

[103] Noothongkaew S., Thumthan O., An K.S. (2018). Minimal layer graphene/TiO_2_ nanotube membranes used for enhancement of UV photodetectors. Mater. Lett..

[104] Shin J., Yoo H. (2023). Photogating effect-driven photodetectors and their emerging applications. Nanomaterials.

[105] Wang Y., Du C., Li P., Yang Y., Xiao Y., Ge T., Jiang X., Liu Y., Gao H., Li K. (2024). Photodetectors Based on ZrS_3_/MoS_2_ Heterostructures. ACS Appl. Mater. Interfaces.

[106] Jana S., Pal S., Bhaktha S.B.N., Ray S.K. (2022). Synergistic effects of plasmonic au nanoislands on a MoSe_2_ Nanoflake/ZnO nanorod heterostructure for an enhanced broadband photoresponse. ACS Appl. Nano Mater..

[107] Tien L.-C., Shih Y.-C., Chen C.-Y., Huang Y.-T., Chen R.-S. (2021). Broadband photodetectors based on layered 1D GaTe nanowires and 2D GaTe nanosheets. J. Alloys Compd..

[108] Wang K., Chen F., Belev G., Kasap S., Karim K.S. (2009). Lateral metal-semiconductor-metal photodetectors based on amorphous selenium. Appl. Phys. Lett..

[109] Schwierz F., Pezoldt J., Granzner R. (2015). Two-dimensional materials and their prospects in transistor electronics. Nanoscale.

[110] Wang R., Su X., Bulla D., Wang T., Gai X., Yang Z., Madden S., Luther-Davies B. (2015). Identifying the best chalcogenide glass compositions for the application in mid-infrared waveguides. Int. Semin. Photonics Opt. Its Appl. (ISPhOA 2014)..

[111] Zhou K., Dai K., Liu C., Shen C. (2020). Flexible conductive polymer composites for smart wearable strain sensors. SmartMat.

[112] Shang H., Chen H., Dai M., Hu Y., Gao F., Yang H., Xu B., Zhang S., Tan B., Zhang X. (2020). A mixed-dimensional 1D Se–2D InSe van der Waals heterojunction for high responsivity self-powered photodetectors. Nanoscale Horiz..

[113] Chen M., Hu X., Li K., Sun J., Liu Z., An B., Zhou X., Liu Z. (2020). Self-assembly of dendritic-lamellar MXene/Carbon nanotube conductive films for wearable tactile sensors and artificial skin. Carbon.

[114] Amjadi M., Kyung K.U., Park I., Sitti M. (2016). Stretchable, skin-mountable, and wearable strain sensors and their potential applications: A review. Adv. Funct. Mater..

[115] Choi S., Lee H., Ghaffari R., Hyeon T., Kim D.H. (2016). Recent advances in flexible and stretchable bio-electronic devices integrated with nanomaterials. Adv. Mater..

[116] Rim Y.S. (2020). Review of metal oxide semiconductors-based thin-film transistors for point-of-care sensor applications. J. Inf. Disp..

[117] Hammock M.L., Chortos A., Tee B.C.K., Tok J.B.H., Bao Z. (2013). 25th Anniversary Article: The Evolution of Electronic Skin (E-Skin): A Brief History, Design Considerations, and Recent Progress. Adv. Mater.

[118] Zang Y., Zhang F., Di C., Zhu D. (2015). Advances of flexible pressure sensors toward artificial intelligence and health care applications. Mater. Horiz..

[119] Wang L., Zhang M., Yang B., Tan J., Ding X., Li W. (2021). Recent advances in multidimensional (1D, 2D, and 3D) composite sensors derived from MXene: Synthesis, structure, application, and perspective. Small Methods.

[120] Yan C., Wang J., Kang W., Cui M., Wang X., Foo C.Y., Chee K.J., Lee P.S. (2014). Highly stretchable piezoresistive graphene–nanocellulose nanopaper for strain sensors. Adv. Mater..

[121] Zhao J., He C., Yang R., Shi Z., Cheng M., Yang W., Xie G., Wang D., Shi D., Zhang G. (2012). Ultra-sensitive strain sensors based on piezoresistive nanographene films. Appl. Phys. Lett..

[122] Zhao J., Wang G., Yang R., Lu X., Cheng M., He C., Xie G., Meng J., Shi D., Zhang G. (2015). Tunable piezoresistivity of nanographene films for strain sensing. ACS Nano.

[123] Dang V.Q., Kim D.I., Kim B.Y., Hwang B.U., Jang M., Shin K.S., Kim S.W., Lee N.E. (2014). Piezoelectric coupling in a field-effect transistor with a nanohybrid channel of ZnO nanorods grown vertically on graphene. Nanoscale.

[124] Sun Q., Seung W., Kim B.J., Seo S., Kim S., Cho J.H. (2015). Active matrix electronic skin strain sensor based on piezopotential-powered graphene transistors. Adv. Mater..

[125] Zhu J., Zhou X., Jing L., Hua Q., Hu W., Wang Z.L. (2019). Piezotronic effect modulated flexible AlGaN/GaN high-electron-mobility transistors. ACS Nano.

[126] Jo S., Kim I., Byun J., Jayababu N., Kim D. (2021). Boosting a power performance of a hybrid nanogenerator via frictional heat combining a triboelectricity and thermoelectricity toward advanced smart sensors. Adv. Mater. Technol..

[127] Baxter L.K. (1997). Capacitive Sensors: Design and Applications.

[128] Rivadeneyra A., López-Villanueva J.A. (2020). Recent advances in printed capacitive sensors. Micromachines.

[129] Fiorillo A.S., Critello C.D., Pullano S.A. (2018). Theory, technology and applications of piezoresistive sensors: A review. Sens. Actuators A Phys..

[130] Liu X., Mwangi M., Li X.J., O’Brien M., Whitesides G.M. (2011). based piezoresistive MEMS sensors. Lab A Chip.

[131] Jiang C., Li X., Yao Y., Lan L., Shao Y., Zhao F., Ying Y., Ping J. (2019). A multifunctional and highly flexible triboelectric nanogenerator based on MXene-enabled porous film integrated with laser-induced graphene electrode. Nano Energy.

[132] Seyedin S., Zhang P., Naebe M., Qin S., Chen J., Wang X., Razal J.M. (2019). Textile strain sensors: A review of the fabrication technologies, performance evaluation and applications. Mater. Horiz..

[133] Zhang C., Sun J., Lu Y., Liu J. (2021). Nanocrack-based strain sensors. J. Mater. Chem. C.

[134] Jiang C., Li X., Ying Y., Ping J. (2020). A multifunctional TENG yarn integrated into agrotextile for building intelligent agriculture. Nano Energy.

[135] Chen P.W., Chung D.D.L. (1996). Concrete as a new strain/stress sensor. Compos. Part B Eng..

[136] Jian M., Xia K., Wang Q., Yin Z., Wang H., Wang C., Xie H., Zhang M., Zhang Y. (2017). Flexible and highly sensitive pressure sensors based on bionic hierarchical structures. Adv. Funct. Mater..

[137] Chen Z., Lu C. (2005). Humidity sensors: A review of materials and mechanisms. Sens. Lett..

[138] Wang K., Wei W., Lou Z., Zhang H., Wang L. (2019). 1D/2D heterostructure nanofiber flexible sensing device with efficient gas detectivity. Appl. Surf. Sci..

[139] Shimizu Y., Takao Y., Egashira M. (1988). Detection of freshness of fish by a semiconductive Ru/TiO_2_ sensor. J. Electrochem. Soc..

[140] Hashemi S.A., Bahrani S., Mousavi S.M., Omidifar N., Behbahan N.G.G., Arjmand M., Ramakrishna S., Dimiev A.M., Lankarani K.B., Moghadami M. (2022). Antibody mounting capability of 1D/2D carbonaceous nanomaterials toward rapid-specific detection of SARS-CoV-2. Talanta.

[141] Ama O., Sadiq M., Johnson M., Zhang Q., Wang D. (2020). Novel 1D/2D KWO/Ti_3_C_2_Tx nanocomposite-based acetone sensor for diabetes prevention and monitoring. Chemosensors.

[142] Shoyama M., Hashimoto N. (2003). Effect of poly ethylene glycol addition on the microstructure and sensor characteristics of SnO_2_ thin films prepared by sol–gel method. Sens. Actuators B Chem..

[143] Paghi A., Mariani S., Barillaro G. (2023). 1D and 2D Field Effect Transistors in Gas Sensing: A Comprehensive Review. Small.

[144] Alagh A., Annanouch F.E., Umek P., Bittencourt C., Sierra-Castillo A., Haye E., Colomer J.F., Llobet E. (2021). CVD growth of self-assembled 2D and 1D WS_2_ nanomaterials for the ultrasensitive detection of NO_2_. Sens. Actuators B Chem..

[145] Zhao J., Zhang G.Y., Shi D.X. (2013). Review of graphene-based strain sensors. Chin. Phys. B.

[146] Ball C., Westhorpe R.N. (2009). Direct blood pressure monitoring. Anaesth. Intensive Care.

[147] Petersen K.E. (1982). Silicon as a mechanical material. Proc. IEEE.

[148] Bernstein D., Godfrey C., Klein A., Shimmin W. (1968). Research on manganin pressure transducers. Behaviour of Dense Media under High Dynamic Pressures.

[149] Wang Y., Wang L., Yang T., Li X., Zang X., Zhu M., Wang K., Wu D., Zhu H. (2014). Wearable and highly sensitive graphene strain sensors for human motion monitoring. Adv. Funct. Mater..

[150] Li X., Koh K.H., Xue J., So C.H., Xiao N., Tin C., Wai K., Lai C. (2022). 1D–2D nanohybrid-based textile strain sensor to boost multiscale deformative motion sensing performance. Nano Res..

[151] Lee H.J., Yang J.C., Choi J., Kim J., Lee G.S., Sasikala S.P., Lee G.H., Park S.H.K., Lee H.M., Sim J.Y. (2021). Hetero-dimensional 2D Ti_3_C_2_T x MXene and 1D graphene nanoribbon hybrids for machine learning-assisted pressure sensors. ACS Nano.

[152] Tosun M., Chan L., Amani M., Roy T., Ahn G.H., Taheri P., Carraro C., Ager J.W., Maboudian R., Javey A. (2016). Air-stable n-doping of WSe_2_ by anion vacancy formation with mild plasma treatment. ACS Nano.

[153] Liu B., Ma Y., Zhang A., Chen L., Abbas A.N., Liu Y., Shen C., Wan H., Zhou C. (2016). High-performance WSe_2_ field-effect transistors via controlled formation of in-plane heterojunctions. ACS Nano.

[154] Zhang R., Drysdale D., Koutsos V., Cheung R. (2017). Controlled layer thinning and p-type doping of WSe_2_ by vapor XeF_2_. Adv. Funct. Mater..

[155] Zhang R., Koutsos V., Cheung R. (2016). Elastic properties of suspended multilayer WSe_2_. Appl. Phys. Lett..

[156] Sun Y., Ding Y., Xie D. (2021). Mixed-dimensional van der Waals heterostructures enabled optoelectronic synaptic devices for neuromorphic applications. Adv. Funct. Mater..

[157] Ilyas N., Wang J., Li C., Li D., Fu H., Gu D., Jiang X., Liu F., Jiang Y., Li W. (2022). Nanostructured materials and architectures for advanced optoelectronic synaptic devices. Adv. Funct. Mater..

[158] Khan R., Rahman N.U., Hayat M.F., Ghernaout D., Salih A.A.M., Ashraf G.A., Samad A., Mahmood M.A., Rahman N., Sohail M. (2024). Unveiling cutting-edge developments: Architectures and nanostructured materials for application in optoelectronic artificial synapses. Nanoscale.

[159] Shen W., Wang P., Wei G., Yuan S., Chen M., Su Y., Xu B., Li G. (2024). SiC@NiO Core–Shell Nanowire Networks-Based Optoelectronic Synapses for Neuromorphic Computing and Visual Systems at High Temperature. Small.

[160] O’Kelly C.J., Fairfield J.A., McCloskey D., Manning H.G., Donegan J.F., Boland J.J. (2016). Associative enhancement of time correlated response to heterogeneous stimuli in a neuromorphic nanowire device. Adv. Electron. Mater..

[161] Jana R., Ghosh S., Bhunia R., Chowdhury A. (2024). Recent developments in the state-of-the-art optoelectronic synaptic devices premised on 2D materials: A review. J. Mater. Chem. C.

[162] Hao R., Hu X., Luo L., Yang X., Feng Q., Li Y., Zhang Z. (2024). Two-dimensional Optoelectronic Memristive Device Realized by Ferroelectric Regulation. IEEE Electron Device Lett..

[163] Cao Y., Zhao C., Zhao T., Sun Y., Liu Z., Li X., Yin L., Gu J., Ren H., Geng X. (2023). Brain-like optoelectronic artificial synapses with ultralow energy consumption based on MXene floating-gates for emotion recognition. J. Mater. Chem. C.

[164] Chen Y., Zhang M., Li D., Tang Y., Ren H., Li J., Liang K., Wang Y., Wen L., Li W. (2023). Bidirectional synaptic phototransistor based on two-dimensional ferroelectric semiconductor for mixed color pattern recognition. ACS Nano.

[165] Yan S., Shi Y., Xiao Z., Zhou M., Yan W., Shen H., Hu D. (2012). Development of biosensors based on the one-dimensional semiconductor nanomaterials. J. Nanosci. Nanotechnol..

[166] Pohanka M., Leuchter J. (2017). Biosensors based on semiconductors, a review. Int. J. Electrochem. Sci..

[167] Yang Z., Ye Z., Zhao B., Zong X., Wang P. (2010). A rapid response time and highly sensitive amperometric glucose biosensor based on ZnO nanorod via citric acid-assisted annealing route. Phys. E Low-Dimens. Syst. Nanostruct..

[168] Xu L., Yang Q., Liu X., Liu J., Sun X. (2014). One-dimensional copper oxide nanotube arrays: Biosensors for glucose detection. RSC Adv..

[169] Zhou W.-H., Wang H.-H., Li W.-T., Guo X.-C., Kou D.-X., Zhou Z.-J., Meng Y.-N., Tian Q.-W., Wu S.-X. (2018). Gold nanoparticles sensitized ZnO nanorods arrays for dopamine electrochemical sensing. J. Electrochem. Soc..

[170] Jiang G., Yang R., Liu J., Liu H., Liu L., Wu Y., A. Y. (2022). Two-dimensional Ti2C MXene-induced photocurrent polarity switching photoelectrochemical biosensing platform for ultrasensitive and selective detection of soluble CD146. Sens. Actuators B Chem..

[171] Zhang Y., Feng D., Xu Y., Yin Z., Dou W., Habiba U.E., Pan C., Zhang Z., Mou H., Deng H. (2021). DNA-based functionalization of two-dimensional MoS_2_ FET biosensor for ultrasensitive detection of PSA. Appl. Surf. Sci..

[172] Manoharan A.K., Batcha M.I.K., Mahalingam S., Raj B., Kim J. (2024). Recent Advances in Two-Dimensional Nanomaterials for Healthcare Monitoring. ACS Sens..

